# Density Functional Theory (DFT)-Aided Structure Elucidation of Linear Diterpenes from the Irish Brown Seaweed *Bifurcaria bifurcata*

**DOI:** 10.3390/md19010042

**Published:** 2021-01-19

**Authors:** Vangelis Smyrniotopoulos, Daria Firsova, Howard Fearnhead, Laura Grauso, Alfonso Mangoni, Deniz Tasdemir

**Affiliations:** 1 School of Chemistry, National University of Ireland Galway, University Road, H91 TK33 Galway, Ireland; vsmy@hotmail.com (V.S.); dashafirsova63@gmail.com (D.F.); 2 Pharmacology and Therapeutics, School of Medicine, National University of Ireland Galway, University Road, H91 W2TY Galway, Ireland; howard.fearnhead@nuigalway.ie; 3 Dipartimento di Agraria, Università degli Studi di Napoli Federico II, 80055 Portici (NA), Italy; laura.grauso@unina.it; 4 Dipartimento di Farmacia, Università degli Studi di Napoli Federico II, 80131 Napoli, Italy; alfonso.mangoni@unina.it; 5 GEOMAR Centre for Marine Biotechnology (GEOMAR-Biotech), Research Unit Marine Natural Product Chemistry, GEOMAR Helmholtz Centre for Ocean Research Kiel, Am Kiel-Kanal 44, 24106 Kiel, Germany; 6 Faculty of Mathematics and Natural Sciences, Kiel University, Christian-Albrechts-Platz 4, 24118 Kiel, Germany

**Keywords:** *Bifurcaria bifurcata*, brown alga, seaweed, linear diterpene, DFT, anticancer activity

## Abstract

Brown alga *Bifurcaria bifurcata* is an extraordinarily rich source of linear (acylic) diterpenes with enormous structural diversity. As part of our interest into secondary metabolites of the Irish seaweeds, here we report four new acyclic diterpenes (**1**–**4**) and seven known terpenoids (**5**–**11**) from the CHCl_3_ extract of *B. bifurcata*. The planar structures of the new metabolites were elucidated by means of 1D and 2D NMR, HRMS, and FT-IR spectroscopy. Since linear diterpenes are highly flexible compounds, the assignment of their stereochemistry by conventional methods, e.g., NOESY NMR, is difficult. Therefore, we employed extensive quantum-mechanical prediction of NMR chemical shifts and optical rotation analyses to identify the relative and absolute configurations of the new compounds **1**–**4**. Several compounds moderately inhibited the human breast cancer cell line (MDA-MB-231) with IC_50_ values ranging from 10.0 to 33.5 μg/mL. This study not only demonstrates the vast capacity of the Irish *B. bifurcata* to produce highly oxygenated linear diterpenoids, but also highlights the potential of new methodologies for assignment of their stereogenic centers.

## 1. Introduction

*Bifurcaria* is a small genus within the brown algal family Sargassaceae (order Fucales). *Bifurcaria bifurcata* (Velley) R. Ross, also known as brown forking weed or brown tuning fork weed [1,2] is widespread on the seashores and tidepools of the Atlantic Ocean [1,3]. In Ireland, it is commonly found on the limestone areas of the Northwestern Ireland [1]. From a chemical point of view, *B. bifurcata* is reputed for being a rich source of linear diterpenes (LDs) that originate from geranylgeraniol, a C20 metabolite bearing four double bonds and five methyl functions [2,3]. Further substitutions and rearrangements, such as oxygenation, isomerization, unsaturation or formation of terminal ring systems (e.g.**,** furan), result in a highly diverse LD family. In general, LDs deriving from *B. bifurcata* have been divided into 3 subfamilies: (a) those with C-12 oxygenation, (b) those with C-13 oxygenation, and (c) the non-C-12/C-13 oxygenated diterpenes [3]. The diterpene composition of *B. bifurcata* has been reported to vary with the collection site and season, and several types of chemotypes have been reported [4,5,6]. These diterpenes are of ecological importance and considered as chemotaxonomical markers [5,6]. They also exhibit pharmacologically-relevant activities, such as antiprotozoal, anticancer, and neuroprotective [3,7,8,9,10,11].

Identification of the stereogenic centers within linear, highly flexible natural products is challenging. Our previous studies on Irish *B. bifurcata* reported several acyclic diterpenes belonging to the C-13 oxygenated LD series, e.g., eleganediol (**12**) [10]. In that work, we employed, for the first time for a LD, vibrational circular dichroism (VCD) spectroscopy joined with density functional theory (DFT) calculations and DP4 probability analyses to assign *S* absolute configuration for single (C-13) or two (C-7, C-13) stereogenic centers [10,11]. In the continuation of our chemical studies on this seaweed [10,11,12], we have now isolated four new LDs (**1**–**4**) along with four known linear diterpenes (**5**–**8**), a monoterpene loliolide (**9**), the tetraterpene carotenoid fucoxanthin (**11**) and a truncated fucoxanthin analogue (**10**). The planar structures of the isolated metabolites were elucidated by NMR, HRMS, and FT-IR spectroscopy. Due to low supply of the new compounds, VCD spectroscopy, which requires higher amounts of pure compounds [10,11,12], could not be used for stereochemical assignments. Instead, we employed DFT calculation of NMR chemical shifts, followed by DP4+ analysis, and DFT prediction of optical rotations. This study describes purification, structure determination and cytotoxic activity (in vitro) of the isolated compounds, as well as DFT studies for identification of the configuration of the new compounds **1**–**4**.

## 2. Results and Discussion

**Figure 1:**
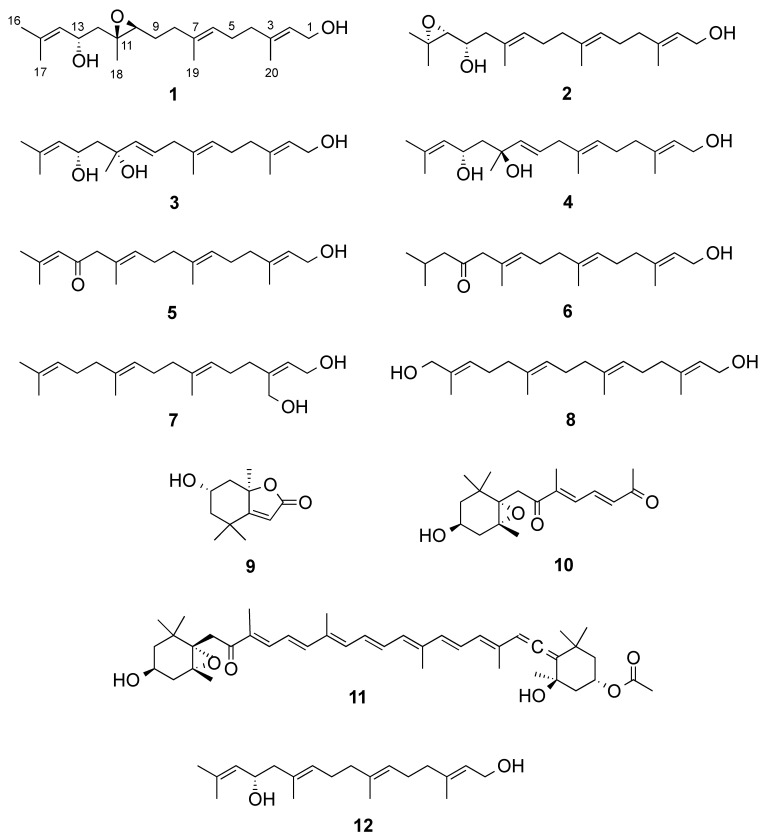
Compounds isolated from *Bifurcaria bifurcata.*

The freeze-dried algal material was successively extracted with CH_2_Cl_2_ and MeOH. The combined organic extracts were submitted to a modified Kupchan partition [10,11]. The CHCl_3_-soluble portion of the crude extract was fractionated by NP-Flash column chromatography (CC) to yield twenty-one fractions (C1−C21). The RP-HPLC separation of fractions C4, C9, C10, and C14 afforded the compounds **1**–**11** (Figure 1).

The new compound **1** was obtained as a colorless film. The molecular formula of **1** was determined by its HR-ESIMS data (*m/z* 345.2401 [M + Na]^+^) as C_20_H_34_O_3_, consistent with four double bond equivalents (DBEs). Analysis of the ^1^H, ^13^C and *g*HSQC NMR spectra indicated the presence of one tertiary methyl δ_H_/δ_C_ 1.30/18.3 (CH_3–_18) and four olefinic methyl groups δ_H_/δ_C_ 1.70/25.7 (CH_3_-16); 1.66/18.2 (CH_3_-17); 1.61/16.0 (H_3_-19) and 1.65/16.2 (H_3_-20), an oxymethylene δ_H_/δ_C_ 4.13/59.4 (CH_2_-1), three olefinic methines δ_H_/δ_C_ 5.38/123.6 (CH-2); δ_H_/δ_C_ 5.16/124.7(CH-6) and 5.15/127.4 (CH-14), two oxymethines δ_H_/δ_C_ 2.98/62.1 (CH-10); and 4.44/65.5 (CH-13) and five methylenes δ_H_/δ_C_ 2.03/39.3 (CH_2_-4); 2.12/26.1 (CH_2_-5); 2.13/36.2 (CH_2_-8); 1.64/26.9 (CH_2_-9) and 1.81,1.74/44.1 (CH_2_-12) (Table 1 and Table 2). The ^13^C NMR spectrum (Table 2) contained four additional fully substituted carbons, including three sp^2^ carbons δ_C_ 134.3 (C-7); 134.7 (C-15); 139.3 (C-3) and one fully substituted oxygenated carbon δ_C_ 60.4 (C-11), which was suggestive of an acyclic diterpene skeleton. A close comparison of the NMR data of **1** with those of eleganediol (**12**, Figure 1), a LD, which we previously isolated from the same seaweed [10] suggested that **1** was a 13-hydroxygeranylgeraniol analogue with an additional oxygenation. The ^1^H NMR signal at δ_H_ 2.98 (t, *J* = 6.2 Hz) and two ^13^C signals at δ_C_ 62.1 (CH) and δ_C_ 60.4 (C), supported the oxygenation to be due to an epoxy functionality in **1**. Three double bonds and an epoxy ring make up for the required four DBEs within **1**.

The planar structure of **1** was assigned by means of 1D and 2D NMR experiments. The *g*COSY spectrum included four spin systems, comprising of (i) H_2_-1 and H-2, (ii) H_2_-4, H_2_-5 and H-6, (iii) H_2_-8, H_2_-9 and the oxirane proton H-10 and (iv) H_2_-12, the oxymethine H-13 and the olefinic proton H-14. This data, especially the spin system iii provided clear indication that the epoxy ring resided between C-10 and C-11. The proton sequences (i–iv) were easily connected to each other with the aid of HMBC correlations. Specifically, diagnostic ^1^H-^13^C long range couplings between H-2/C-1, H_2_-1/C-2, and H_2_-1/C-3 corroborated the position of terminal secondary alcohol at C-1, while HMBC correlations between C-11/H-13, C-13/H_2_-12, C-14/H_2_-12, and C-15/H_2_-12 provided further support for attachment of an OH group at C-13. The final proof for the location of the epoxy ring between C-10 and C-11 came from HMBC correlations observed between H_2_-8/C-10, H_2_-9/C-10 and H-10/C-11, H_2_-9/C-11, H_2_-12/C-11, H-13/C-11, and H_3_-18/C-11.

**Figure 2:**
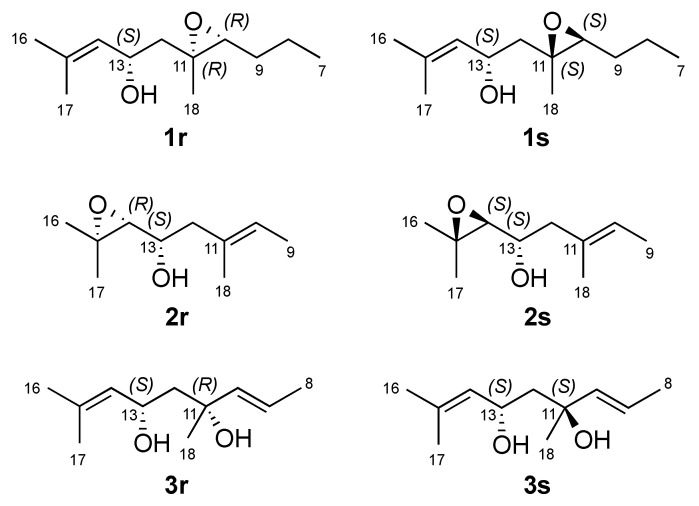
Model compounds used for density functional theory (DFT) calculations of new linear diterpenes **1**–**4**.

**Figure 3:**
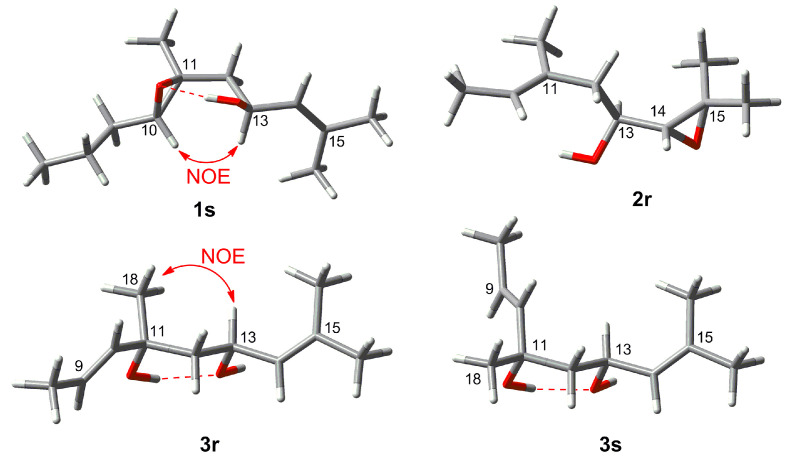
Lowest energy conformations of model compounds **1s**, **2r**, **3r** and **3s**.

The *E* geometry of the double bonds ∆^2,3^, ∆^6,7^ and ∆^14,15^ was deduced on the basis of NOE couplings observed between H-2/H_2_-4, H-6/H_2_-8 and, H-14/H_2_-12 respectively. The olefinic terminal methyl groups H_3_-16 and H_3_-17 were identified as *pro*-*E* and as *pro*-*Z*, respectively, due to further NOE correlations between H-14/H_3_-16 and H-13/H_3_-17. A *trans* stereochemistry for the epoxy ring was suggested by the observed NOESY correlation between H-10 and H_2_-12. Assignment of relative configuration of C-13 and C-10/C-11 was based on DFT prediction of ^1^H and ^13^C chemical shifts [13]. Due to the high conformational flexibility of compound **1**, calculations were performed using the simplified model compounds **1r** (10*R*,11*R*,13*S*) and **1s** (10*S*,11*S*,13*S*), comprising atoms C-7 to C-18 (Figure 2). A systematic conformational search over the six rotable bonds of **1r**/**1s** produced 292 and 251 sterically allowed unique conformers for **1r** and **1s**, respectively. The geometry of all conformers was optimized quantum mechanically using Gaussian 16 [14] with the B3LYP functional, the 6-31G(d,p) basis set, and the SMD continuous solvent model. Conformers with a Boltzmann weight less than 1% were removed, resulting in 15 conformers for **1r** and 16 conformers for **1s**, which were used for subsequent calculations. ^1^H and ^13^C chemical shifts were calculated at the mPW1PW91/6-311+G(2d,p)/SMD level, using the scaling factors determined by the Tantillo group [15], and average chemical shifts were calculated using Boltzmann distribution. Only atoms at positions 10 to 18 of model compounds **1r**/**1s** were considered in the comparison with experimental chemical shift, because they were at least three bonds away from the first point of difference with **1**. The results showed that NMR chemical shifts predicted for model compound **1s** (RMSD of 1.59 ppm for ^13^C and 0.099 ppm for ^1^H) matched experimental chemical shifts much better than those predicted for **1r** (RMSD of 1.96 ppm for ^13^C and 0.206 ppm for ^1^H). DP4+ analysis [16], providing 100% probability of **1s** as the correct stereoisomer, confirmed this assignment. This computational work was supported by the NOESY data. A hydrogen bond between the OH group at C-13 and the oxirane O atom is observed in all the six lowest energy conformations of the **1s**, accounting for 74% of the population. These conformers also show a close proximity (2.13 Å) between H-10 and H-13 (Figure 3), which is in accordance with the intense NOESY correlation peak between these two protons. 

Absolute configuration of compound **1** was determined using DFT prediction of optical rotation (OR) [17,18,19], using the same set of optimized conformers of model compound **1s** (Figure 2) that were used for NMR chemical shift prediction. In doing so, we made the reasonable assumption that the contribution to chiroptical properties, including molar rotation, of a remote non-chiral side chain is negligible [11]. Specific rotations of each conformer were calculated at the B3LYP/TZVP/SMD level, taking care to convert molar rotations into specific rotations using the molecular weight of compound **1** and not that of the model compound **1s**. The calculated Boltzmann-averaged specific rotation was +13.3, in good agreement with the experimental value +17.0. Therefore, compound **1** was identified as 2*E*,6*E*,10*S*,11*S*,13*S*,14*E*)-3,7,11,15-tetramethylhexadeca-2,6,14-triene-10,11-epoxy-1,13-diol, which we named as 10*S*,11*S*-epoxyeleganediol. 

Compound **2** was obtained as a clear oil with a molecular formula of C_20_H_34_O_3_ based on the pseudomolecular ion peak observed at *m/z* 345.2400 [M + Na]^+^ in its HR-ESIMS spectrum. This required four double bond equivalents (DBEs), as in the case of **1**. The inspection of its 1D NMR and HSQC spectra indicated its high similarity to compound **1** (Table 1 and Table 2). The major difference between two compounds was due to different position of the epoxy ring. The appearance of H-14 as a shielded doublet at δ_H_ 2.66 (*J* = 7.7 Hz) and the ^13^C chemical shift values of both C-14 (δ_C_ 67.5, d) and C-15 (δ_C_ 58.3, s) clearly suggested the presence of an oxirane (epoxy) ring at C-14/C-15. Accordingly, the geminal methyl groups H_3_-16 (δ_H_ 1.09) and H_3_-17 (δ_H_ 1.07) were shielded by about 0.6 ppm in comparison to **1**, and H-13 was shielded to δ_H_ 3.51. The observed ^1^H-^1^H COSY correlations between two oxymethine protons H-13 and H-14, as well as the HMBC correlations between H-14/C-13, H-12/C-13, H-13/C-14, H-14/C-12, H_3_-16/C-14, H_3_-16/C-15, H_3_-17/C-14 and H_3_-17/C-15 completed the planar structure of **2** as 14,15-epoxyeleganediol. Configuration of the two stereocenters in compound **2** was determined by DFT prediction of ^1^H and ^13^C chemical shifts and specific rotation. Model compounds **2r** (13*S*,14*R*) and **2s** (13*S*,14*S*) were used for calculations (Figure 2). Systematic conformational search generated 61 and 64 unique conformers for **2r** and **2s**, respectively. Optimization of the conformers and chemical shift prediction using the same level of theory used for compound **1** provided unsatisfactory results, because predicted ^1^H and ^13^C chemical shifts led to opposite conclusions. Because a most critical part in DFT studies of flexible molecules is the correct evaluation of the conformational ensemble, optimization and evaluation of the energy of conformers was repeated at a higher level of theory, namely B3LYP/TZVP/SMD. This led to 12 low-energy conformers (Boltzmann weight > 1%) for **2r** and 15 low-energy conformers for **2s**, which were used for NMR chemical shift prediction as described above. Model compound **2r** matched experimental chemical shifts much better (RMSD of 1.63 ppm for ^13^C and 0.131 ppm for ^1^H, positions 11 to 18 were considered in the comparison) than model compound **2s** (RMSD of 2.15 ppm for ^13^C and 0.178 ppm for ^1^H), and DP4+ analysis confirmed this observation with 99.98% probability for **2r**. Absolute configuration of compound **2** was determined by OR prediction using the same methods described above for **1**. The predicted [α]_D_ –5.8 agreed in sign with the experimental [α]_D_ –19.5. Therefore, compound **2** was identified as (2*E*,6*E*,10*E*,13*S*,14*R*)-3,7,11,15-tetramethylhexadeca-2,6,10-triene-14,15-epoxy-1,13-diol, which we named as 14*R*,15-epoxyeleganediol.

Compound **3** was isolated as a colorless oil. The same molecular formula C_20_H_34_O_3_ was assigned to **3** based on HR-ESIMS data *m/z* 345.2398 [M + Na]^+^ and ^13^C NMR data (Table 2), requiring four DBEs. Based on its 1D NMR data, **3** shared many common features with **1** and **2** and was readily identified as another 13-hydroxygeranylgeraniol derivative (Table 1 and Table 2). The two most striking differences of **3** to **1** and **2** were ascribed to (i) the presence of a fourth double bond that emerged as an AB system in the ^1^H NMR spectrum at δ_H_ 5.60 (dt, *J* = 15.6, 6.4 Hz, H-9) and δ_H_ 5.54 (br. d, *J* =15.6 Hz, H-10) (Table 1) and (ii) the replacement of the epoxy signals with an sp^2^ quaternary carbon (δ_C_ 73.0, s), indicating the presence of a tertiary OH group in the middle chain. The latter (OH group) was assigned to C-11, which was supported by diagnostic HMBC correlations from H-9, H-10, H_2_-12, H-13, and CH_3_-18 to C-11. The position of the olefinic bond was deduced to be at ∆^9,10^ based on the COSY cross peaks (between H-9/H-10 and H-9/H_2_-8) and key HMBC correlations between H-8/C-9, H_2_-8/C-10, H-9/C-8, H-9/C-10, H-10/C-8, H-10/C-9, and H_3_-18/C-9 and H_3_-18/C-10. The *E* geometry of ∆^9,10^ was evident from the coupling constant (*J*_9,10_ = 15.6 Hz). The NOESY spectrum of **3** also supported the all *E* geometry of the remaining double bonds at C-2, C-6 and C-14. 

Configuration at C-11 and C-13 was determined on the basis of DFT studies, using the two diastereomeric model compounds **3r** and **3s**. Systematic conformational search defined 240 conformers for **3r** and 217 conformers for **3s** (Figure 2). After DFT optimization at the B3LYP/6-31G(d,p)/SMD level, 15 low energy conformers (Boltzmann weight > 1%) for **3r** and 6 low energy conformers for **3s** were used for chemical shift prediction, which however was not conclusive in this case. Indeed, fitting with experiment of chemical shifts predicted for **3r** for ^1^H was good (RMSD of 1.59 ppm for ^13^C and 0.131 ppm, positions 10 to 18 were considered in the comparison) but that for **3s** was not much worse (RMSD of 1.66 ppm for ^13^C and 0.157 ppm for ^1^H), while DP4+ analysis showed only a moderate preference (91.49%) toward **3r**. However, DFT calculations also showed for both model compounds a strong preference (>90% of population) for conformations with an intramolecular hydrogen bond between the two OH groups, resulting in a chair-like six-membered ring (Figure 3). In this frame, the prominent NOESY correlation peak between H_3_-18 and H-13 could be interpreted as an indication of the axial-like orientation of the methyl group, and therefore of the 11R*,13S* relative configuration as in model compound **3r**. Absolute configuration of compound **3** could not be determined using OR prediction as for compounds **1** and **2**, because the measured [α]_D_ +5.1 was close to 0, making any configurational assignment unreliable [20]. However, considering that eleganediol (**12**) and all LDs we have isolated so far from the Irish *B. bifurcata* show the 13*S* configuration [10,11]*,* we suggest the same 13*S* configuration for compound **3** on biosynthetic grounds. Thus, the proposed absolute stereostructure of compound **3** is (2*E*,6*E*,9*E*,11*R*,13*S*,14*E*)-3,7,11,15-tetramethylhexadeca-2,6,9,14-tetraene-1,11,13-triol, i.e., 11*R*-hydroxyeleganediol. 

Based on the HR-ESIMS data (*m/z* 323.2581 [M + H]^+^), compound **4** was assigned the same molecular formula as **3**, C_20_H_34_O_3_. Also the ^1^H and ^13^C NMR data of **4** were very similar to those of **3** (Table 1 and Table 2). The only significant difference observed between the two compounds was the downfield shift of the CH_3_-18 methyl carbon from δ_C_ 27.0 to δ_C_ 31.0 in **4** (Table 2). The planar structure of compound **4** was the same as for **3**, as shown with the aid of a full set of 2D NMR experiments, *g*COSY, *g*HSQC, *g*HMBC, and the configuration of the three double bonds was also the same. The compound was therefore either one of the two possible (and enantiomeric to each other) 11*S*,13*S* or 11*R*,13*R* diastereomers of **3**. This assignment was supported by the DFT study performed for **3** (in which the model compound **3s** has the 11*S*,13*S* configuration), combined with the NOESY data. Firstly, DP4+ analysis using the chemical shifts computed for **3r**/**3s** showed a 100.00% probability for **3s**. In addition, the prominent correlation peak between H_3_-18 and H-13 observed in the NOESY spectrum of compound **3** was completely absent in the NOESY spectrum of **4**, because the methyl group is in the equatorial-like position in model compound **3s** (Figure 3), and thus in **4**. The OR of compound **4** was still smaller than that of compound **3**, and its absolute configuration could only be proposed on biogenetic grounds assuming the 13*S* configuration universally observed for LDs from *B. bifurcata*. Thus, the proposed absolute stereostructure of **4** is (2*E*,6*E*,9*E*,11*S*,13*S*)-3,7,11,15-tetramethylhexadeca-2,6,9,14-tetraene-1,11,13-triol, i.e., 11*S*-hydroxyeleganediol. 

In addition, seven known compounds (**5**–**11**) were isolated. Based on their 1D/2D NMR, HR-ESIMS, and [α]_D_ data, they were identified as eleganolone (**5**) [6],^6^ its dehidyro-derivative (**6**) [6,21], 20-hydroxygeranylgeraniol (**7**) [22,23], 16-hydroxygeranylgeraniol (**8**) [21,24], loliolide (**9**) [25], a truncated fucoxanthin analogue (**10**) [26], and fucoxanthin (**11**) [27]. 

All compounds isolated in this study were tested for their growth inhibitory potential activity against the human breast cancer cell line MDA-MB-231. The known compounds **5**, **6**, **7**, and **8** displayed moderate cytotoxicity (IC_50_ values 13.0, 33.5, 10.0, and 14.5 μg/mL, respectively). Compound **2** inhibited the cancer cell growth (78.8%) at 100 μg/mL test concentration but an IC_50_ value could not be determined due to minor amounts available. The remaining compounds were devoid of cytotoxicity (IC_50_ > 100 μg/mL).

Our studies on the Irish *B. bifurcata* so far have pointed out a high structural diversity of LDs [10,11,12]. Interestingly, the majority of the LDs (**1**–**6**) are 13-hydroxy or 13-ketogeranylgeraniol derivatives, while **7**–**8** are diols containing the second OH function on one of the methyl groups. Notably, C-13-hydroxy substituted new LDs (**1**–**4**) have a C13-*S* configuration, and bear additional oxidations on the geranylgeraniol backbone that generates further stereocenters. The flexible nature of LDs, however, often prevents clear stereochemical assignments of these stereocenters by conventional methods, such as NOESY NMR. The present study shows that DFT studies can assist with interpretation of NMR data for such molecules, provided that proper care is given to conformational search and evaluation of the populations of conformers. Even when chemical shift prediction does not provide conclusive results, as it happened for compound **3**, the knowledge about the conformational behavior of the molecule acquired through DFT studies provides a reliable way to turn NOESY or spin-spin coupling data into configurational assignments. Previously, we applied IR/VCD spectroscopy coupled with DFT calculations to identify the absolute configuration of LDs from *B. bifurcata* [10,11]. This has proved to be a valuable method, but due to its low sensitivity, VCD analyses require relatively large amounts of compound (5–10 mg) [10,11,12]. ECD has been used to determine absolute configuration of few LDs with stronger UV chromophores [28]. In the present study, absolute configuration of LDs has been determined using OR prediction. This method is critically dependent on the quality of the underlying conformational search, and cannot be used when the experimental specific rotation is close to zero [20]. However, when applicable it allows to access absolute configuration of compounds devoid of chromophores and only available in sub-milligram amounts, without the need for special equipment.

Various classes of algal terpenoids exert diverse biological activities, such as antimicrobial or anticancer [29,30,31], thereby holding promising potential in marine biodiscovery. However, the real potential of algal terpenes in drug discovery remains relatively untapped. Linear diterpenes obtained from brown algae, specifically from *Bifurcaria bifurcata* have been reported to exhibit cytotoxicity [9] and growth inhibitory activity against cancer cell lines [12,32]. These activities are generally moderate but medicinal chemistry or formulation studies may improve their potency. 

## 3. Materials and Methods 

### 3.1. General Experimental Procedures 

Specific rotations of the metabolites were measured on a Unipol L1000 Schmidt+Haensch polarimeter at the sodium D line (589.3 nm, 20 °C) using a 10 cm cell. UV spectra were obtained in spectroscopic grade CHCl_3_ or MeOH on a Varian, Cary 100 UV-Vis spectrophotometer. FT-IR spectra were recorded on a Perkin Elmer 400 or a Perkin Elmer Spectrum One ATR FT-IR spectrometer. NMR spectra were acquired on a Varian 500 MHz or an Agilent 600 MHz spectrometer. Chemical shifts are expressed as δ (ppm) referenced to the residual solvent signal (CDCl_3_: δ_H_ 7.24, δ_C_ 77.0 or C_6_D_6_: δ_H_ 7.16, δ_C_ 128.0), and *J* values are in Hz. HPLC separations were achieved on an Agilent 1260 system equipped with a diode array and an ELSD detector. A Kromasil 100 C18 5u (250 × 8 mm, 5 μm) RP-HPLC column was employed for HPLC studies. HRMS data were measured on an Agilent QTOF 6540 MS system (ESI, positive ion mode), coupled to an Agilent 1290 Infinity UPLC system, operating the elution gradient: 50% B for 8 min, increasing to 100% B in 3 min, maintaining 100% B for 5 min (solvent A: H_2_O + 0.1% formic acid, solvent B: MeCN + 0.1% formic acid), on a Zorbax Eclipse Plus C18 RRHD (50 × 2.1 mm, 1.8 μm) column, at 0.5 mL/min, with UV detection at 200−600 nm. Thin layer chromatography (TLC) analyses were performed on Kieselgel 60 F254 aluminum support plates (Merck) and spots were visualized by vanillin/H_2_SO_4_ reagent (6% vanillin and 15% H_2_SO_4_ in MeOH). All solvents were of HPLC or LCMS grade and were purchased from Sigma Aldrich.

### 3.2. Algal Material 

Details of the collection site and taxonomic identification of the algal sample have previously been reported [10,11]. *Bifurcaria bifurcata* was collected from the intertidal rock pool at Kilkee, Co. Clare of Ireland, in May 2009. A voucher specimen is retained at the Marine Biodiscovery Laboratory of the Irish Marine Institute (code number BDV0015).

### 3.3. Extraction and Isolation

The freeze-dried algal biomass (132.4 g dry weight) was extracted with CH_2_Cl_2_ and MeOH. The organic extracts were combined and evaporated to dryness on a rotary evaporator. The resulting dark green residue (12.0 g) was submitted to a modified Kupchan partition where the crude extract was dissolved in 90%MeOH (200 mL) and partitioned against *n*-hexane (3 × 200 mL). The water concentration was increased to 35%, before partitioning against CHCl_3_. Evaporation of the solvents under vacuum afforded the CHCl_3_ subextract (7.6 g). 

The CHCl_3_ subextract (6.8 g) was fractionated by a Flash CC system (Agilent 971FP, pre-packed silica column SF25–80g), operating with the following gradient: 0% B for 5 min, to 5% B in 15 min, at 5% B for 10 min, to 10% B in 10 min, at 10% B for 10 min, to 40% B in 40 min, to 100% B in 10 min, at 100% B for 10 min, solvent A: *n*-hexanes, solvent B: EtOAc, flow of 25 mL/min afforded 21 fractions (C1-C21). Fraction C4 (43.9 mg) was subjected to RP-HPLC. The gradient elution using 55% B for 13 min, increasing to 100% B in 5 min, maintaining 100% B for 20 min (solvent A: H_2_O, solvent B: MeCN), at a flow of 1.5 mL/min, afforded **5** (3.2 mg, t_R_ 32.2 min) and **6** (1.3 mg, t_R_ 33.7 min). Fraction C9 (105.5 mg) was subjected to RP-HPLC on the same system, and under the aforementioned conditions, to give pure **9** (1.6 mg, t_R_ 10.7 min), **10** (0.9 mg, t_R_ 11.8 min), **1** (1.2 mg, t_R_ 23.7 min), **8** (1.1 mg, t_R_ 31.5 min), and **11** (21.0 mg, t_R_ 60.6 min). Fraction C10 (118.0 mg) was subjected to RP-HPLC on the same system using the same gradient elution to afford **3** (3.5 mg, t_R_ 27.4 min), **2** (1.0 mg, t_R_ 27.9 min) and **4** (1.5 mg, t_R_ 28.8 min). Compound **7** (1.9 mg, t_R_ 34.2 min) was isolated from fraction C14 (19.5 mg) by RP-HPLC using 100% MeCN as the eluent. 

### 3.4. Computational Studies

Systematic conformational search for model compounds **1r**, **1s**, **2r**, **2s**, **3r**, **3s** was performed using the Search_Compare module within the Insight II/Discover package. After being generated, each conformer was optimized in the CFF91force field, and duplicate conformers were removed. The dihedral angles involved in the search were those about the bonds C-8/C-9, C-9/C-10, C-11/C-12, C-12/C-13, C-13/C-14, and C-13/OH for **1r** and **1s**, those about the bonds C-11/C-12, C-12/C-13, C-13/C-14, and C-13/OH for **2r** and **2s**, and those about the bonds C-9/C-10, C-10/C-11, C-11/C-12, C-13/C-14, C-11/OH and C13/OH for **3r** and **3s**. Each dihedral angle was scanned in steps of 60°. 

Conformers generated by the systematic search were optimized using density functional theory (DFT) with the Gaussian 16 program [14], the B3LYP functional, the 6–31G(d,p) basis set (**1r**, **1s**, **3r**, and **3s**) or the TZVP basis set (**2r** and **2s**), and the SMD continuous solvent model for CHCl_3_. Only significantly populated conformers (population > 1% at 298 K according to the Boltzmann statistics based on internal energy) were used for the subsequent calculation steps. No imaginary frequencies were found by vibrational frequency analysis, showing that all conformers of all model compounds were in a true energy minimum. Cartesian coordinates of these conformers can be found in Tables S1–S6. 

NMR chemical shifts were calculated from isotropic shieldings calculated at the mPW1PW91/6-311+G(2d,p)/SMD(CHCl_3_) level of theory, using the scaling factors determined by Lodewyk et al. [15]. for this level of theory (slope: −1.0936, intercept: 31.8018 for ^1^H and slope: −1.0533, intercept: 186.5242 for ^13^C); the results are reported in Tables S7–S12. Optical rotations were calculated using time-dependent DFT (TDDFT) at the B3LYP/TZVP/SMD(CHCl_3_) level; the results are reported in Table S13. Average chemical shifts and specific rotations were obtained using Boltzmann statistics. 

*10S,11S-Epoxyeleganediol (***1***)*: Colorless oil; [α]_D_ +17.0 (*c* 0.20, CHCl_3_); UV (CHCl_3_) *λ*_max_ (log *ε*) 233 (2.11) nm; IR (film) *ν*_max_ 3408, 2942, 2861, 1381, 1170, 1024 cm^−1^; ^1^H NMR (600 MHz, CDCl_3_) and ^13^C NMR (150 MHz, CDCl_3_) see Table 1 and Table 2; HRESIMS *m/z* 345.2401 [M + Na]^+^ (calcd for C_20_H_34_O_3_Na, 345.2400). 

*14R,15-Epoxyeleganediol (***2***)*: Colorless oil; [α]_D_ −19.5 (*c* 0.13, MeOH); UV (CHCl_3_) *λ*_max_ (log *ε*) 239 (2.03) nm; IR (film) *ν*_max_ 3379, 2925, 1683, 1619, 1444, 1381, 1009 cm^−1^; ^1^H NMR (600 MHz, C_6_D_6_) and ^13^C NMR (150 MHz, C_6_D_6_) see Table 1 and Table 2; HRESIMS *m/z* 345.2400 [M + Na]^+^ (calcd for C_20_H_34_O_3_Na, 345.2400).

*11R-Hydroxyeleganediol(***3***)*: Colorless oil; [α]_D_ +5.1 (*c* 0.23, MeOH); UV (CHCl_3_) *λ*_max_ (log *ε*) 236 (2.01) nm; IR (film) *ν*_max_ 3354, 2925, 1669, 1441, 1376, 1242, 1032, 974, 856 cm^−1^; ^1^H NMR (500 MHz, CDCl_3_) and ^13^C NMR (125 MHz, CDCl_3_) see Table 1 and Table 2; HRESIMS *m/z* 345.2398 [M + Na]^+^ (calcd for C_20_H_34_O_3_Na, 345.2400) and *m/z* 667.4906 [2M + Na]^+^ (calcd for C_40_H_68_O_6_Na, 667.4908).

*11S-Hydroxyeleganediol(***4***)*: Colorless oil; [α]_D_ −2.0 (*c* 0.10, MeOH); UV (CHCl_3_) *λ*_max_ (log *ε*) 235 (1.99) nm; IR (film) *ν*_max_ 3380, 2925, 1668, 1455, 1379, 1246, 1033, 918 cm^−1^; ^1^H NMR (600 MHz, C_6_D_6_; 500 MHz, CDCl_3_) and ^13^C NMR (150 MHz, C_6_D_6_) see Table 1 and Table 2; HRESIMS *m/z* 323.2581 [M + H]^+^ (calcd for C_20_H_35_O_3_, 323.2581).

### 3.5. Anticancer Activity Assessments 

The breast cancer cell line MDA-MB-231 (ATCC) was used for bioassays. The cell line was maintained in Dulbecco’s Modified Eagle Medium (DMEM, Sigma-Aldrich) supplemented with 10% fetal bovine serum and antibiotics (1% penicillin/streptomycin, Sigma-Aldrich) and incubated at 37 °C at 5% CO_2_. The cells were seeded in a 96 well plate (1 × 10^4^ cells per well) followed by culturing for 24 h (37 °C, 5% CO_2_) before being treated with test samples at a final concentration of 0–100 μg/mL. The vehicle control used was 1% DMSO, while 10 μM 5-Fluorouracil served as positive control. Cell viability was assessed after 72 h by Alamar Blue assay. For this aim, 40 μL Alamar Blue (0.56 mM) was added to each well containing 200 μL of cell culture medium (93 μM final Alamar Blue concentration). After incubation for 6 h, the fluorescence of each well was assessed (λ_ex_ = 530 nm; λ_em_ = 595 nm) using Victor 3V 1420 multilabel counter. Cell viability was calculated and expressed as a percentage of untreated control cells. The data are the mean ± SD of three experiments (in triplicates) and GraphPad Prism software was used to plot the data and to determine the IC_50_ values. 

## Figures and Tables

**Table 1:** ^1^H NMR data (600 MHz, CDCl_3_) of **1**–**4**.

C	1	2 ^a^	3 ^b^	4 ^a^	4 ^b^
	δ_H_ (*J* in Hz)	δ_H_ (*J* in Hz)	δ_H_ (*J* in Hz)	δ_H_ (*J* in Hz)	δ_H_ (*J* in Hz)
1	4.13, br. d (6.9)	4.00, d (6.7)	4.13, br. d (6.8)	3.97, d (6.6)	4.13, br. d (6.8)
2	5.38, br. t (6.9)	5.43, t (6.7)	5.37, br. t (6.8)	5.39, br. t (6.6)	5.37, br. t (6.8)
3	-	-	-	-	-
4	2.03, br. t (7.4)	2.02, m	2.03, m	2.00, m	2.03, m
5	2.12, m	2.12, m	2.10, m	2.13, m	2.12, m
6	5.16, m	5.22, t (6.9)	5.10, br. t (6.9)	5.28, br. t (7.0)	5.15, br. t (7.1)
7	-	-	-	-	-
8	2.13, m	2.03, m	2.65, br. d (6.4)	2.75, br. d (7.0)	2.71 br. d (6.9)
9	1.64, m	2.13, m	5.60, dt (15.6, 6.4)	5.95, dt (15.3, 7.0)	5.7, dt (15.4, 6.9)
10	2.98, t (6.2)	5.23, t (5.8)	5.54, br. d (15.6)	5.47, br. d (15.3)	5.52, br. d (15.4)
11	-	-	-	-	-
12	1.81, dd (14.6, 3.4) 1.74, dd (14.6, 9.1)	2.23, dd (13.3, 7.7) 2.10 m	1.78, dd (14.6, 10.0) 1.52, dd (14.6, 2.9)	1.83, dd (14.4, 10.8) 1.39, dd (14.4, 1.9)	1.79, dd (14.5, 10.5) 1.48, dd (14.5, 1.8)
13	4.44 td (9.1, 3.4)	3.51, ddd (7.7, 7.7, 5.6)	4.74, ddd (10.0, 8.6, 2.9)	4.70, ddd (10.8, 8.5, 1.9)	4.62 ddd (10.5, 8.6, 1.8)
14	5.15, m	2.66, d (7.7)	5.19, dhept (8.6, 1.2)	5.25, dhept (8.5, 1.2)	5.18, dhept (8.6, 1.2)
15	-	-	-	-	-
16	1.70, br. s	1.09, s	1.69, d (1.2)	1.56, d (1.2)	1.68, d (1.2)
17	1.66, br. s	1.07, s	1.67, d (1.2)	1.52, d (1.2)	1.61, d (1.2)
18	1.30, s	1.52, br. s	1.37, s	1.26, s	1.25, s
19	1.61, br. s	1.56, br. s	1.56, br. s	1.60, br. s	1.59, br. s
20	1.65, br. s	1.48, br. s	1.65, br. s	1.45, br. s	1.55, br. s

^a^ Acquired in C_6_D_6_, ^b^ Acquired at 500 MHz.

**Table 2:** ^13^C NMR data (150 MHz, CDCl_3_) of **1**–**4**.

C	1	2 ^a^	3 ^b^	4 ^a^
	δ_C_, type	δ_C_, type	δ_C_, type	δ_C_, type
1	59.4, CH_2_	59.3, CH_2_	59.4, CH_2_	59.5, CH_2_
2	123.6, CH	125.1, CH	123.9, CH	125.4, CH
3	139.3, C	137.8, C	139.0, C	137.3, C
4	39.3, CH_2_	39.7, CH_2_	39.2, CH_2_	39.5, CH_2_
5	26.1, CH_2_	26.5, CH_2_	25.9, CH_2_	26.2, CH_2_
6	124.7, CH	124.8, CH	124.8, CH	124.8, CH
7	134.3, C	134.9, C	134.0, C	134.6, C
8	36.2, CH_2_	39.8, CH_2_	42.2, CH_2_	42.8, CH_2_
9	26.9, CH_2_	26.7, CH_2_	125.7, CH	126.9, CH
10	62.1, CH	128.1, CH	138.9, CH	138.2, CH
11	60.4, C	131.1, C	73.0, C	73.6, C
12	44.1, CH_2_	44.8, CH_2_	47.6, CH_2_	47.8, CH_2_
13	65.5, CH	69.2, CH	66.3, CH	67.5, CH
14	127.4, CH	67.5, CH	127.8, CH	129.5, CH
15	134.7, C	58.3, C	134.6, C	132.7, C
16	25.7, CH_3_	24.9, CH_3_	25.7, CH_3_	25.6, CH_3_
17	18.2, CH_3_	19.4, CH_3_	18.2, CH_3_	18.1, CH_3_
18	18.3, CH_3_	16.6, CH_3_	27.0, CH_3_	31.0, CH_3_
19	16.0, CH_3_	16.0, CH_3_	16.2, CH_3_	16.7, CH_3_
20	16.2, CH_3_	16.1, CH_3_	16.1, CH_3_	16.2, CH_3_

^a^ Acquired in C_6_D_6_, ^b^ Acquired at 125 MHz.

## Data Availability

Data can be requested from the corresponding author.

## Supplementary Materials

The following are available online at https://www.mdpi.com/1660-3397/19/1/42/s1. Supplementary Figures S1–S33: NMR, HRESIMS and FT-IR spectra of compounds **1**–**4**. Supplementary Tables S1–S13: Detailed information of computational studies with model compounds.
