# Supplementary material for: Density Functional Theory (DFT)-Aided Structure Elucidation of Linear Diterpenes from the Irish Brown Seaweed Bifurcaria bifurcata
Source: Mar Drugs. 2021 Jan 19;19(1):42. doi: 10.3390/md19010042 (PMC7832306; doi:10.3390/md19010042)
Supplement: Supplementary file 1 [file marinedrugs-19-00042-s001.pdf]

Supplementary Information for

Density Functional Theory (DFT)-Aided Structure Elucidation of Linear Diterpenes from the Irish Brown Seaweed *Bifurcaria bifurcata*

Vangelis Smyrniotopoulos [1], Daria Firsova [1,], Howard Fearnhead [2], Laura Grauso, [3] Alfonso Mangoni, [4] and Deniz Tasdemir [1,5,6,*]

1 School of Chemistry, National University of Ireland Galway, University Road, Galway, Ireland; vsmy@hotmail.com; daria.firsova@abbvie.com
2 Pharmacology and Therapeutics, School of Medicine, National University of Ireland Galway, University Road, Galway, Ireland; howard.fearnhead@nuigalway.ie, ORCID ID: 0000-0002-8054-9794
3 Dipartimento di Agraria, Università degli Studi di Napoli Federico II, 80055 Portici (NA), Italy; laura.grauso@unina.it, ORCID ID: 0000-0001-9165-8796
4 Dipartimento di Farmacia, Università degli Studi di Napoli Federico II, 80131 Napoli, Italy; alfonso.mangoni@unina.it, ORCID ID: 0000-0003-3910-6518
5 GEOMAR Centre for Marine Biotechnology (GEOMAR-Biotech), Research Unit Marine Natural Product Chemistry, GEOMAR Helmholtz Centre for Ocean Research Kiel, Am Kiel-Kanal 44, 24106 Kiel, Germany
6 Faculty of Mathematics and Natural Sciences, Kiel University, Christian-Albrechts-Platz 4, 24118 Kiel, Germany

* Correspondence: dtasdemir@geomar.de; Tel.: +49-431-600-4430, ORCID ID: 0000-0002-7841-6271

List of Figures

Figure S1. ^{1}H NMR spectrum of compound **1** (600 MHz, $CDCl_3$)

Figure S2. ^{13}C NMR spectrum of compound **1** (150 MHz, $CDCl_3$)

Figure S3. *g*HSQC spectrum of compound **1** (600/150 MHz, $CDCl_3$).

Figure S4. *g*COSY spectrum of compound **1** (600 MHz, $CDCl_3$).

Figure S5. *g*HMBC spectrum of compound **1** (600/150 MHz, $CDCl_3$).

Figure S6. NOESY spectrum of compound **1** (600 MHz, $CDCl_3$).

Figure S7. HR-ESIMS report of compound **1**.

Figure S8. FT-IR spectrum of compound **1**.

Figure S9. ^{1}H NMR spectrum of compound **2** (600 MHz, CD_6D_6).

Figure S10. ^{13}C NMR spectrum of compound **2** (150 MHz, CD_6D_6).

Figure S11. *g*HSQC spectrum of compound **2** (600/150 MHz, CD_6D_6).

Figure S12. *g*COSY spectrum of compound **2** (600 MHz, CD_6D_6).

Figure S13. *g*HMBC spectrum of compound **2** (600/150 MHz, CD_6D_6).

Figure S14. NOESY spectrum of compound **2** (600 MHz, CD_6D_6).

Figure S15. HR-ESIMS report of compound **2**.

Figure S16. FT-IR spectrum of compound **2**.

Figure S17. ^{1}H NMR spectrum of compound **3** (500 MHz, $CDCl_3$).

Figure S18. ^{13}C NMR spectrum of compound **3** (125 MHz, $CDCl_3$).

Figure S19. *g*HSQC spectrum of compound **3** (500/125 MHz, $CDCl_3$).

Figure S20. *g*COSY spectrum of compound **3** (500 MHz, $CDCl_3$).

Figure S21. *g*HMBC spectrum of compound **3** (500/125 MHz, $CDCl_3$).

Figure S22. NOESY spectrum of compound **3** (500 MHz, $CDCl_3$).

Figure S23. HR-ESIMS report of compound **3**.

Figure S24. FT-IR spectrum of compound **3**.

Figure S25. ^{1}H NMR spectrum of compound **4** (500 MHz, $CDCl_3$).

Figure S26. *g*COSY spectrum of compound **4** (500 MHz, $CDCl_3$).

Figure S27. *g*HSQC spectrum of compound **4** (500/125 MHz, $CDCl_3$).

Figure S28. ^{1}H NMR spectrum of compound **4** (600 MHz, CD_6D_6).

Figure S29. ^{13}C NMR spectrum of compound **4** (150 MHz, CD_6D_6).

Figure S30. *g*HMBC spectrum of compound **4** (600/150 MHz, CD_6D_6).

Figure S31. NOESY spectrum of compound **4** (600 MHz, CD_6D_6).

Figure S32. HR-ESIMS report of compound **4**.

Figure S33. FT-IR spectrum of compound **4**.

<u>**List of Tables:**</u>

Table S1. Cartesian coordinates, energies, and population at 298 K of significantly populated conformers of model compound **1r**.

Table S2. Cartesian coordinates, energies, and population at 298 K of significantly populated conformers of model compound **1s**.

Table S3. Cartesian coordinates, energies, and population at 298 K of significantly populated conformers of model compound **2r**.

Table S4. Cartesian coordinates, energies, and population at 298 K of significantly populated conformers of model compound **2s**.

Table S5. Cartesian coordinates, energies, and population at 298 K of significantly populated conformers of model compound **3r**.

Table S6. Cartesian coordinates, energies, and population at 298 K of significantly populated conformers of model compound **3s**.

Table S7. Isotropic shieldings of significantly populated conformers of model compound **1r**, average isotropic shieldings over conformers, linearly scaled calculated chemical shifts, experimental chemical shifts of compound **1**, and errors.

Table S8. Isotropic shieldings of significantly populated conformers of model compound **1s**, average isotropic shieldings over conformers, linearly scaled calculated chemical shifts, experimental chemical shifts of compound **1**, and errors.

Table S9. Isotropic shieldings of significantly populated conformers of model compound **2r**, average isotropic shieldings over conformers, linearly scaled calculated chemical shifts, experimental chemical shifts of compound **2**, and errors.

Table S10. Isotropic shieldings of significantly populated conformers of model compound **2s**, average isotropic shieldings over conformers, linearly scaled calculated chemical shifts, experimental chemical shifts of compound **2**, and errors.

Table S11. Isotropic shieldings of significantly populated conformers of model compound **3r**, average isotropic shieldings over conformers, linearly scaled calculated chemical shifts, experimental chemical shifts of compound **3**, and errors.

Table S12. Isotropic shieldings of significantly populated conformers of model compound **3s**, average isotropic shieldings over conformers, linearly scaled calculated chemical shifts, experimental chemical shifts of compound **4**, and errors.

Table S13. Calculated relative energies (ΔE), populations, and optical rotations of significantly populated conformers of model compounds **1s** and **2r** at the B3LYP/TZVP/SMD(CHCl3) level.

Figure S1. ^{1}H NMR spectrum of compound **1** (600 MHz, $CDCl_3$)

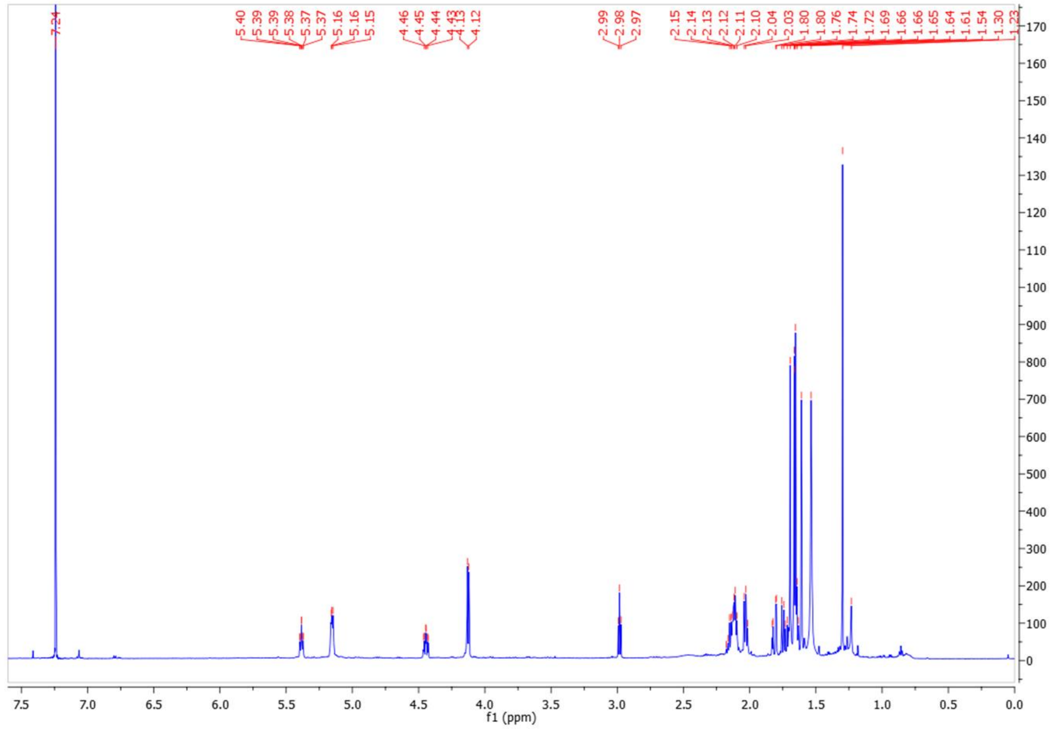

Figure S2. ^{13}C NMR spectrum of compound **1** (150 MHz, $CDCl_3$)

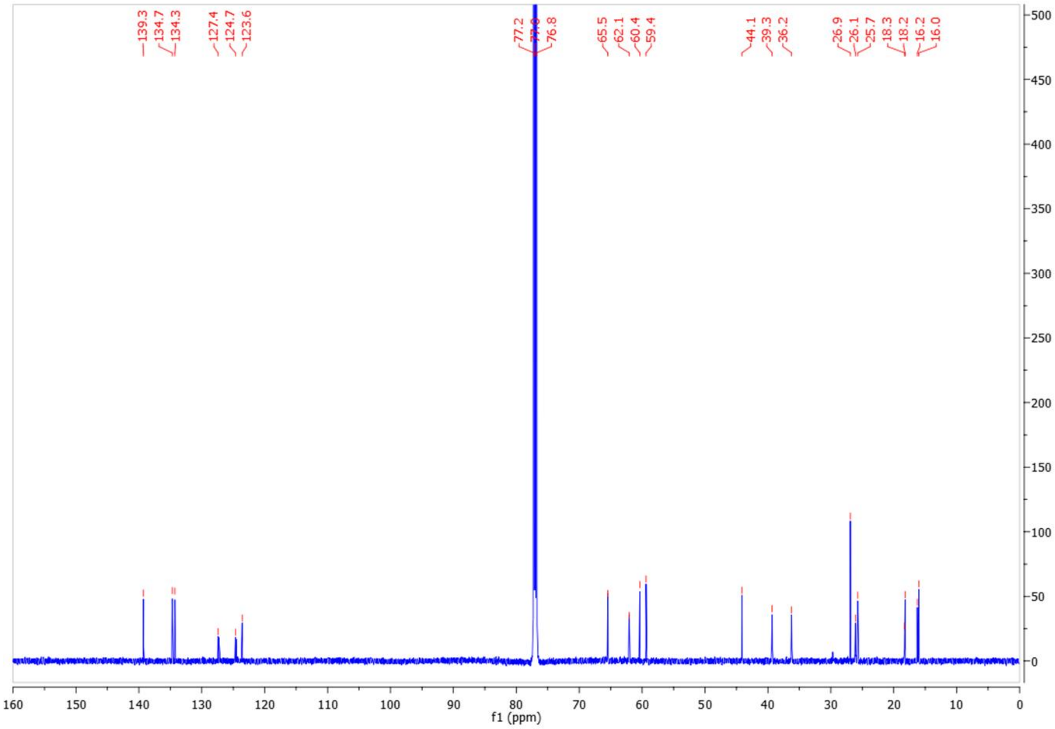

Figure S3. *g*HSQC spectrum of compound **1** (600/150 MHz, $CDCl_3$)

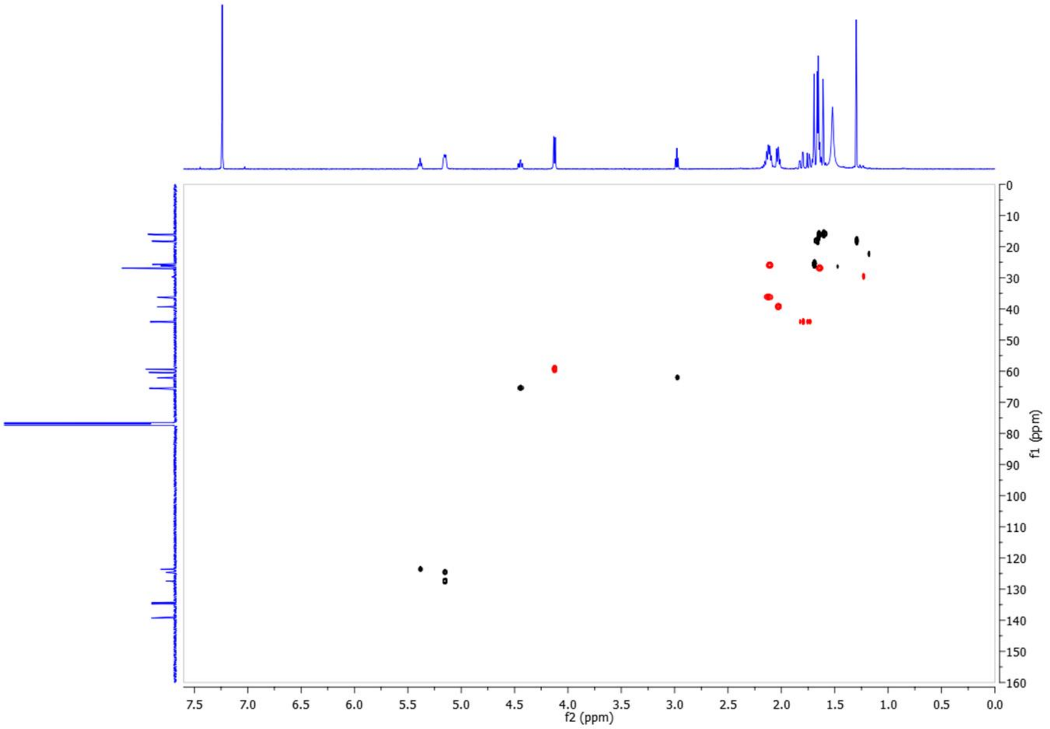

Figure S4. *g*COSY spectrum of compound **1** (600 MHz, $CDCl_3$)

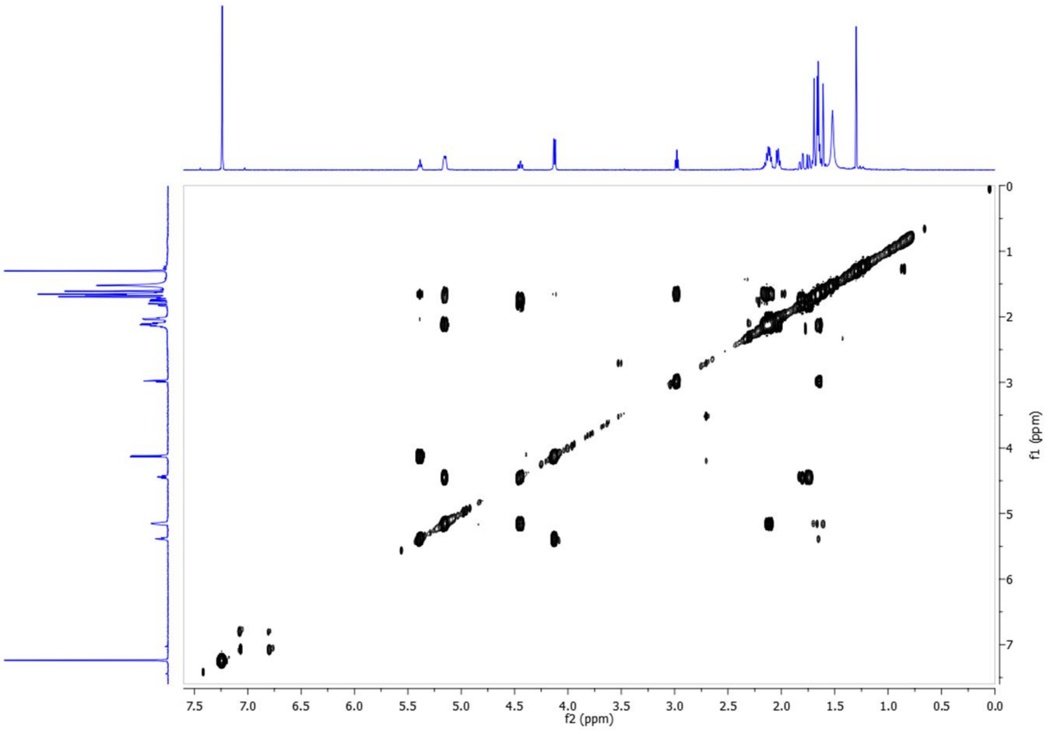

Figure S5. *g*HMBC spectrum of compound **1** (600/150 MHz, $CDCl_3$).

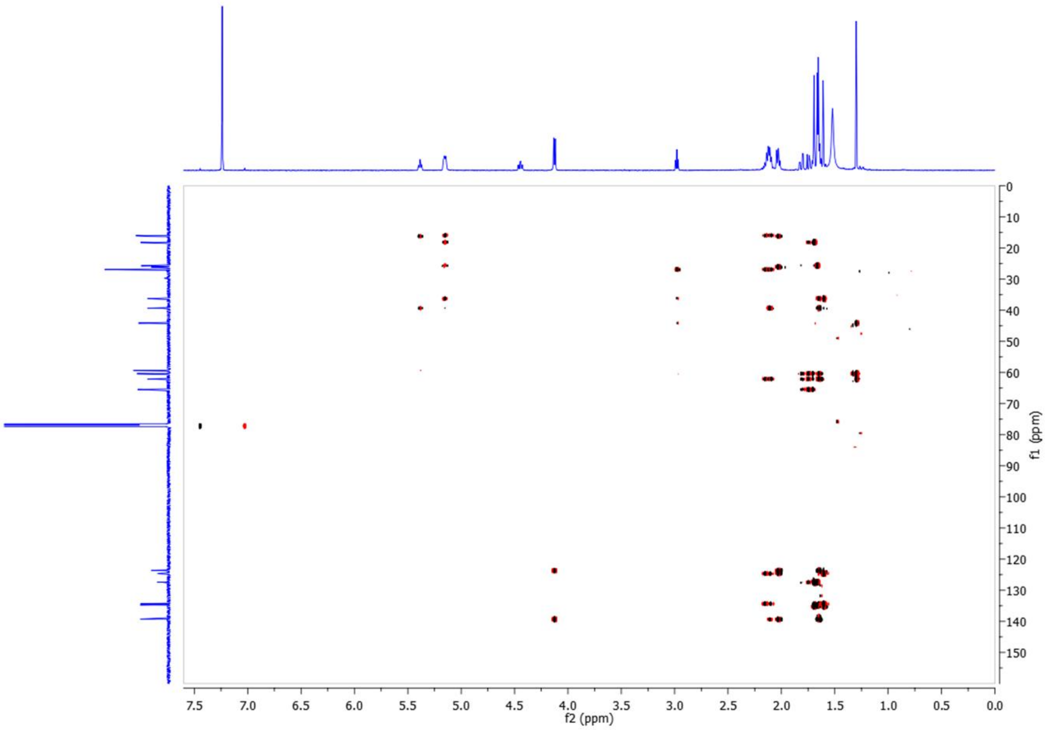

Figure S6. NOESY spectrum of compound **1** (600 MHz, $CDCl_3$).

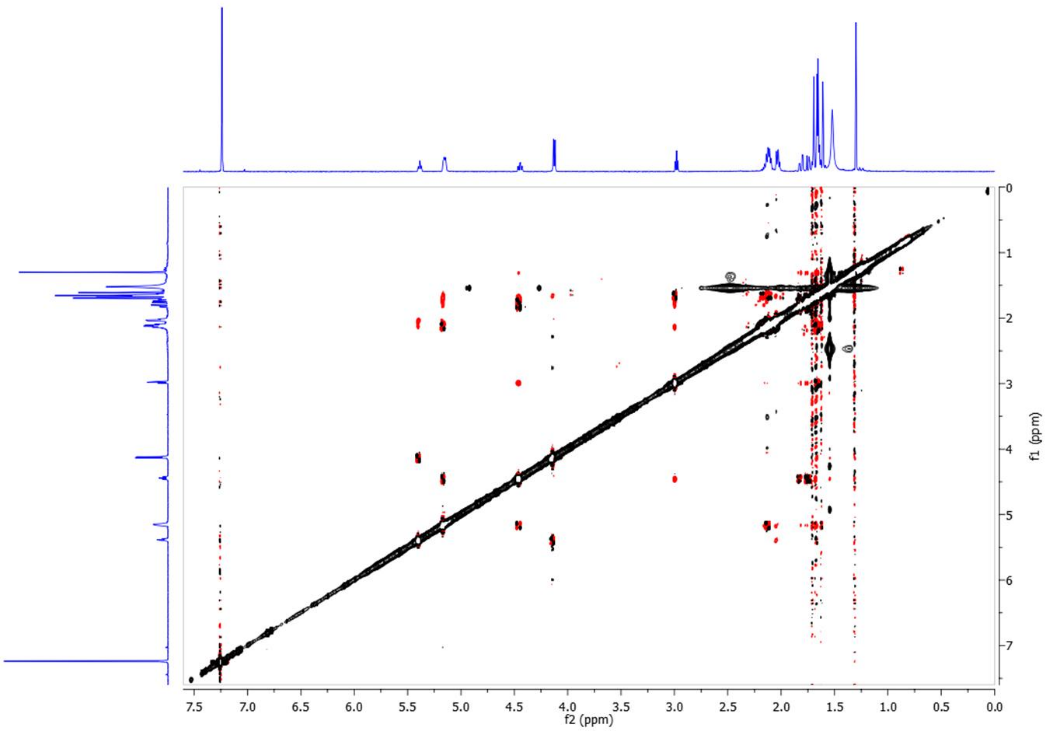

Figure S7. HR-ESIMS report of compound **1**

Qualitative Compound Report

**Data File**	bifu13_15c8-2.d	**Sample Name**	bifu13_15c8-2
**Sample Type**	Sample	**Position**	P1-A9
**Instrument Name**	Instrument 1	**User Name**	
**Acq Method**	VsmyPosFragile_BDV15.m	**Acquired Time**	09-Oct-14 02:40:49
**IRM Calibration Status**	Success	**DA Method**	Default.m
**Comment**			

**Sample Group**	**Info.**
**Fragmentor**	**Nozzle Voltage**

Compound Table

Compound Label	RT	Mass	Abund	Formula	Tgt Mass	Diff (ppm)	MFG Formula	DB Formula
Cpd 1:	6.182	322.2509	234144	C20H34O3	322.2508	0.18	C20H34O3	C20H34O3

Compound Label	*m/z*	RT	Algorithm	Mass
Cpd 1:	671.489	6.182	Find By Formula	322.2509

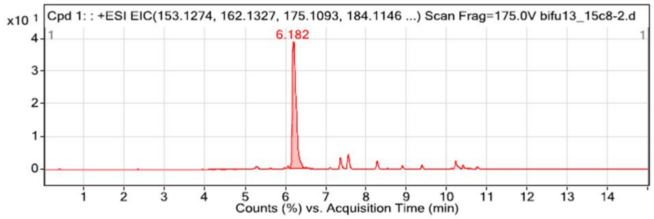

MS Spectrum

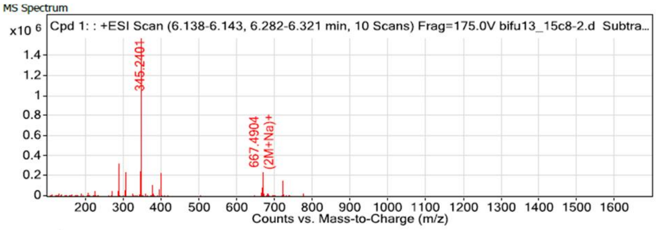

MS Zoomed Spectrum

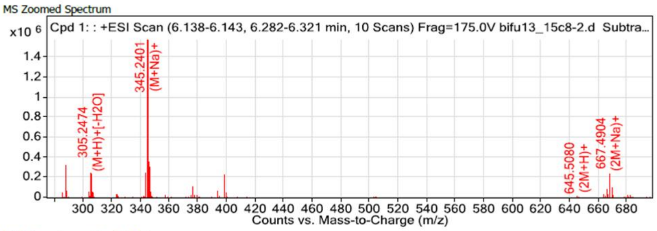

MS Spectrum Peak List

m/z	*Calc m/z*	Diff(ppm)	z	Abund	Formula	Ion
305.2474	305.2475	-0.46	1	234144.1	C20H33O2	(M+H)+[-H2O]
306.2505	306.2509	-1.39	1	49374.8	C20H33O2	(M+H)+[-H2O]
323.2576	323.2581	-1.54	1	26931.7	C20H35O3	(M+H)+
345.2401	345.24	0.18	1	1573659.6	C20H34NaO3	(M+Na)+
345.2401				1573659.6		
346.2433	346.2434	-0.3	1	309987.1	C20H34NaO3	(M+Na)+
627.4969	627.4983	-2.22	1	1725.2	C40H67O5	(2M+H)+[-H2O]
645.508	645.5089	-1.31	1	8610.9	C40H69O6	(2M+H)+
667.4904	667.4908	-0.65	1	238402.5	C40H68NaO6	(2M+Na)+
668.4938	668.4942	-0.64	1	100601	C40H68NaO6	(2M+Na)+

--- End Of Report ---

Figure S8. FT-IR spectrum of compound **1**.

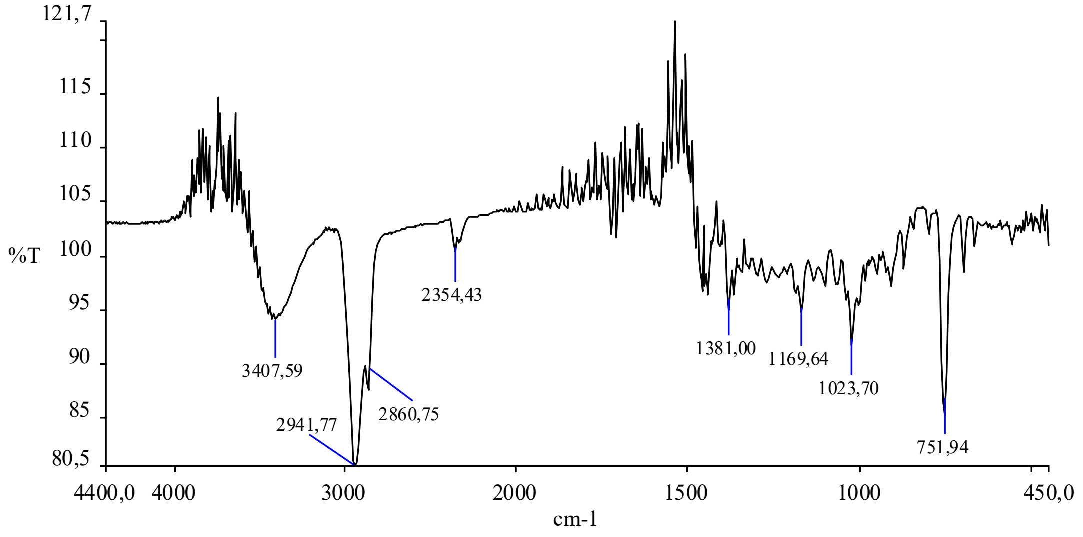

Figure S9. ^{1}H NMR spectrum of compound **2** (600 MHz, CD_6D_6).

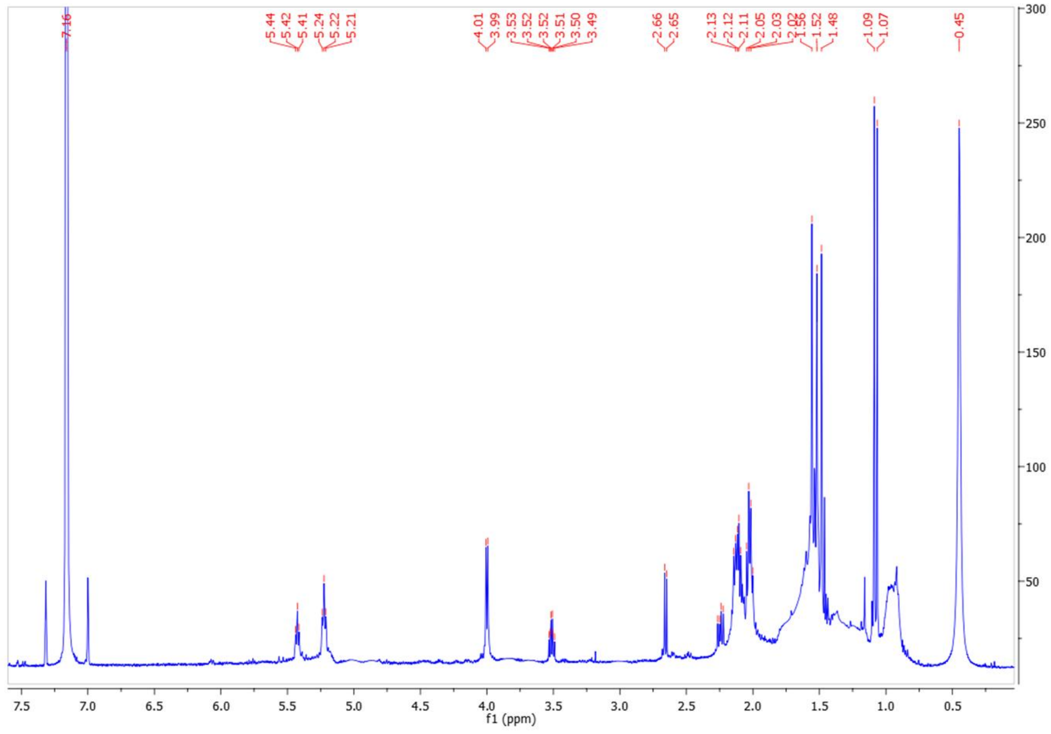

Figure S10. ^{13}C NMR spectrum of compound **2** (150 MHz, C_6D_6).

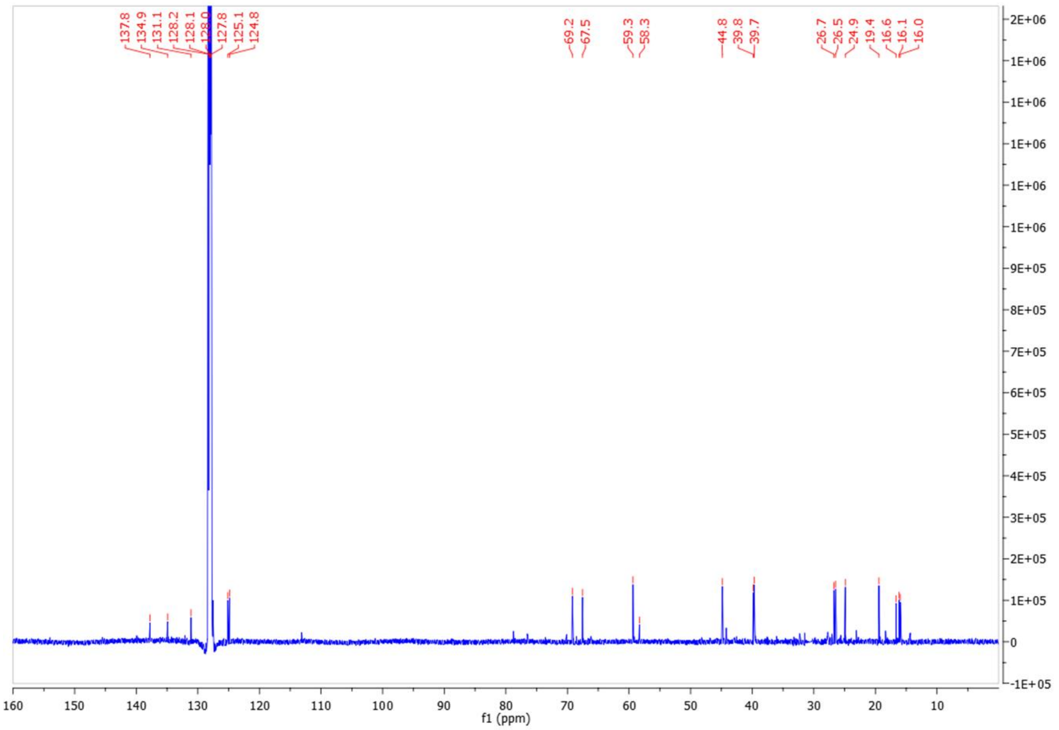

Figure S11. *g*HSQC spectrum of compound **2** (600/150 MHz, C_6D_6)

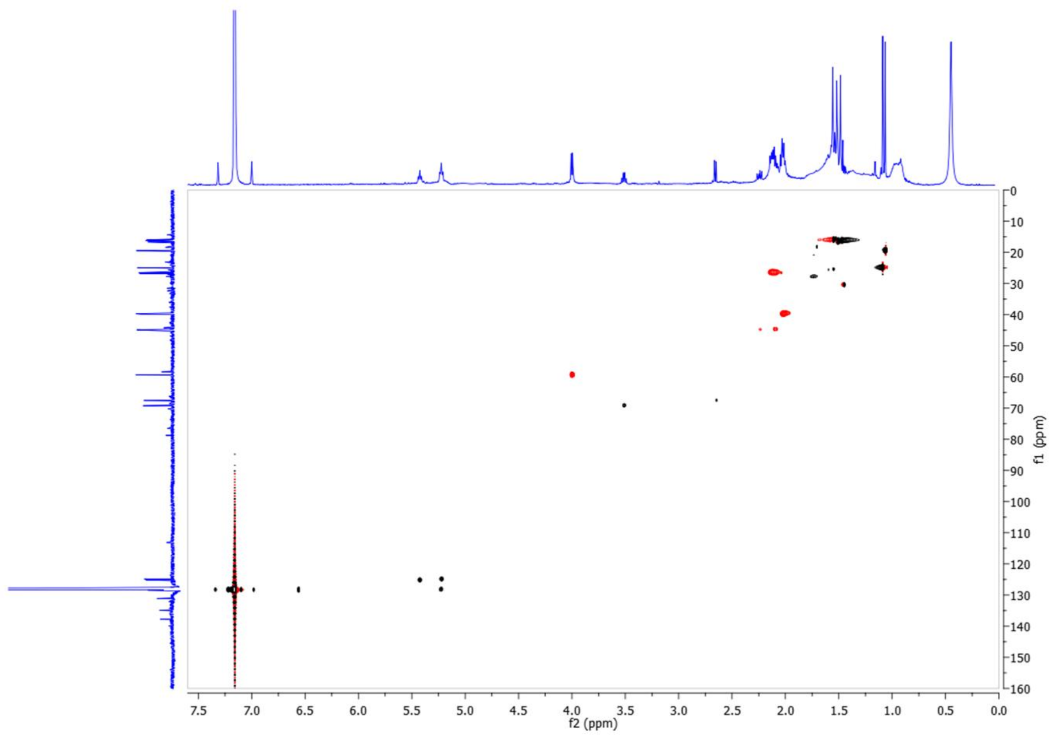

Figure S12. *g*COSY spectrum of compound **2** (600 MHz, CD_6D_6).

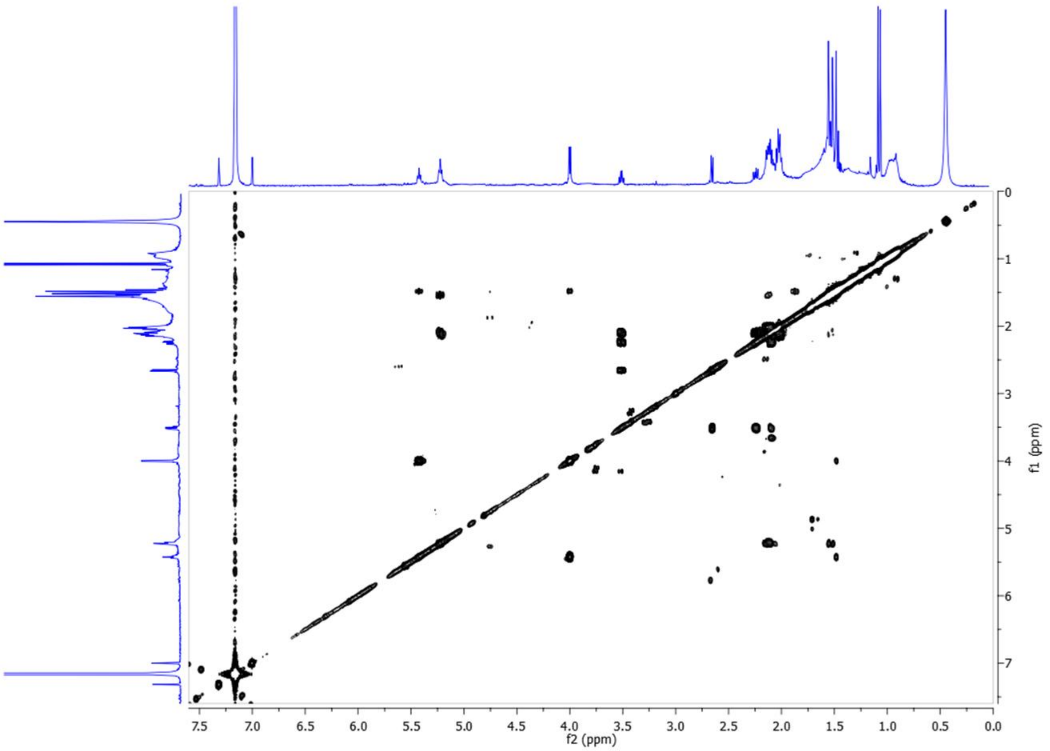

Figure S13. *g*HMBC spectrum of compound **2** (600/150 MHz, CD_6D_6).

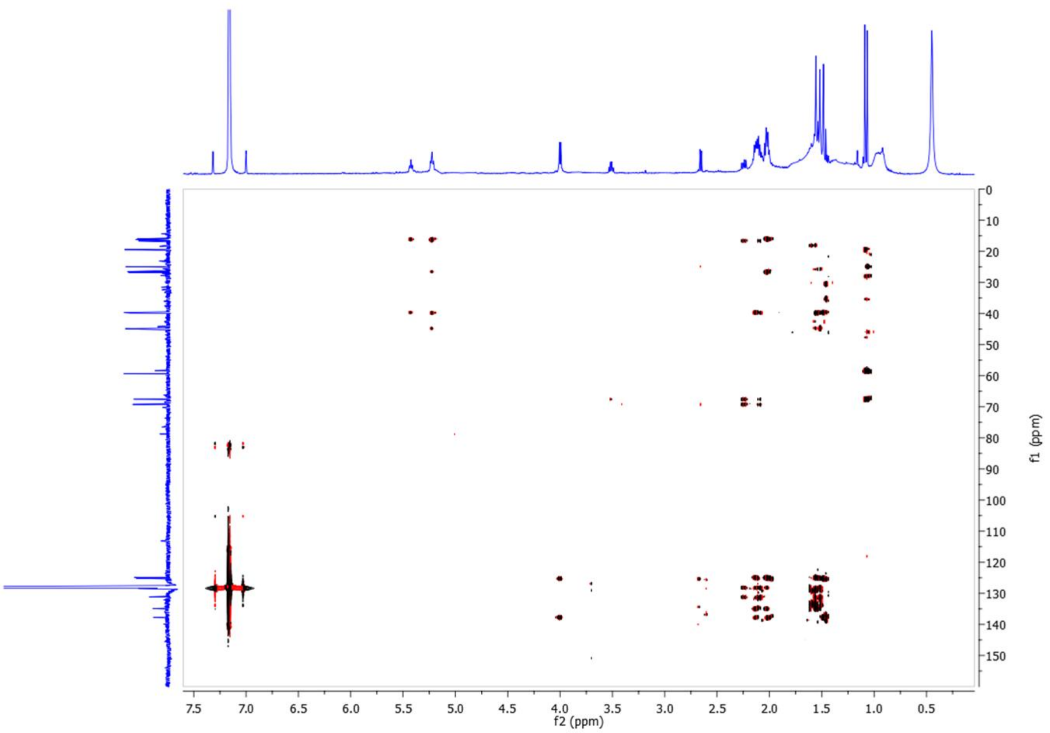

Figure S14. NOESY spectrum of compound **2** (600 MHz, CD_6D_6).

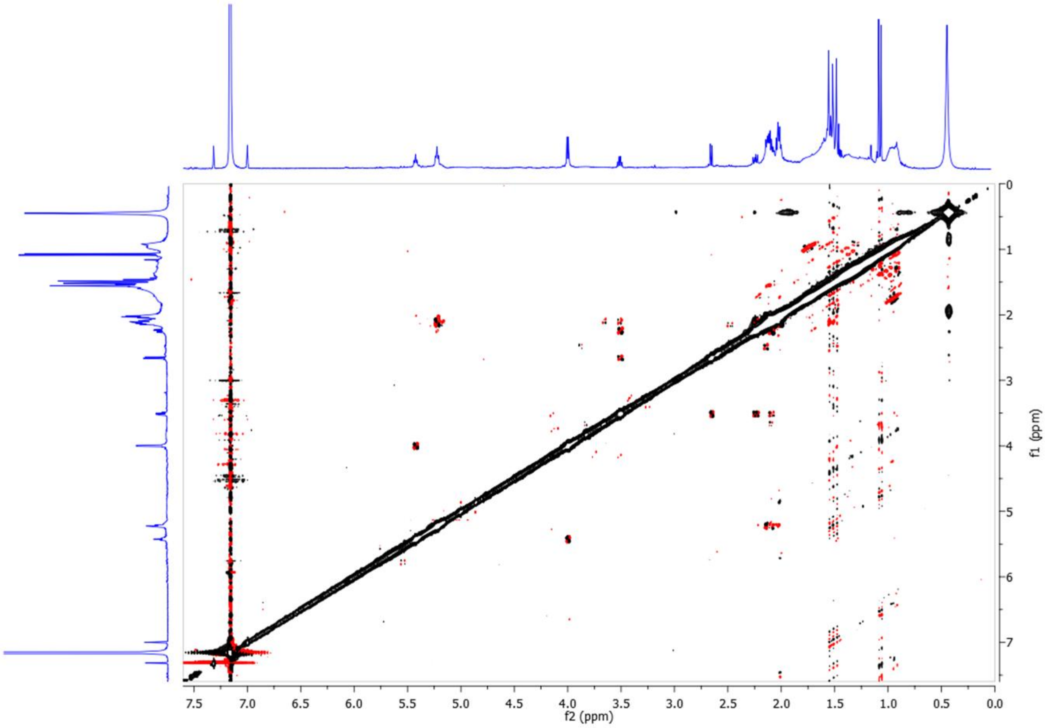

Figure S15. HR-ESIMS report of compound **2**

Qualitative Compound Report

**Data File**	bifu20_15c10-8.d	**Sample Name**	bifu20_15c10-8
**Sample Type**	Sample	**Position**	P1-A5
**Instrument Name**	Instrument 1	**User Name**	
**Acq Method**	VsmyPosFragile_BDV15.m	**Acquired Time**	14-Oct-14 02:00:04
**IRM Calibration Status**	Success	**DA Method**	Default.m
**Comment**			

**Sample Group**	**Info.**
**Fragmentor**	**Nozzle Voltage**

Compound Table

Compound Label	RT	Mass	Abund	Formula	Tgt Mass	Diff (ppm)	MFG Formula	DB Formula
Cpd 1:	6.407	322.2507	1343307	C20H34O3	322.2508	-0.16	C20H34O3	C20H34O3

Compound Label	*m/z*	RT	Algorithm	Mass
Cpd 1:	989.7363	6.407	Find By Formula	322.2507

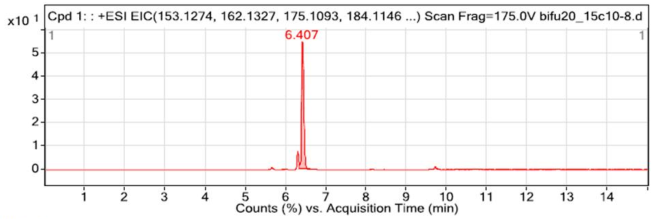

MS Spectrum

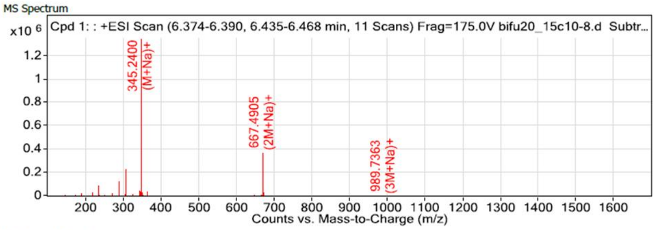

MS Zoomed Spectrum

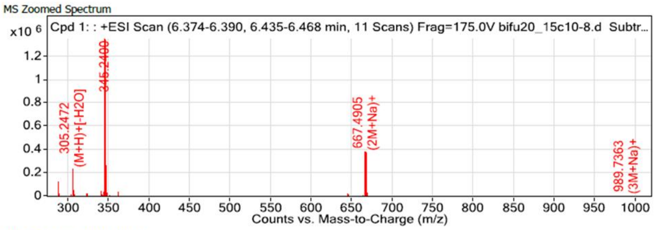

MS Spectrum Peak List

m/z	*Calc m/z*	Diff(ppm)	z	Abund	Formula	Ion
305.2472	305.2475	-0.93	1	228581	C20H33O2	(M+H)+[-H2O]
323.2574	323.2581	-1.96	1	18612.9	C20H35O3	(M+H)+
327.2267	327.2295	-8.54	1	75.7	C20H32NaO2	(M+Na)+[-H2O]
345.24				1343307		
345.24	345.24	-0.13	1	1343307	C20H34NaO3	(M+Na)+
346.2433	346.2434	-0.4	1	271910.8	C20H34NaO3	(M+Na)+
627.4971	627.4983	-1.89	1	1103	C40H67O5	(2M+H)+[-H2O]
645.5082	645.5089	-0.97	1	16929	C40H69O6	(2M+H)+
667.4905	667.4908	-0.45	1	373014.7	C40H68NaO6	(2M+Na)+
989.7363	989.7416	-5.38	1	70	C60H102NaO9	(3M+Na)+

--- End Of Report ---

Figure S16. FT-IR spectrum of compound **2**.

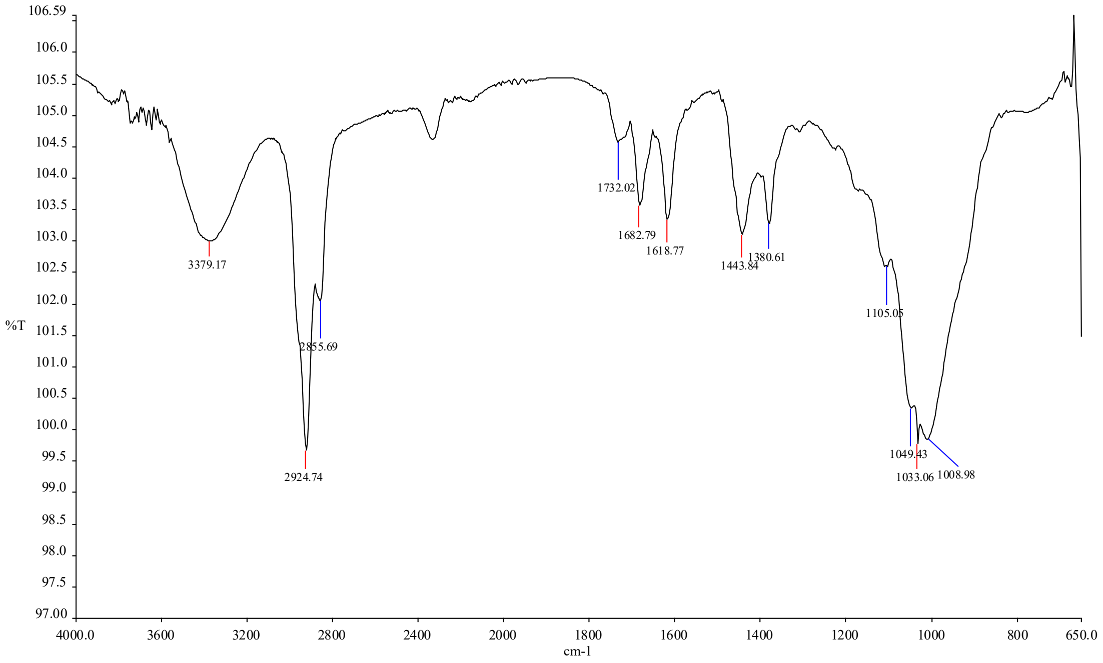

Figure S17. ^{1}H NMR spectrum of compound **3** (500 MHz, $CDCl_3$)

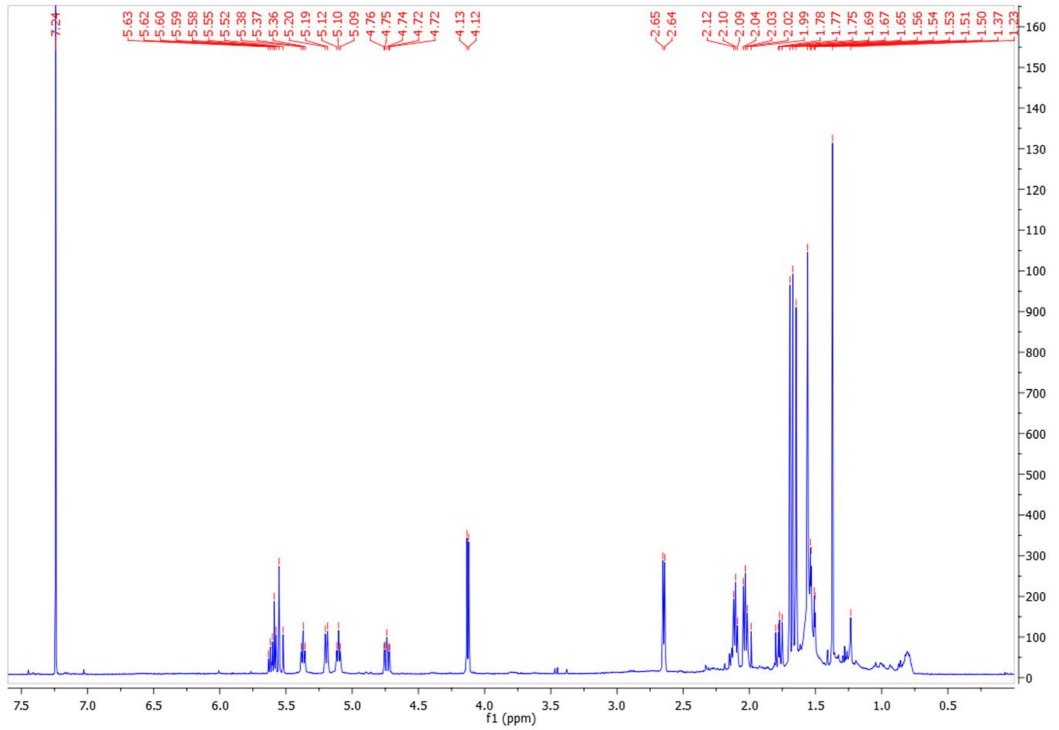

Figure S18. ^{13}C NMR spectrum of compound **3** (125 MHz, $CDCl_3$).

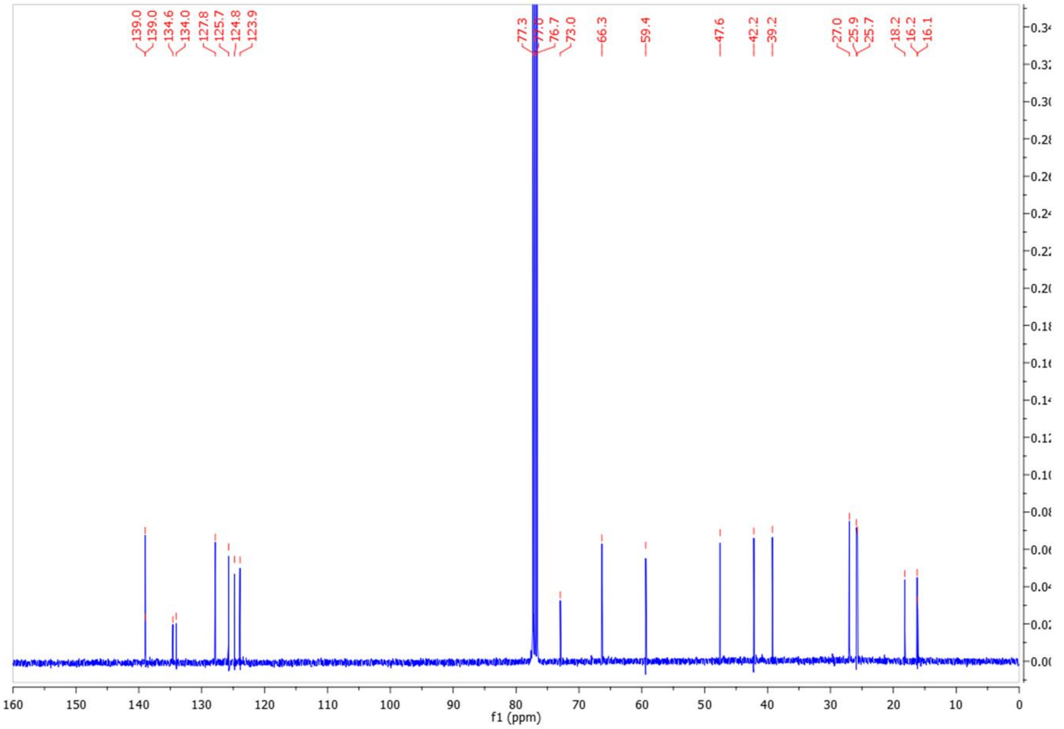

Figure S19. *g*HSQC spectrum of compound **3** (500/125 MHz, $CDCl_3$).

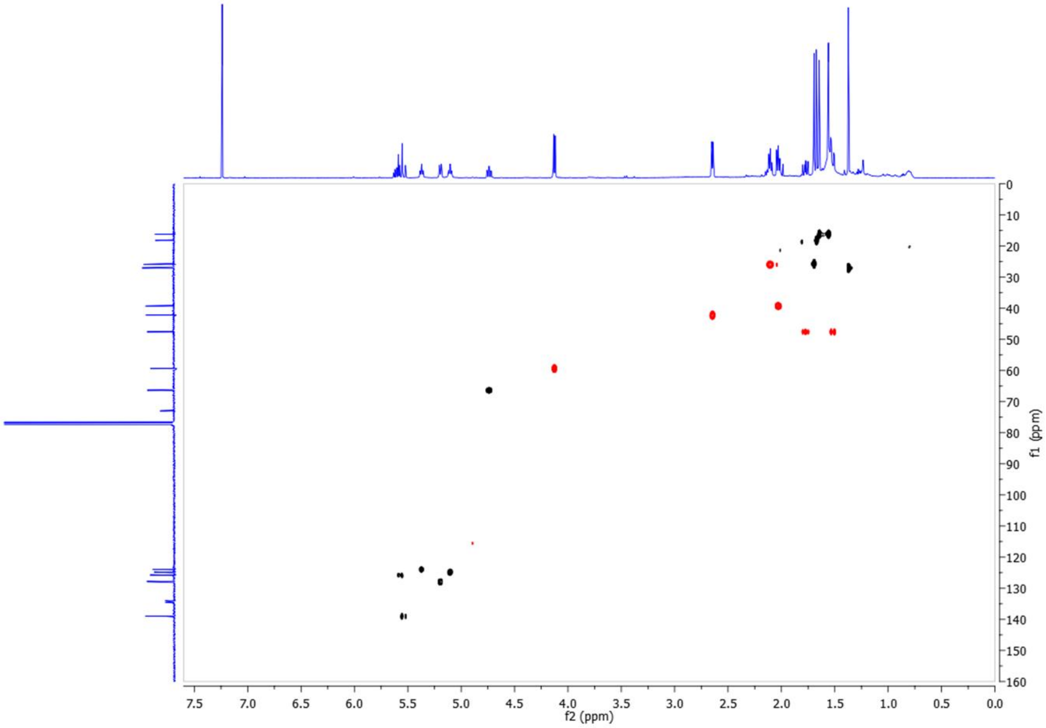

Figure S20. *g*COSY spectrum of compound **3** (500 MHz, $CDCl_3$).

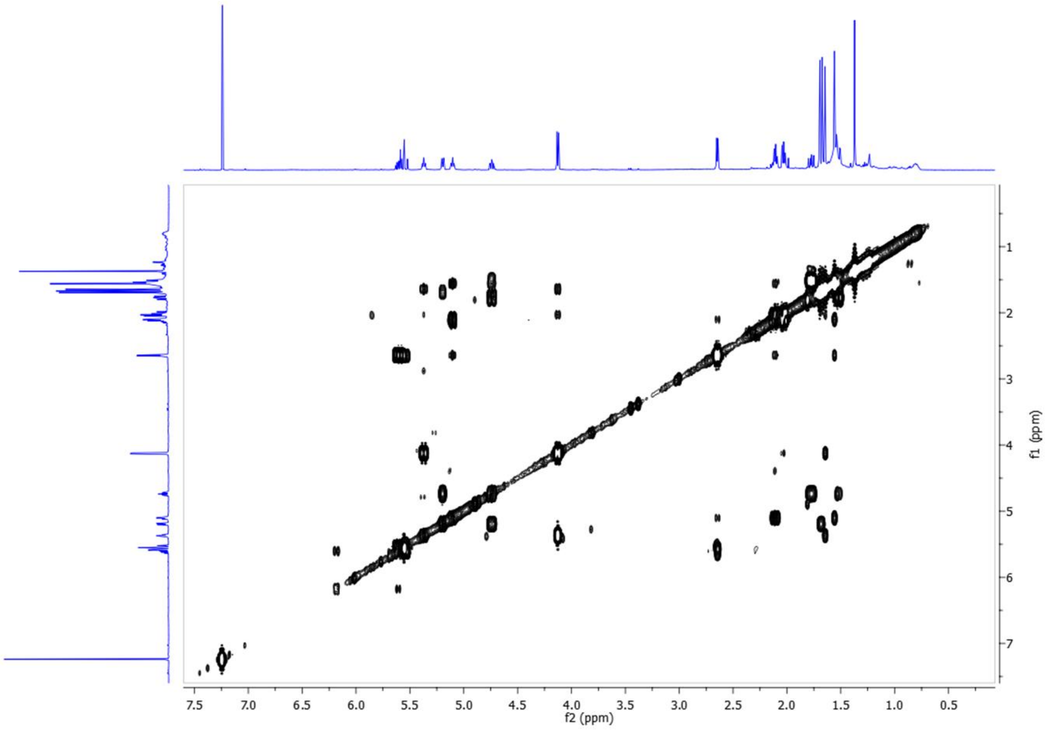

Figure S21. *g*HMBC spectrum of compound **3** (500/125 MHz, $CDCl_3$).

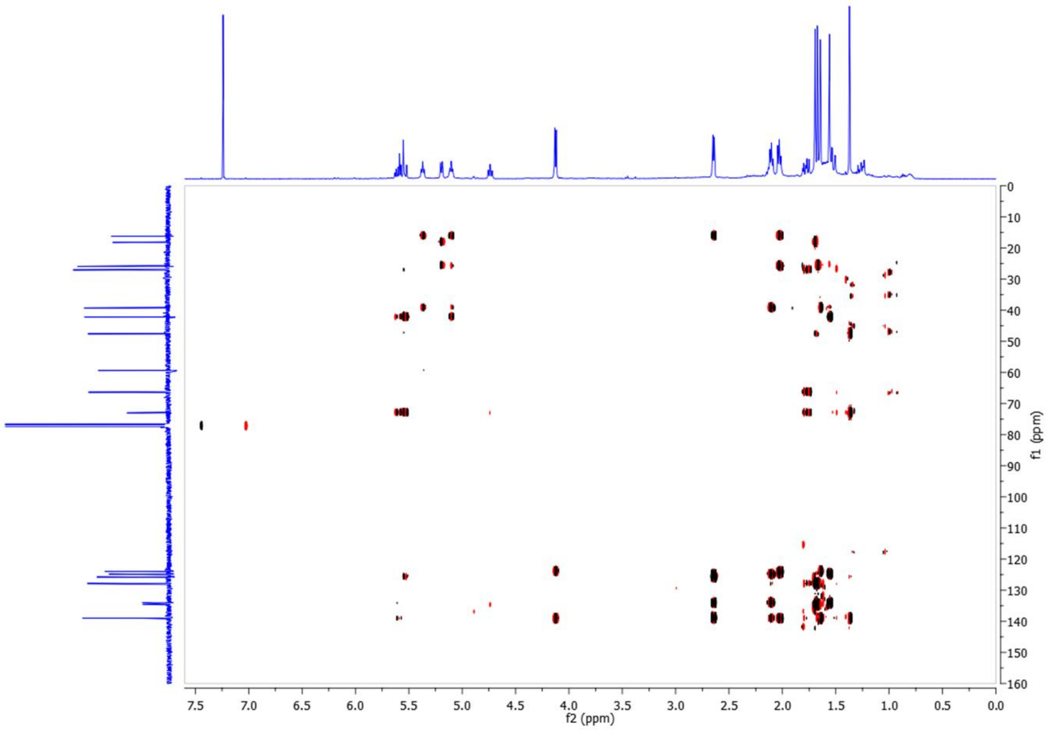

Figure S22. NOESY spectrum of compound **3** (500 MHz, $CDCl_3$).

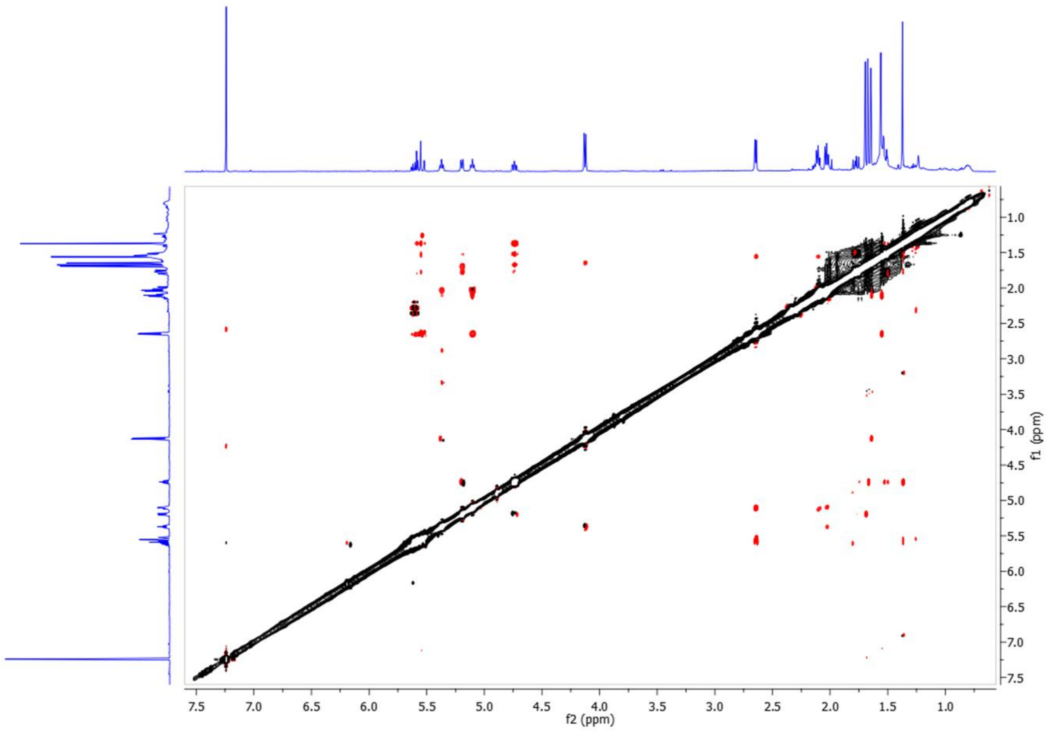

Figure S23. HR-ESIMS report of compound **3**.

Qualitative Compound Report

**Data File**	bifu19_15c10-7.d	**Sample Name**	bifu19_15c10-7
**Sample Type**	Sample	**Position**	P1-B4
**Instrument Name**	Instrument 1	**User Name**	
**Acq Method**	VsmyPosFragile_BDV15.m	**Acquired Time**	09-Oct-14 04:04:10
**IRM Calibration Status**	Success	**DA Method**	Default.m
**Comment**			

**Sample Group**	**Info.**
**Fragmentor**	**Nozzle Voltage**

Compound Table

Compound Label	RT	Mass	Abund	Formula	Tgt Mass	Diff (ppm)	MFG Formula	DB Formula
Cpd 1:	6.315	322.2506	993579	C20H34O3	322.2508	-0.58	C20H34O3	C20H34O3

Compound Label	*m/z*	RT	Algorithm	Mass
Cpd 1:	672.5173	6.315	Find By Formula	322.2506

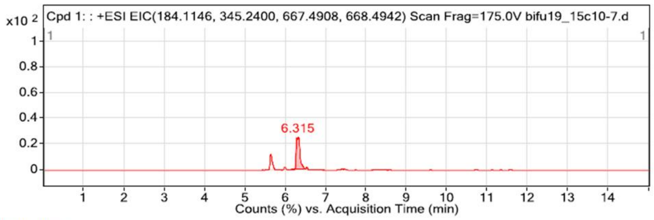

MS Spectrum

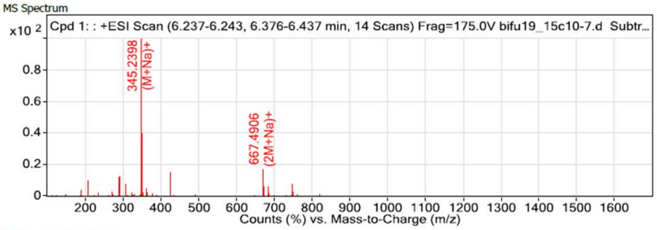

MS Zoomed Spectrum

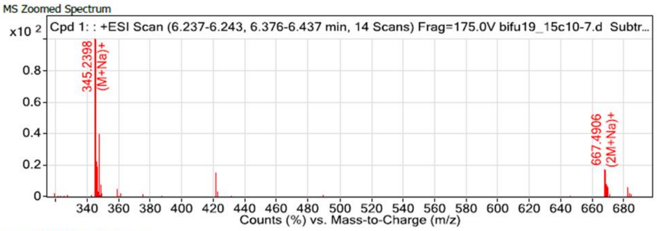

MS Spectrum Peak List

m/z	*Calc m/z*	Diff(ppm)	z	Abund	Formula	Ion
345.2398	345.24	0.55	1	993579.4	C20H34NaO3	(M+Na)+
346.2435	346.2434	-0.19	1	193862.7	C20H34NaO3	(M+Na)+
667.4906	667.4908	0.28	1	170803.8	C40H68NaO6	(2M+Na)+
668.4941	668.4942	0.25	1	73326.5	C40H68NaO6	(2M+Na)+
669.5042	669.4973	-10.19	1	60326.6	C40H68NaO6	(2M+Na)+
670.5084	670.5003	-12.11	1	21219.4	C40H68NaO6	(2M+Na)+
671.5143	671.5032	-16.5	1	6672.3	C40H68NaO6	(2M+Na)+
672.5173	672.506	-16.83	1	1745.8	C40H68NaO6	(2M+Na)+

--- End Of Report ---

Figure S24. FT-IR spectrum of compound **3**.

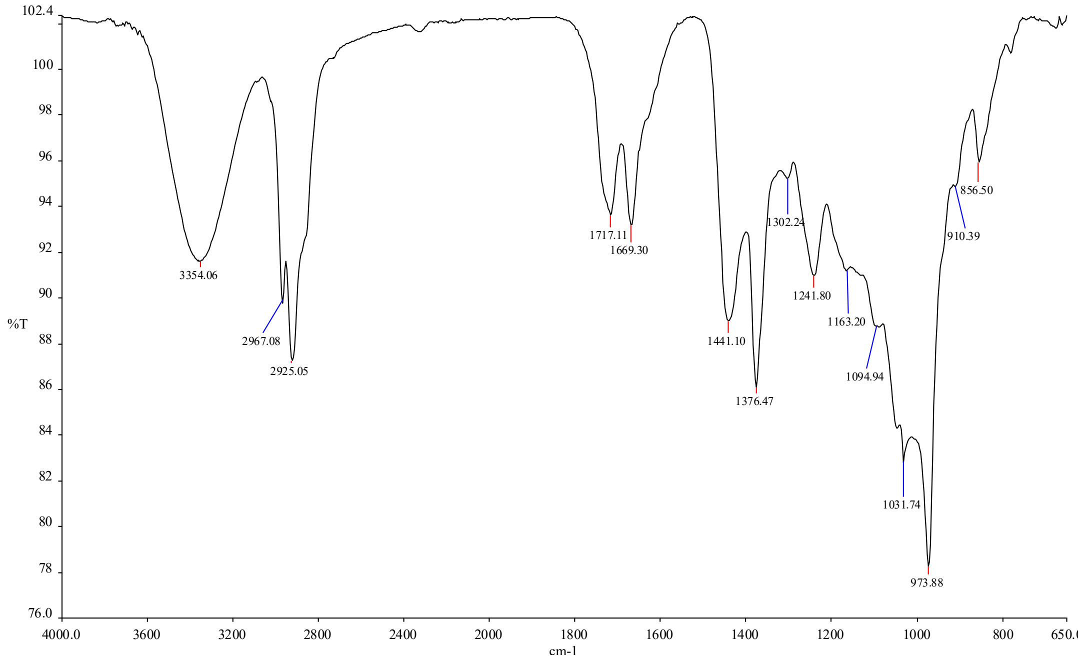

Figure S25. ^{1}H NMR spectrum of compound **4** (500 MHz, $CDCl_3$).

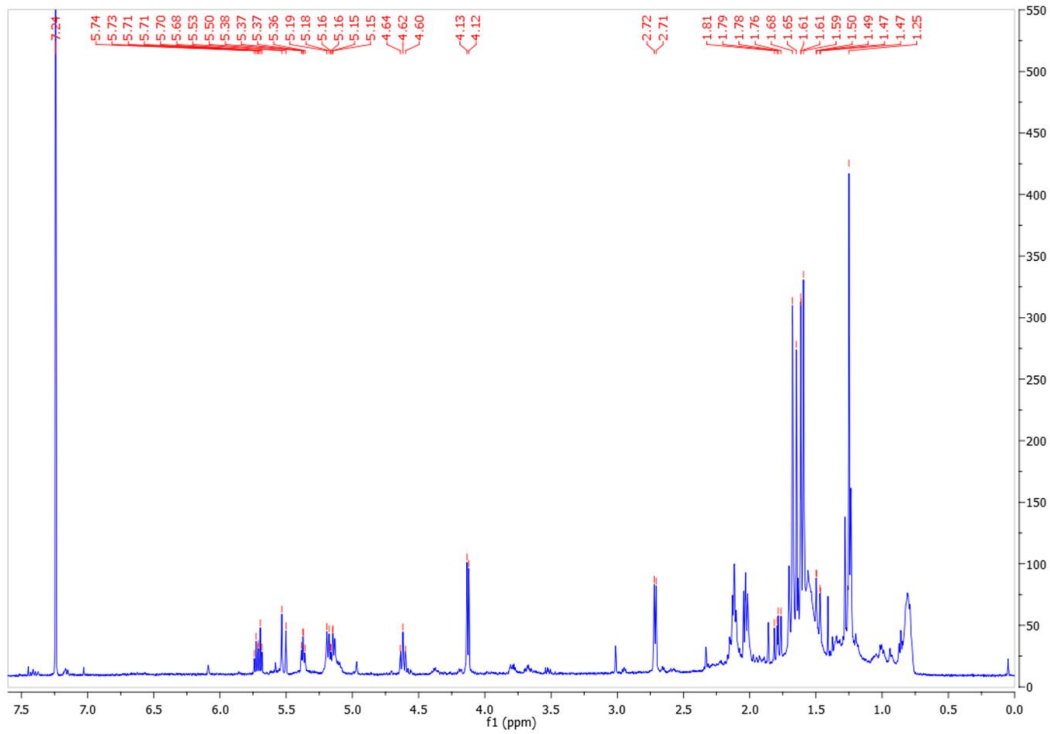

Figure S26. *g*HSQC spectrum of compound **4** (500/125 MHz, $CDCl_3$)

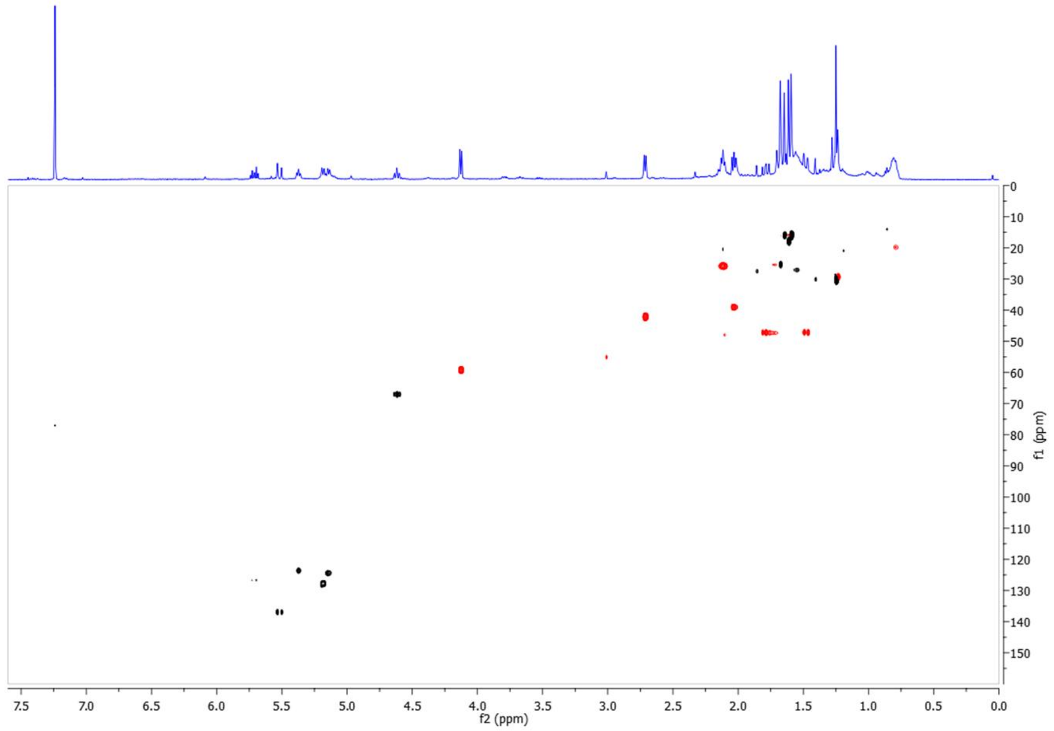

Figure S27. *g*COSY spectrum of compound **4** (500 MHz, CDCl3)

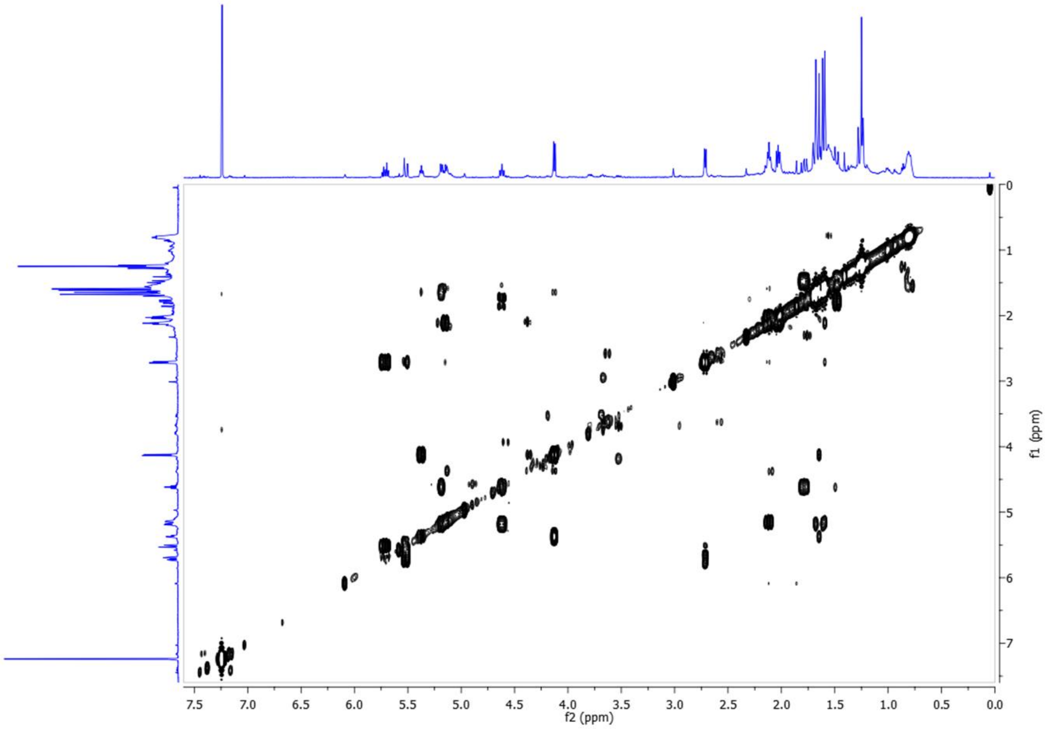

Figure S28. ^{1}H NMR spectrum of compound **4** (600 MHz, C_6D_6)

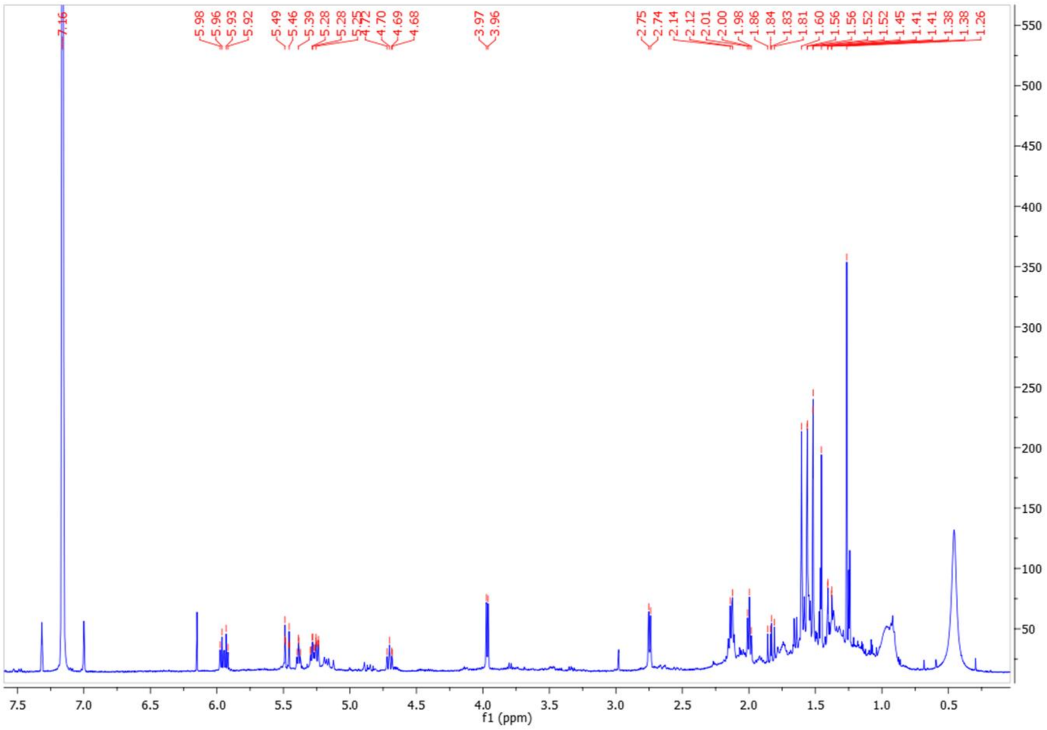

Figure S29. ^{13}C NMR spectrum of compound **4** (150 MHz, C_6D_6)

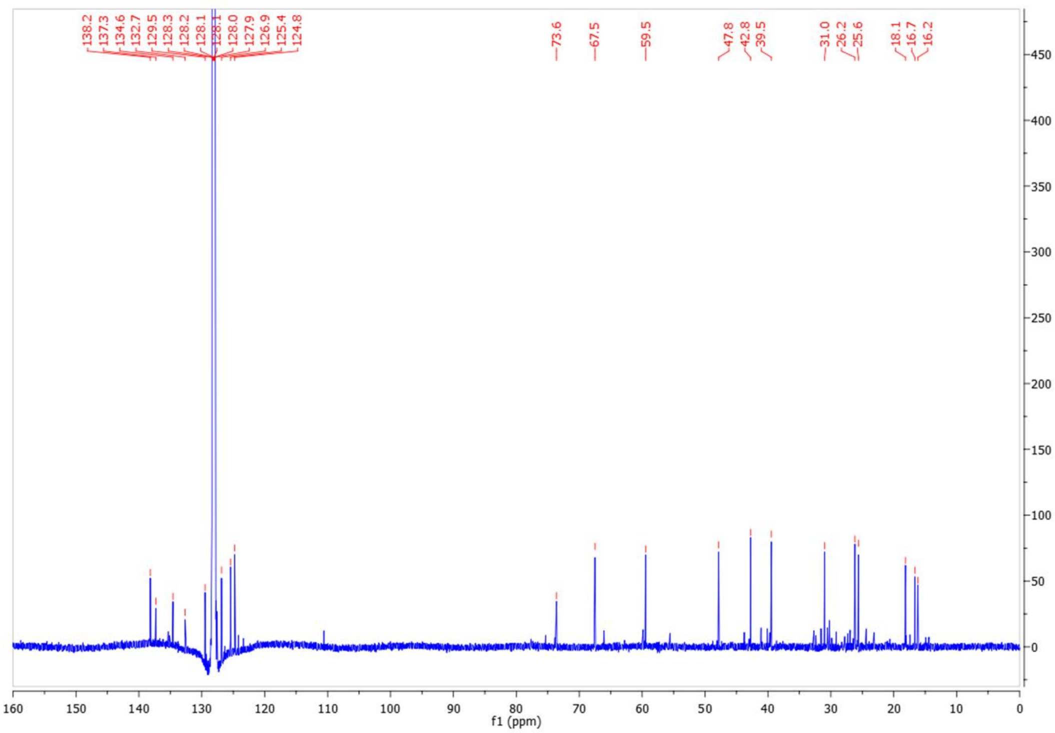

Figure S30. *g*HMBC spectrum of compound **4** (600/150 MHz, C_6D_6)

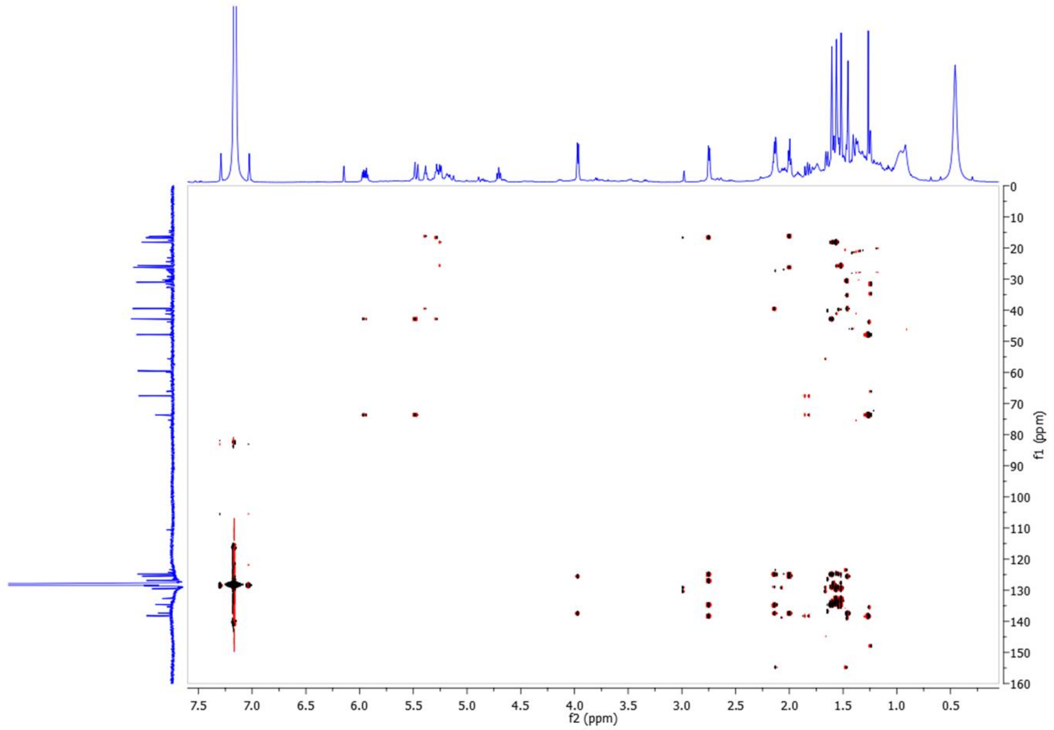

Figure S31. NOESY spectrum of compound **4** (600 MHz, C_6D_6)

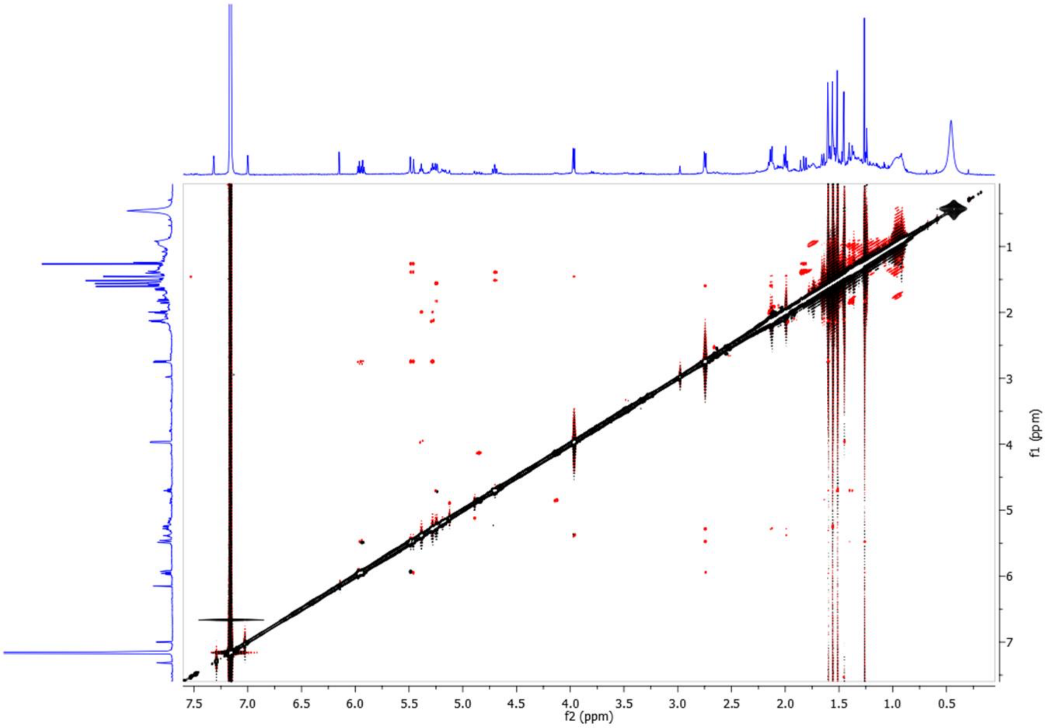

Figure S32. HR-ESIMS report of compound **4**

Qualitative Compound Report

**Data File**	bifu22_15c10-10.d	**Sample Name**	bifu22_15c10-10
**Sample Type**	Sample	**Position**	P1-A1
**Instrument Name**	Instrument 1	**User Name**	
**Acq Method**	VsmyPosFragile_BDV15.m	**Acquired Time**	14-Oct-14 00:36:39
**IRM Calibration Status**	Success	**DA Method**	Default.m
**Comment**			

**Sample Group**	**Info.**
**Fragmentor**	**Nozzle Voltage**

Compound Table

Compound Label	RT	Mass	Abund	Formula	Tgt Mass	Diff (ppm)	MFG Formula	DB Formula
Cpd 1:	6.517	322.2507	1352352	C20H34O3	322.2508	-0.4	C20H34O3	C20H34O3

Compound Label	*m/z*	RT	Algorithm	Mass
Cpd 1:	992.7119	6.517	Find By Formula	322.2507

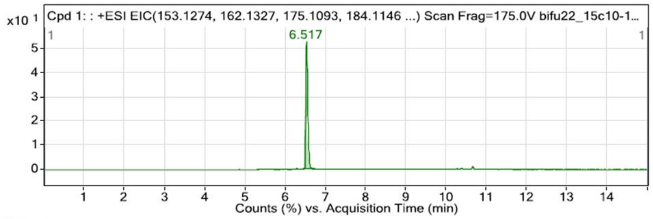

MS Spectrum

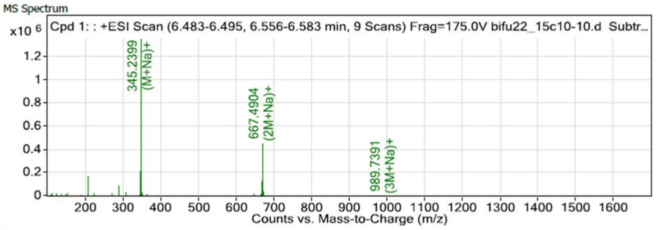

MS Zoomed Spectrum

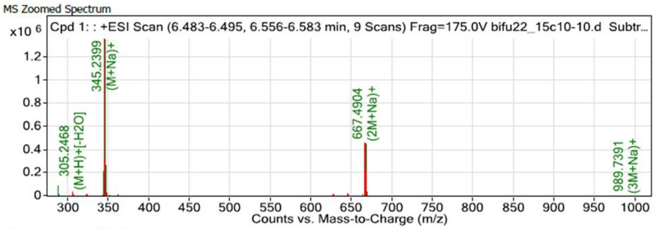

MS Spectrum Peak List

m/z	*Calc m/z*	Diff(ppm)	z	Abund	Formula	Ion
305.2468	305.2475	-2.3	1	31845.6	C20H33O2	(M+H)+[-H2O]
323.2581	323.2581	-0.02	1	4878.3	C20H35O3	(M+H)+
345.2399	345.24	-0.35	1	1352352	C20H34NaO3	(M+Na)+
345.2399				1352352		
346.2432	346.2434	-0.55	1	270176	C20H34NaO3	(M+Na)+
627.4974	627.4983	-1.45	1	6121.5	C40H67O5	(2M+H)+[-H2O]
645.5079	645.5089	-1.54	1	23284.2	C40H69O6	(2M+H)+
649.4759	649.4802	-6.69	1	231.6	C40H66NaO5	(2M+Na)+[-H2O]
667.4904	667.4908	-0.55	1	452882.8	C40H68NaO6	(2M+Na)+
989.7391	989.7416	-2.52	1	463.4	C60H102NaO9	(3M+Na)+

--- End Of Report ---

Figure S33. FT-IR spectrum of compound **4**

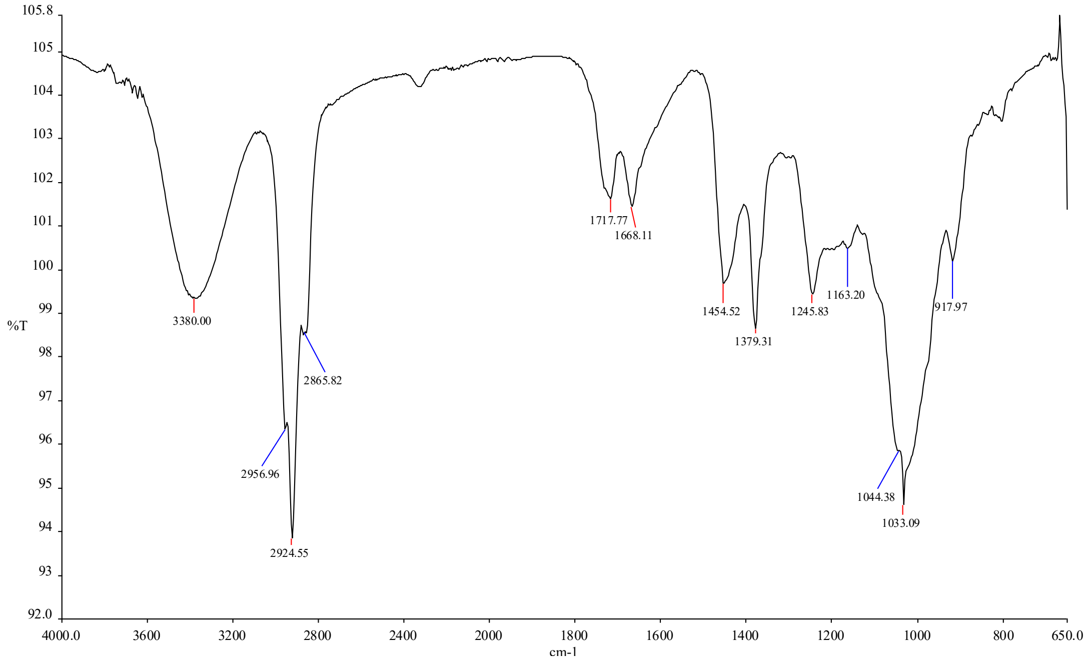

Table S1. Cartesian coordinates, energies, and population at 298 K of significantly populated conformers of model compound **1r**. Conformers were optimized at the B3LYP/6-31G(d,p)/SMD($CHCl_3$) level.

Conformer 1				Conformer 2				Conformer 3			
Relative energy (kcal/mol)			0.00	Relative energy (kcal/mol)			0.21	Relative energy (kcal/mol)			0.66
Population (%)			29.7	Population (%)			20.9	Population (%)			9.8
C	4.48946	-0.64417	-0.02386	C	-4.19271	-0.53049	-0.90456	C	-4.65614	-0.25441	0.58232
H	4.43111	-1.55639	-0.63262	H	-3.66198	-1.48925	-0.96968	H	-5.59186	0.29897	0.73031
H	4.24321	-0.94181	1.00299	H	-4.08820	-0.05502	-1.88896	H	-4.57497	-0.94722	1.43098
C	3.43578	0.36165	-0.51344	C	-3.52153	0.35109	0.15990	C	-3.48911	0.74767	0.63814
C	2.04077	-0.21434	-0.48669	C	-2.08061	0.65716	-0.17468	C	-2.13060	0.08578	0.64085
C	0.79838	0.52072	-0.16637	C	-0.89657	-0.07989	0.32016	C	-0.86749	0.69666	0.17363
C	-0.52754	-0.00385	-0.71523	C	0.38695	-0.04932	-0.50813	C	0.45280	0.19776	0.75957
C	-1.54635	-0.37533	0.38868	C	1.59026	0.56965	0.24269	C	1.40923	-0.40053	-0.29970
C	-2.88602	-0.72197	-0.20255	C	2.85948	0.42247	-0.55211	C	2.75260	-0.71723	0.29971
C	-4.03075	-0.02327	-0.14708	C	3.92136	-0.35798	-0.29742	C	3.92904	-0.10700	0.08678
C	-5.27696	-0.53448	-0.82903	C	5.10183	-0.37938	-1.23845	C	5.17335	-0.56474	0.80903
C	0.80637	1.98474	0.21593	C	-1.01562	-1.23608	1.28867	C	-0.82198	2.08259	-0.43277
C	-4.20910	1.29696	0.56147	C	4.06204	-1.27151	0.89511	C	4.14766	1.05766	-0.84727
O	-1.09275	-1.52991	1.10614	O	1.37768	1.97362	0.43682	O	0.87416	-1.63069	-0.80421
O	1.42212	-0.37051	0.81720	O	-1.26563	1.22450	0.88036	O	-1.56670	-0.30337	-0.63891
C	5.91303	-0.08566	-0.07278	C	-5.67424	-0.78285	-0.61572	C	-4.73907	-1.05088	-0.72341
H	6.64078	-0.82196	0.28495	H	-6.12727	-1.41382	-1.38788	H	-5.60573	-1.72116	-0.71740
H	6.19735	0.19309	-1.09446	H	-5.81190	-1.28666	0.34814	H	-4.84026	-0.38419	-1.58786
H	6.01138	0.80881	0.55355	H	-6.23774	0.15686	-0.58103	H	-3.84527	-1.66165	-0.88377
H	3.48701	1.27056	0.09424	H	-4.06042	1.30550	0.23666	H	-3.56559	1.44947	-0.19845
H	3.65991	0.65548	-1.54860	H	-3.59203	-0.12408	1.14343	H	-3.56842	1.33884	1.56090
H	1.91829	-1.09781	-1.11848	H	-1.95128	1.16786	-1.13227	H	-2.02482	-0.69217	1.40169
H	-0.96115	0.76140	-1.36862	H	0.63299	-1.07412	-0.80784	H	0.94719	1.03477	1.26518
H	-0.35953	-0.89794	-1.32629	H	0.23412	0.53115	-1.42511	H	0.26769	-0.57535	1.51391
H	-1.64228	0.47641	1.07789	H	1.68949	0.07015	1.21761	H	1.51810	0.32239	-1.12138
H	-2.88440	-1.66744	-0.74640	H	2.87870	1.04221	-1.44949	H	2.72458	-1.55313	0.99988
H	-6.09129	-0.67156	-0.10491	H	6.02270	-0.08291	-0.71856	H	5.94933	-0.87314	0.09562
H	-5.64392	0.18569	-1.57292	H	5.27901	-1.39161	-1.62662	H	5.60743	0.25071	1.40339
H	-5.10650	-1.48907	-1.33496	H	4.96006	0.29172	-2.09037	H	4.97343	-1.40531	1.47965
H	0.72549	2.61162	-0.67918	H	-1.10108	-2.18204	0.74241	H	-0.67532	2.83460	0.35066
H	1.71557	2.25881	0.75325	H	-1.88174	-1.13163	1.94417	H	-1.73763	2.31959	-0.97661
H	-0.04798	2.21530	0.86141	H	-0.12270	-1.29786	1.92019	H	0.01625	2.16520	-1.13306
H	-5.05015	1.24292	1.26489	H	4.99735	-1.06101	1.42986	H	4.95657	0.83345	-1.55470
H	-4.45907	2.08939	-0.15684	H	4.12083	-2.31992	0.57295	H	4.46661	1.94680	-0.28681
H	-3.32550	1.61773	1.11667	H	3.23983	-1.18785	1.60834	H	3.26081	1.32783	-1.42375
H	-0.13561	-1.40587	1.23348	H	0.44102	2.07006	0.68377	H	-0.07950	-1.47677	-0.92466

Conformer 4				Conformer 5				Conformer 6			
Relative energy (kcal/mol)			0.87	Relative energy (kcal/mol)			0.92	Relative energy (kcal/mol)			1.21
Population (%)			6.8	Population (%)			6.2	Population (%)			3.9
C	4.61503	-0.07838	0.22102	C	4.45683	0.46688	-0.72760	C	4.32649	-0.38154	-0.84888
H	4.34493	-0.20769	1.27657	H	4.40374	0.16898	-1.78348	H	5.28504	0.09334	-1.09095
H	5.52311	0.53681	0.20865	H	5.51360	0.39225	-0.44446	H	3.74482	-0.38626	-1.77998
C	3.49376	0.71111	-0.48090	C	3.65984	-0.53901	0.12073	C	3.61197	0.49875	0.19359
C	2.14460	0.03703	-0.39716	C	2.21635	-0.66748	-0.31258	C	2.20808	0.04000	0.51661
C	0.84648	0.71482	-0.18576	C	1.02796	-0.13394	0.39216	C	0.96978	0.51811	-0.13891
C	-0.42694	0.02709	-0.67650	C	-0.24098	0.13982	-0.41395	C	-0.26039	-0.38851	-0.13289
C	-1.43719	-0.28147	0.45448	C	-1.46395	-0.68077	0.06130	C	-1.48217	0.23315	0.58492
C	-2.73097	-0.81137	-0.10279	C	-2.71618	-0.25581	-0.65713	C	-2.70476	-0.62756	0.41609
C	-3.92542	-0.20304	-0.17113	C	-3.76180	0.43627	-0.17833	C	-3.81571	-0.37454	-0.29346
C	-5.11035	-0.90424	-0.79062	C	-4.92650	0.78585	-1.07312	C	-4.93433	-1.38669	-0.35496
C	0.73863	2.21185	0.00603	C	1.13606	0.60996	1.70488	C	0.98515	1.58013	-1.21656
C	-4.22042	1.18929	0.32973	C	-3.89777	0.93397	1.23964	C	-4.07414	0.88398	-1.08463
O	-0.91034	-1.29356	1.32136	O	-1.26617	-2.06997	-0.22912	O	-1.22746	0.32972	1.99204
O	1.51375	0.00569	0.91056	O	1.38070	-1.55380	0.47264	O	1.35346	0.99638	1.19318
C	4.92498	-1.44222	-0.40342	C	3.99592	1.92035	-0.58342	C	4.58526	-1.82335	-0.39818
H	5.76673	-1.92194	0.10767	H	4.62056	2.59044	-1.18415	H	5.15580	-2.37147	-1.15579
H	4.07320	-2.12774	-0.33870	H	2.96026	2.05388	-0.91484	H	5.16214	-1.84935	0.53396
H	5.19350	-1.34370	-1.46226	H	4.05855	2.25576	0.45841	H	3.65547	-2.37668	-0.22812
H	3.43965	1.71389	-0.04779	H	4.11778	-1.53214	0.02253	H	4.18988	0.49766	1.12812
H	3.74095	0.83505	-1.54468	H	3.72187	-0.27171	1.18063	H	3.58816	1.53602	-0.15275
H	2.08965	-0.92414	-0.91173	H	2.09730	-0.81875	-1.38899	H	2.14924	-0.95712	0.95638
H	-0.90379	0.67053	-1.42427	H	-0.47216	1.20877	-0.34695	H	-0.52694	-0.62110	-1.16995
H	-0.18186	-0.92023	-1.16989	H	-0.07659	-0.09297	-1.47219	H	-0.03083	-1.33706	0.36585
H	-1.61651	0.64111	1.02611	H	-1.58029	-0.54063	1.14596	H	-1.66006	1.23565	0.16911
H	-2.64364	-1.82315	-0.50069	H	-2.73478	-0.56144	-1.70395	H	-2.63962	-1.57651	0.94998
H	-5.93279	-0.99570	-0.06847	H	-5.86236	0.36705	-0.67937	H	-5.87055	-0.95984	0.02911
H	-5.50803	-0.33249	-1.64009	H	-5.07297	1.87333	-1.12416	H	-5.13594	-1.68974	-1.39140

H	-4.85510	-1.90695	-1.14515
H	0.62777	2.71223	-0.96248
H	1.61371	2.62134	0.51298
H	-0.14283	2.45603	0.60867
H	-5.07033	1.17437	1.02442
H	-4.51426	1.84357	-0.50211
H	-3.37619	1.65787	0.83917
H	0.03170	-1.07895	1.44082

H	-4.78846	0.41296	-2.09199
H	1.23108	1.68675	1.52801
H	1.99245	0.27452	2.29219
H	0.23469	0.45042	2.30668
H	-4.84470	0.59102	1.67647
H	-3.92856	2.03171	1.26117
H	-3.08699	0.61120	1.89568
H	-0.33501	-2.25792	-0.01685

H	-4.70661	-2.28602	0.22435
H	1.05614	1.11609	-2.20653
H	1.81876	2.27363	-1.09564
H	0.05791	2.16282	-1.18981
H	-5.03219	1.33099	-0.78903
H	-4.15836	0.65594	-2.15577
H	-3.29762	1.64196	-0.96535
H	-0.30407	0.62658	2.07604

**Conformer 7**			
Relative energy (kcal/mol)			1.21
Population (%)			3.9
C	-4.30127	-0.52091	0.80209
H	-5.26601	-0.10039	1.11094
H	-3.71246	-0.64001	1.72100
C	-3.61044	0.50474	-0.11600
C	-2.20690	0.11206	-0.51803
C	-0.96661	0.49856	0.19142
C	0.26954	-0.38651	0.04152
C	1.48731	0.35054	-0.56524
C	2.71578	-0.51899	-0.54191
C	3.79589	-0.42079	0.24878
C	4.92584	-1.41537	0.13284
C	-0.98204	1.38969	1.41420
C	4.00533	0.63681	1.30436
O	1.23105	0.67599	-1.93698
O	-1.36557	1.16581	-1.05194
C	-4.53800	-1.89382	0.16348
H	-5.09936	-2.54647	0.84086
H	-5.11512	-1.80512	-0.76465
H	-3.59965	-2.40510	-0.07701
H	-4.20179	0.62825	-1.03375
H	-3.59143	1.48309	0.37304
H	-2.14549	-0.80793	-1.10187
H	0.53771	-0.78077	1.02802
H	0.04598	-1.24412	-0.60315
H	1.66110	1.27255	0.00879
H	2.68027	-1.33090	-1.26930
H	5.87017	-0.90912	-0.10848
H	5.09091	-1.93857	1.08452
H	4.73451	-2.16598	-0.63930
H	-1.03342	0.78417	2.32582
H	-1.82707	2.07990	1.40497
H	-0.06382	1.98505	1.46342
H	4.96296	1.15016	1.14755
H	4.06078	0.18014	2.30174
H	3.21739	1.39209	1.32773
H	0.30346	0.96861	-1.97457

**Conformer 8**			
Relative energy (kcal/mol)			1.28
Population (%)			3.4
C	3.85034	-1.25156	0.17695
H	3.41980	-2.08903	-0.38850
H	3.57645	-1.41546	1.22685
C	3.21338	0.06298	-0.30049
C	1.70755	0.04204	-0.17585
C	0.84993	1.19297	0.17511
C	-0.61585	1.25736	-0.25442
C	-1.33119	-0.07308	-0.51674
C	-2.82492	0.10711	-0.58643
C	-3.76936	-0.43831	0.19845
C	-5.23069	-0.14552	-0.04244
C	1.43071	2.56035	0.47618
C	-3.50564	-1.35647	1.36621
O	-0.85196	-0.57079	-1.78577
O	1.15003	0.17049	1.15932
C	5.37355	-1.26554	0.03266
H	5.79824	-2.21276	0.38278
H	5.67629	-1.13248	-1.01289
H	5.83606	-0.45992	0.61517
H	3.63922	0.89843	0.26416
H	3.46596	0.22983	-1.35742
H	1.22863	-0.74236	-0.76191
H	-1.16111	1.78160	0.54042
H	-0.69793	1.88163	-1.15349
H	-1.05518	-0.77053	0.28301
H	-3.14734	0.75906	-1.39995
H	-5.78994	-1.07117	-0.23407
H	-5.69030	0.31439	0.84270
H	-5.38105	0.52682	-0.89190
H	1.45979	3.17571	-0.43031
H	2.44082	2.49585	0.88337
H	0.80530	3.08072	1.21072
H	-4.07189	-2.29036	1.25442
H	-3.85324	-0.89634	2.30067
H	-2.45223	-1.61172	1.49437
H	-1.31787	-1.40357	-1.95188

**Conformer 9**			
Relative energy (kcal/mol)			1.35
Population (%)			3.0
C	4.18416	-1.08676	-0.03685
H	3.87846	-2.01014	-0.54685
H	3.94625	-1.22693	1.02495
C	3.35695	0.08977	-0.57831
C	1.87247	-0.14134	-0.43268
C	0.85198	0.88778	-0.13955
C	-0.58949	0.63164	-0.57640
C	-1.59595	0.59706	0.60086
C	-3.02122	0.63589	0.09535
C	-3.86185	-0.36350	-0.21986
C	-5.24994	-0.05051	-0.72897
C	1.21280	2.33914	0.08920
C	-3.55830	-1.83844	-0.11970
O	-1.35813	-0.53885	1.43119
O	1.31959	-0.03013	0.90526
C	5.69091	-0.88240	-0.20648
H	6.25401	-1.73429	0.18965
H	5.96146	-0.76779	-1.26290
H	6.03281	0.01543	0.32173
H	3.65414	1.01180	-0.06894
H	3.57275	0.23067	-1.64684
H	1.51264	-1.02665	-0.96323
H	-0.88318	1.41540	-1.28380
H	-0.66723	-0.32509	-1.10418
H	-1.44888	1.51487	1.19682
H	-3.38234	1.65308	-0.06032
H	-5.41498	-0.49645	-1.71923
H	-6.01554	-0.47890	-0.06786
H	-5.43196	1.02554	-0.80525
H	1.22405	2.88239	-0.86238
H	2.18961	2.44510	0.56375
H	0.47088	2.81866	0.73682
H	-3.66605	-2.31252	-1.10506
H	-4.28500	-2.33497	0.53804
H	-2.55902	-2.03010	0.26573
H	-0.38970	-0.62738	1.48808

**Conformer 10**			
Relative energy (kcal/mol)			1.48
Population (%)			2.5
C	-3.96595	-1.27134	-0.28790
H	-3.48947	-2.23018	-0.53345
H	-4.22077	-0.79983	-1.24526
C	-2.95714	-0.37678	0.44913
C	-1.69456	-0.16013	-0.35224
C	-0.89603	1.08394	-0.41089
C	0.58408	0.95036	-0.76158
C	1.50438	0.54283	0.39983
C	2.91606	0.31027	-0.07129
C	3.63383	-0.82498	-0.03418
C	5.05293	-0.85339	-0.54819
C	-1.30672	2.34760	0.31255
C	3.13808	-2.15062	0.48888
O	1.48808	1.61563	1.36104
O	-1.77535	0.70495	-1.50672
C	-5.24095	-1.52536	0.51895
H	-5.94078	-2.16253	-0.03269
H	-5.01855	-2.02299	1.47053
H	-5.75902	-0.58717	0.75034
H	-3.42790	0.58175	0.68954
H	-2.68026	-0.84462	1.40468
H	-1.14977	-1.08723	-0.55817
H	0.67292	0.18881	-1.54279

**Conformer 11**			
Relative energy (kcal/mol)			1.51
Population (%)			2.3
C	3.86983	-0.90121	-0.28135
H	3.67334	-0.51595	-1.29040
H	3.43330	-1.90835	-0.24964
C	3.16225	-0.00798	0.74918
C	1.66134	0.00749	0.56462
C	0.90040	1.00846	-0.21360
C	-0.45993	0.67967	-0.82868
C	-1.28520	-0.42711	-0.16120
C	-2.71501	-0.41217	-0.63510
C	-3.83034	-0.19630	0.08236
C	-5.18770	-0.23480	-0.57742
C	1.58418	2.17582	-0.89700
C	-3.86751	0.10770	1.55967
O	-0.67743	-1.68720	-0.52131
O	0.91682	1.05509	1.23629
C	5.38038	-0.99278	-0.05264
H	5.85802	-1.63456	-0.80096
H	5.85295	-0.00522	-0.11220
H	5.60832	-1.40860	0.93592
H	3.37473	-0.38186	1.76080
H	3.56558	1.00894	0.70705
H	1.19511	-0.97353	0.65370
H	-1.05478	1.60145	-0.80721

**Conformer 12**			
Relative energy (kcal/mol)			1.55
Population (%)			2.2
C	4.06338	-0.87611	-0.78071
H	3.71739	-0.32432	-1.66442
H	3.73314	-1.91429	-0.91901
C	3.39783	-0.29379	0.47579
C	1.89222	-0.40662	0.43140
C	0.95422	0.64697	-0.01599
C	-0.41804	0.22998	-0.54186
C	-1.59640	0.79983	0.28725
C	-2.90837	0.61365	-0.44150
C	-3.82741	-0.36299	-0.36183
C	-5.06224	-0.30781	-1.23177
C	1.42847	2.01634	-0.44975
C	-3.76301	-1.57077	0.54084
O	-1.61980	0.21797	1.58925
O	1.14593	0.35856	1.41004
C	5.59172	-0.83215	-0.71490
H	6.04017	-1.25293	-1.62122
H	5.95632	0.19642	-0.61042
H	5.96908	-1.40515	0.14010
H	3.75138	-0.83822	1.36219
H	3.69775	0.74979	0.61340
H	1.53164	-1.43342	0.33011
H	-0.50798	0.56284	-1.58207

H	0.95007	1.89439	-1.18560
H	1.09482	-0.36623	0.86088
H	3.38937	1.20485	-0.47887
H	5.74958	-1.16696	0.24085
H	5.16100	-1.58246	-1.36239
H	5.37514	0.12302	-0.92082
H	-0.80766	2.41063	1.28246
H	-2.38791	2.40043	0.45301
H	-1.00089	3.22198	-0.27383
H	3.81632	-2.53520	1.26172
H	3.13076	-2.89974	-0.31391
H	2.13254	-2.10744	0.91171
H	2.10062	1.36774	2.06909

H	-0.32131	0.42217	-1.88662
H	-1.22859	-0.28569	0.92465
H	-2.82190	-0.61842	-1.70135
H	-5.82887	-0.99546	-0.11196
H	-5.71013	0.72373	-0.45699
H	-5.11968	-0.45304	-1.64699
H	1.79118	1.94077	-1.94723
H	2.52344	2.44468	-0.41101
H	0.93211	3.05681	-0.87713
H	-4.52339	-0.60208	2.08064
H	-4.29340	1.10466	1.73415
H	-2.88682	0.07810	2.03775
H	-1.21046	-2.38233	-0.10787

H	-0.51455	-0.86123	-0.54207
H	-1.44415	1.88955	0.38303
H	-3.09353	1.40610	-1.16774
H	-5.13596	-1.20159	-1.86618
H	-5.97335	-0.29086	-0.61830
H	-5.07448	0.57272	-1.88089
H	1.61650	2.02882	-1.52911
H	2.34178	2.31436	0.06770
H	0.66196	2.76978	-0.23822
H	-3.80466	-2.49054	-0.05882
H	-4.63841	-1.60014	1.20427
H	-2.86477	-1.58142	1.15451
H	-0.68850	0.15223	1.86697

**Conformer 13**			
Relative energy (kcal/mol)			1.62
Population (%)			1.9
C	3.01868	1.54004	0.31627
H	2.50870	1.24296	1.24200
H	2.39184	2.31613	-0.14367
C	3.10202	0.32955	-0.62578
C	1.73748	-0.19785	-1.00820
C	1.01212	-1.29954	-0.33604
C	-0.51168	-1.30104	-0.43358
C	-1.23692	-0.35295	0.53461
C	-2.72248	-0.34082	0.28476
C	-3.49403	0.67873	-0.12793
C	-4.98067	0.49085	-0.31108
C	1.61564	-2.09271	0.80197
C	-2.99986	2.06604	-0.45611
O	-0.97702	-0.81939	1.87246
O	1.66448	-1.50104	-1.62149
C	4.39079	2.12885	0.65241
H	4.30111	2.98822	1.32574
H	5.03200	1.38769	1.14372
H	4.91031	2.46752	-0.25169
H	3.62652	0.61909	-1.54724
H	3.70026	-0.46418	-0.16704
H	1.11292	0.54608	-1.51321
H	-0.77972	-1.00687	-1.45329
H	-0.89116	-2.31953	-0.27974
H	-0.81422	0.65338	0.41171
H	-3.20181	-1.30068	0.48313
H	-5.54540	1.18897	0.32134
H	-5.27859	0.70292	-1.34665
H	-5.29955	-0.52637	-0.06694
H	1.26669	-1.70418	1.76158
H	2.70690	-2.07628	0.77594
H	1.29389	-3.13850	0.73113
H	-3.55147	2.81695	0.12449
H	-3.18595	2.29750	-1.51323
H	-1.93397	2.20850	-0.26828
H	-1.45562	-0.23075	2.47457

**Conformer 14**			
Relative energy (kcal/mol)			1.90
Population (%)			1.2
C	3.10270	1.87461	-0.26559
H	2.41443	2.46651	-0.88493
H	4.08135	2.36194	-0.35472
C	3.21336	0.45582	-0.85143
C	1.87118	-0.21277	-1.05901
C	1.24239	-1.23082	-0.18825
C	-0.28278	-1.33828	-0.20794
C	-1.02303	-0.15521	0.43458
C	-2.51323	-0.37086	0.44320
C	-3.46498	0.33627	-0.18894
C	-4.92037	-0.03866	-0.04764
C	1.92645	-1.79684	1.03631
C	-3.21570	1.52518	-1.08373
O	-0.54121	-0.03005	1.78788
O	1.87112	-1.60220	-1.44679
C	2.64881	1.92306	1.19731
H	2.59581	2.95781	1.55472
H	1.66247	1.46956	1.34146
H	3.35351	1.38791	1.84486
H	3.70353	0.51105	-1.83292
H	3.85732	-0.16478	-0.21963
H	1.18252	0.39336	-1.65710
H	-0.60499	-1.40443	-1.25203
H	-0.59318	-2.26682	0.28767
H	-0.76509	0.75346	-0.12392
H	-2.82448	-1.21608	1.05901
H	-5.50303	0.79772	0.36117
H	-5.36182	-0.27299	-1.02554
H	-5.06030	-0.90480	0.60522
H	1.54902	-1.31133	1.93930
H	3.01120	-1.68289	0.99074
H	1.70531	-2.86829	1.11281
H	-3.80276	2.38882	-0.74520
H	-3.54891	1.30933	-2.10746
H	-2.16759	1.82645	-1.13264
H	-1.03235	0.69756	2.19713

**Conformer 15**			
Relative energy (kcal/mol)			1.91
Population (%)			1.2
C	3.68600	0.48140	-0.59883
H	3.47563	0.78054	-1.63456
H	4.11051	-0.52904	-0.65023
C	2.36867	0.42334	0.19007
C	1.36433	-0.51015	-0.44423
C	0.40780	-1.37950	0.27287
C	-0.86386	-1.80968	-0.44404
C	-2.11100	-0.96393	-0.14374
C	-2.00146	0.44680	-0.65853
C	-2.17711	1.59912	0.01136
C	-2.05643	2.92620	-0.69830
C	0.36966	-1.45453	1.78531
C	-2.49877	1.71220	1.48148
O	-3.18857	-1.64833	-0.81427
O	1.60753	-1.93121	-0.33190
C	4.70823	1.44100	0.01378
H	5.63606	1.45877	-0.56830
H	4.32059	2.46612	0.05162
H	4.96566	1.14738	1.03834
H	2.57466	0.12238	1.22232
H	1.92050	1.42620	0.23315
H	1.07212	-0.21126	-1.45490
H	-0.68626	-1.79957	-1.52501
H	-1.09152	-2.84661	-0.16563
H	-2.29573	-0.96879	0.93819
H	-1.78303	0.50482	-1.72605
H	-2.99080	3.49810	-0.62076
H	-1.27624	3.54765	-0.23875
H	-1.81614	2.80630	-1.75849
H	-0.28742	-0.68550	2.20511
H	1.36458	-1.32850	2.21552
H	-0.01280	-2.43125	2.10347
H	-3.40198	2.31886	1.62754
H	-1.69111	2.23221	2.01333
H	-2.65790	0.74981	1.97147
H	-3.97740	-1.09593	-0.71125

**Conformer 16**			
Relative energy (kcal/mol)			1.93
Population (%)			1.1
C	-4.00745	-1.10648	0.62812
H	-5.05705	-0.94768	0.90650
H	-3.55133	-1.65716	1.46222
C	-3.32367	0.26658	0.50055
C	-1.82636	0.17060	0.30648
C	-0.97732	1.19309	-0.33895
C	0.50565	1.33561	0.00352
C	1.22649	0.09409	0.54179
C	2.72238	0.26724	0.51052
C	3.63240	-0.45427	-0.16528
C	5.10310	-0.13128	-0.05652
C	-1.56602	2.46058	-0.92664
C	3.31763	-1.61157	-1.08062
O	0.79653	-0.08791	1.90915
O	-1.32362	-0.02707	-1.04204
C	-3.94390	-1.95921	-0.64289
H	-4.46617	-2.91226	-0.50230
H	-4.41428	-1.44401	-1.48879

H	-2.91133	-2.18166	-0.92920
H	-3.77900	0.82715	-0.32234
H	-3.50532	0.84167	1.41926
H	-1.32713	-0.45875	1.04338
H	1.01883	1.65131	-0.91354
H	0.62896	2.15178	0.72700
H	0.91717	-0.76749	-0.06177
H	3.07947	1.08426	1.13963
H	5.66861	-0.99661	0.31455
H	5.52514	0.11324	-1.04055
H	5.29079	0.71269	0.61336
H	-1.54226	3.27312	-0.19145
H	-2.59758	2.32045	-1.25305
H	-0.97869	2.78144	-1.79478
H	3.88347	-2.50297	-0.77973
H	3.62916	-1.38221	-2.10829
H	2.25855	-1.87422	-1.10630
H	1.26913	-0.86219	2.24891

Table S2. Cartesian coordinates, energies, and population at 298 K of significantly populated conformers of model compound **1s**. Conformers were optimized at the B3LYP/6-31G(d,p)/SMD($CHCl_3$) level.

Conformer 1				Conformer 2				Conformer 3			
Relative energy (kcal/mol)			0.00	Relative energy (kcal/mol)			0.19	Relative energy (kcal/mol)			0.64
Population (%)			28.1	Population (%)			20.5	Population (%)			9.6
C	-3.84388	-1.18961	0.38176	C	-3.41311	1.48974	0.12514	C	-3.83788	-1.45933	-0.58469
H	-3.30954	-2.13320	0.55567	H	-3.07686	1.46825	1.16994	H	-4.73521	-1.56628	-1.20682
H	-3.88088	-0.67282	1.34888	H	-2.80646	2.25551	-0.37657	H	-3.23207	-2.35731	-0.76774
C	-3.04550	-0.32823	-0.60930	C	-3.14518	0.12249	-0.52290	C	-3.06135	-0.22143	-1.06731
C	-1.63725	-0.06783	-0.13017	C	-1.67517	-0.22870	-0.53136	C	-1.70893	-0.06857	-0.40858
C	-0.85416	1.16723	-0.34745	C	-0.97118	-1.03736	0.48810	C	-0.96241	1.19797	-0.25406
C	0.67432	1.13724	-0.36064	C	0.52535	-0.85430	0.74115	C	0.56379	1.20620	-0.16587
C	1.37162	0.09134	0.53628	C	1.40629	-0.47309	-0.46883	C	1.24058	-0.00150	0.51835
C	2.86823	0.21630	0.42158	C	2.84780	-0.34554	-0.05119	C	2.73609	0.17557	0.53466
C	3.73335	-0.60611	-0.19189	C	3.57882	0.76883	0.10616	C	3.66562	-0.47466	-0.18249
C	5.20995	-0.29180	-0.21073	C	5.01867	0.68625	0.55263	C	5.12867	-0.13342	-0.03188
C	-1.43468	2.35749	-1.08073	C	-1.70627	-1.65203	1.65970	C	-1.54316	2.51896	-0.71192
C	3.36068	-1.87920	-0.91087	C	3.07929	2.17411	-0.12261	C	3.38380	-1.56687	-1.18465
O	1.03922	0.29742	1.91321	O	1.35270	-1.48784	-1.47698	O	0.81877	-0.11612	1.88155
O	-1.47355	0.89383	0.95081	O	-1.32145	-1.60967	-0.81333	O	-1.65296	0.59456	0.88651
C	-5.26540	-1.48695	-0.09959	C	-4.88858	1.89227	0.06951	C	-4.24665	-1.40910	0.89063
H	-5.81051	-2.09881	0.62717	H	-5.05138	2.86864	0.53833	H	-4.82535	-2.29776	1.16582
H	-5.25867	-2.03004	-1.05217	H	-5.51986	1.16359	0.59130	H	-4.86726	-0.52994	1.09992
H	-5.83638	-0.56312	-0.24956	H	-5.24407	1.95661	-0.96548	H	-3.37604	-1.36034	1.55211
H	-3.57455	0.61363	-0.78261	H	-3.49412	0.13734	-1.56449	H	-3.66416	0.67744	-0.90528
H	-2.98279	-0.84370	-1.57811	H	-3.72201	-0.65601	-0.01442	H	-2.89312	-0.30334	-2.15004
H	-1.06805	-0.98383	0.03821	H	-1.06862	0.48482	-1.09181	H	-1.11494	-0.98408	-0.43567
H	1.03301	2.13044	-0.05994	H	0.91593	-1.79198	1.15783	H	0.86581	2.11156	0.37690
H	0.99812	0.98931	-1.39844	H	0.64256	-0.09266	1.52213	H	0.95822	1.30676	-1.18479
H	1.05655	-0.91339	0.21942	H	1.05164	0.48332	-0.87906	H	0.97898	-0.91540	-0.03406
H	3.26139	1.10963	0.90823	H	3.32180	-1.30736	0.14768	H	3.06758	0.94801	1.22961
H	5.58159	-0.20263	-1.24056	H	5.17468	1.24035	1.48819	H	5.55939	0.18921	-0.98950
H	5.78838	-1.09974	0.25710	H	5.68616	1.14225	-0.19087	H	5.70542	-1.01267	0.28509
H	5.43837	0.63961	0.31515	H	5.34066	-0.34685	0.71149	H	5.29134	0.66310	0.69975
H	-1.17041	2.31643	-2.14334	H	-1.62078	-1.00906	2.54280	H	-1.20362	2.75265	-1.72723
H	-2.52118	2.40518	-0.99542	H	-2.76464	-1.81134	1.44776	H	-2.63402	2.51489	-0.70386
H	-1.02149	3.28534	-0.67046	H	-1.26153	-2.62137	1.91029	H	-1.20152	3.32486	-0.05323
H	3.59762	-1.80146	-1.98053	H	3.10087	2.74777	0.81376	H	3.68654	-1.25258	-2.19259
H	3.94872	-2.72243	-0.52587	H	3.73635	2.70494	-0.82380	H	3.97478	-2.46200	-0.95103
H	2.30339	-2.13679	-0.82226	H	2.06136	2.21699	-0.51513	H	2.33274	-1.85890	-1.22886
H	0.07831	0.46327	1.92790	H	0.40788	-1.70970	-1.57051	H	-0.14897	0.00166	1.86381

Conformer 4				Conformer 5				Conformer 6			
Relative energy (kcal/mol)			0.91	Relative energy (kcal/mol)			0.91	Relative energy (kcal/mol)			1.07
Population (%)			6.1	Population (%)			6.0	Population (%)			4.6
C	4.07624	0.98581	0.04407	C	-3.49418	1.63976	-0.64192	C	-4.43800	-0.66554	0.29755
H	4.15252	0.52781	1.03820	H	-2.89448	2.14845	-1.40880	H	-4.25141	-1.70311	0.60597
H	5.08509	0.95839	-0.38567	H	-4.54193	1.84909	-0.88855	H	-4.57667	-0.08973	1.22134
C	3.16112	0.10953	-0.83233	C	-3.26948	0.12164	-0.75018	C	-3.20308	-0.12639	-0.44068
C	1.76991	-0.05441	-0.26504	C	-1.81390	-0.27156	-0.62029	C	-1.94852	-0.22293	0.39467
C	0.97154	-1.29979	-0.26571	C	-1.17116	-0.92729	0.54139	C	-0.82989	0.73797	0.39588
C	-0.55620	-1.25277	-0.22780	C	0.32496	-0.76037	0.80880	C	0.54655	0.24105	0.81967
C	-1.21152	-0.07795	0.53036	C	1.25154	-0.60510	-0.41702	C	1.47873	-0.12786	-0.34290
C	-2.71231	-0.19867	0.49070	C	2.68641	-0.47253	0.02197	C	2.77315	-0.71620	0.15261
C	-3.59328	0.53214	-0.20999	C	3.45888	0.62473	0.03993	C	4.02092	-0.24060	0.00333
C	-5.07235	0.24147	-0.12539	C	4.88246	0.55437	0.53774	C	5.20077	-0.98836	0.57572
C	1.51533	-2.59441	-0.83098	C	-1.95930	-1.31806	1.77277	C	-0.84471	1.99027	-0.45499
C	-3.23672	1.67353	-1.13015	C	3.02348	1.99965	-0.40361	C	4.38456	1.04016	-0.70646
O	-0.82856	-0.08444	1.90962	O	1.17898	-1.75962	-1.25991	O	0.77070	-1.09306	-1.14388
O	1.64258	-0.84373	0.95270	O	-1.51244	-1.69310	-0.65722	O	-1.79346	0.71921	1.48378
C	3.62933	2.44449	0.18140	C	-3.17068	2.22939	0.73402	C	-5.71314	-0.60392	-0.54593
H	4.35700	3.02058	0.76320	H	-3.38147	3.30408	0.75903	H	-6.57560	-0.99275	0.00646
H	2.66411	2.53359	0.69138	H	-2.11507	2.09695	0.99577	H	-5.61283	-1.19545	-1.46385
H	3.53407	2.92637	-0.79924	H	-3.77049	1.75629	1.52024	H	-5.94594	0.42614	-0.84099
H	3.63285	-0.86672	-0.97242	H	-3.60867	-0.22012	-1.73699	H	-3.38348	0.90998	-0.74424
H	3.06251	0.56198	-1.82916	H	-3.88325	-0.40378	-0.01197	H	-3.04180	-0.70557	-1.36094
H	1.20798	0.87822	-0.21448	H	-1.16061	0.31137	-1.27263	H	-1.69390	-1.24707	0.67999
H	-0.91086	-2.18640	0.22827	H	0.66642	-1.63474	1.37830	H	0.42068	-0.63209	1.46947
H	-0.91758	-1.25455	-1.26374	H	0.45353	0.10905	1.46567	H	1.03732	1.02048	1.41616
H	-0.90189	0.86557	0.05818	H	0.95021	0.29009	-0.97981	H	1.67040	0.77251	-0.94116
H	-3.09373	-1.00093	1.12344	H	3.11449	-1.41258	0.37184	H	2.64236	-1.65512	0.69288
H	-5.48460	-0.00005	-1.11449	H	5.03787	1.23345	1.38705	H	5.75044	-0.36396	1.29275
H	-5.62435	1.12099	0.23237	H	5.58651	0.87137	-0.24335	H	5.91676	-1.25465	-0.21337

H	-5.28910	-0.59369	0.54672
H	1.23187	-2.70133	-1.88403
H	2.60205	-2.65244	-0.75543
H	1.09345	-3.44475	-0.28430
H	-3.52115	1.43799	-2.16452
H	-3.79674	2.57676	-0.85500
H	-2.17342	1.92110	-1.12603
H	0.13130	-0.25699	1.91324

H	5.15729	-0.45563	0.85484
H	-1.88207	-0.54076	2.54085
H	-3.01456	-1.48554	1.55203
H	-1.55171	-2.24354	2.19419
H	3.04821	2.70212	0.44034
H	3.71744	2.39764	-1.15543
H	2.01713	2.02389	-0.82638
H	0.22886	-1.96331	-1.34061

H	4.89743	-1.90637	1.08696
H	-0.45276	1.78661	-1.45722
H	-1.85475	2.39083	-0.55660
H	-0.21978	2.76728	0.00054
H	4.84070	1.75060	-0.00438
H	5.13770	0.84766	-1.48158
H	3.53452	1.53801	-1.17690
H	1.37867	-1.37929	-1.84133

**Conformer 7**			
Relative energy (kcal/mol)			1.17
Population (%)			3.9
C	3.47230	-1.26571	-0.04568
H	2.70359	-1.56167	0.67910
H	3.22905	-1.79537	-0.97638
C	3.38386	0.24918	-0.28766
C	2.02619	0.66368	-0.80657
C	0.91015	1.17670	0.01126
C	-0.50925	0.98842	-0.50835
C	-1.24589	-0.22337	0.08031
C	-2.59061	-0.42261	-0.56699
C	-3.80855	-0.35493	-0.00360
C	-5.05003	-0.59469	-0.82808
C	1.04125	1.40125	1.50271
C	-4.07916	-0.03521	1.44589
O	-0.40672	-1.36939	-0.16031
O	1.70198	2.07255	-0.81548
C	4.85074	-1.71006	0.44858
H	4.88607	-2.79322	0.60877
H	5.10925	-1.22585	1.39777
H	5.63418	-1.45582	-0.27520
H	4.14086	0.54581	-1.02742
H	3.62313	0.79217	0.63261
H	1.73949	0.15340	-1.72978
H	-0.47472	0.89270	-1.59930
H	-1.09583	1.88744	-0.28065
H	-1.35988	-0.07595	1.16212
H	-2.52824	-0.65748	-1.63072
H	-5.71012	0.28282	-0.80797
H	-5.63561	-1.43028	-0.42177
H	-4.81326	-0.81819	-1.87212
H	0.77230	0.49836	2.06113
H	2.05912	1.68377	1.77697
H	0.37198	2.20772	1.82473
H	-4.65068	0.89847	1.53126
H	-4.69957	-0.81803	1.90131
H	-3.17479	0.07324	2.04767
H	-0.89441	-2.14522	0.15319

**Conformer 8**			
Relative energy (kcal/mol)			1.19
Population (%)			3.8
C	-3.60021	1.43017	0.40579
H	-4.68780	1.56335	0.45419
H	-3.26702	1.26215	1.43825
C	-3.31386	0.17028	-0.43233
C	-1.84857	-0.20162	-0.48924
C	-1.15275	-1.09977	0.45895
C	0.35134	-0.97316	0.70266
C	1.22685	-0.53170	-0.49055
C	2.67607	-0.46907	-0.08518
C	3.43875	0.61379	0.13114
C	4.88082	0.46595	0.55300
C	-1.88655	-1.78112	1.59432
C	2.97532	2.04329	-0.00468
O	1.13400	-1.47234	-1.56535
O	-1.53379	-1.56853	-0.87444
C	-2.96421	2.71525	-0.13510
H	-3.26433	3.58096	0.46518
H	-3.27382	2.90623	-1.16951
H	-1.86979	2.67233	-0.11927
H	-3.65978	0.33193	-1.46246
H	-3.89141	-0.67232	-0.04186
H	-1.22931	0.52923	-1.00893
H	0.72193	-1.94718	1.04831
H	0.49854	-0.27061	1.53242
H	0.89220	0.45963	-0.82854
H	3.12635	-1.45348	0.04736
H	5.06265	0.95864	1.51778
H	5.55201	0.94760	-0.17072
H	5.17593	-0.58310	0.64571
H	-1.75031	-1.22115	2.52614
H	-2.95595	-1.87702	1.40085
H	-1.47867	-2.78616	1.74877
H	3.02195	2.55722	0.96486
H	3.63959	2.59883	-0.67943
H	1.95520	2.13764	-0.38231
H	0.18226	-1.66156	-1.66117

**Conformer 9**			
Relative energy (kcal/mol)			1.25
Population (%)			3.4
C	-3.82344	1.27579	-0.33518
H	-3.63242	1.60363	-1.36614
H	-4.88529	1.47608	-0.14538
C	-3.59593	-0.24255	-0.23995
C	-2.22700	-0.68378	-0.71372
C	-1.09484	-1.12346	0.12962
C	0.31667	-1.03523	-0.43999
C	1.11598	0.20817	-0.02872
C	2.48695	0.21494	-0.65133
C	3.68310	0.19618	-0.03952
C	4.95834	0.22005	-0.84696
C	-1.19101	-1.19653	1.63847
C	3.89608	0.14125	1.45312
O	0.36603	1.34975	-0.49017
O	-1.91481	-2.08920	-0.57995
C	-2.96838	2.10410	0.62898
H	-3.19913	3.17167	0.53623
H	-1.89974	1.97407	0.42974
H	-3.15758	1.81700	1.67060
H	-4.33462	-0.75377	-0.87242
H	-3.77803	-0.58639	0.78386
H	-1.95621	-0.27656	-1.69189
H	0.25803	-1.07302	-1.53350
H	0.87751	-1.92117	-0.11631
H	1.19105	0.23850	1.06569
H	2.46768	0.25093	-1.74161
H	5.57089	-0.66745	-0.63916
H	5.57469	1.08925	-0.58081
H	4.76364	0.25500	-1.92258
H	-0.86650	-0.25950	2.10270
H	-2.21109	-1.40557	1.96531
H	-0.54707	-1.99895	2.01744
H	4.42531	-0.78004	1.73013
H	4.53477	0.97240	1.77938
H	2.97043	0.17854	2.03058
H	0.88474	2.13745	-0.26985

**Conformer 10**			
Relative energy (kcal/mol)			1.32
Population (%)			3.0
C	-4.21456	0.17773	-0.58539
H	-4.37773	1.25663	-0.46088
H	-3.75893	0.04638	-1.57476
C	-3.22559	-0.31302	0.48361
C	-1.91723	0.43833	0.43882
C	-0.57429	-0.12144	0.70738
C	0.53065	0.82203	1.19570
C	1.58457	1.20244	0.12572
C	2.26751	0.01683	-0.51648
C	3.52047	-0.42711	-0.32386
C	4.04069	-1.62312	-1.08487
C	-0.37573	-1.58516	1.03727
C	4.51565	0.17958	0.63441
O	0.98867	2.03716	-0.87682
O	-1.02408	0.13980	-0.66613
C	-5.55774	-0.55306	-0.53375
H	-6.24023	-0.18452	-1.30717
H	-6.04987	-0.41513	0.43637
H	-5.43082	-1.63082	-0.68988
H	-3.05658	-1.38732	0.36006
H	-3.66239	-0.17091	1.48221
H	-2.02260	1.51280	0.61046
H	1.03757	0.35688	2.04848

**Conformer 11**			
Relative energy (kcal/mol)			1.39
Population (%)			2.7
C	-4.14845	-1.26662	0.07559
H	-3.87069	-2.15065	-0.51427
H	-3.80402	-1.45837	1.09949
C	-3.40713	-0.03667	-0.47178
C	-1.90895	-0.22997	-0.47843
C	-0.88779	0.80334	-0.19098
C	0.50306	0.60443	-0.79096
C	1.66044	0.69582	0.21698
C	2.96425	0.25544	-0.39595
C	3.73296	-0.79465	-0.06167
C	5.02522	-1.06463	-0.79405
C	-1.27152	2.22205	0.17467
C	3.41544	-1.79241	1.02464
O	1.76800	2.07248	0.63100
O	-1.22851	-0.20644	0.79737
C	-5.66951	-1.10111	0.06432
H	-6.16965	-1.99059	0.46275
H	-6.04546	-0.93658	-0.95260
H	-5.98105	-0.24475	0.67403
H	-3.67923	0.84408	0.11829
H	-3.73106	0.15813	-1.50415
H	-1.58330	-1.05759	-1.11613
H	0.67049	1.33709	-1.59060

**Conformer 12**			
Relative energy (kcal/mol)			1.42
Population (%)			2.6
C	-4.07199	-1.07491	0.03920
H	-3.70617	-1.99977	-0.42658
H	-3.86659	-1.16758	1.11295
C	-3.28098	0.12051	-0.51462
C	-1.79361	-0.03441	-0.30396
C	-0.83555	1.05306	-0.01235
C	0.64194	0.92202	-0.38262
C	1.26382	-0.49227	-0.32207
C	2.68609	-0.46323	-0.83908
C	3.85003	-0.31403	-0.18540
C	5.15159	-0.31118	-0.95369
C	-1.28701	2.48973	0.14182
C	4.01178	-0.13768	1.30468
O	1.18607	-1.03471	0.99361
O	-1.29334	0.15554	1.05080
C	-5.57942	-0.94721	-0.18938
H	-6.11693	-1.81054	0.21733
H	-5.81670	-0.88147	-1.25790
H	-5.98072	-0.04894	0.29444
H	-3.64345	1.04348	-0.05248
H	-3.46018	0.21203	-1.59522
H	-1.38867	-0.93129	-0.77608
H	1.22093	1.56832	0.28899

H	0.09137	1.75987	1.55311	H	0.54916	-0.38936	-1.24764	H	0.77175	1.32926	-1.39337
H	2.32574	1.83504	0.62379	H	1.40105	0.06975	1.07984	H	0.70308	-1.14156	-1.01635
H	1.64593	-0.51396	-1.23839	H	3.30333	0.90314	-1.20618	H	2.73734	-0.55283	-1.92467
H	4.36063	-2.42006	-0.39990	H	5.01133	-2.05791	-1.26250	H	5.80879	-1.12323	-0.61402
H	4.92452	-1.35499	-1.67943	H	5.87692	-1.06324	-0.10054	H	5.70500	0.62259	-0.78506
H	3.28837	-2.03793	-1.76192	H	5.21856	-0.32309	-1.57441	H	4.99909	-0.42707	-2.03084
H	-0.47870	-1.74508	2.11680	H	-1.30555	2.84982	-0.72349	H	-1.20528	3.01941	-0.81393
H	-1.09723	-2.22046	0.52075	H	-2.24700	2.26252	0.66286	H	-2.31722	2.56397	0.49289
H	0.63011	-1.90491	0.74901	H	-0.52263	2.64614	0.84705	H	-0.64543	3.00581	0.86426
H	4.78583	-0.54209	1.41707	H	3.29752	-2.79733	0.59802	H	4.64377	-0.93575	1.71821
H	5.44818	0.42996	0.11188	H	4.24603	-1.85787	1.73965	H	4.53459	0.80517	1.51662
H	4.15318	1.08336	1.12822	H	2.50555	-1.56000	1.58084	H	3.05862	-0.14681	1.82927
H	0.20062	1.54751	-1.17369	H	2.49577	2.11815	1.26847	H	0.28241	-0.83216	1.30128

**Conformer 13**				**Conformer 14**				**Conformer 15**			
Relative energy (kcal/mol)			1.52	Relative energy (kcal/mol)			1.54	Relative energy (kcal/mol)			1.73
Population (%)			2.1	Population (%)			2.1	Population (%)			1.5
C	-4.15445	-0.74998	-0.76335	C	-4.20119	-0.09369	0.59343	C	4.45529	-0.83881	0.52539
H	-3.88620	0.04531	-1.47102	H	-3.75506	-0.66596	1.41721	H	5.20111	-0.70495	1.31927
H	-3.85264	-1.69445	-1.23596	H	-4.34512	0.92700	0.97225	H	4.20171	-1.90783	0.52381
C	-3.36349	-0.55729	0.53932	C	-3.22451	-0.06295	-0.59239	C	3.20103	-0.02629	0.89316
C	-1.86920	-0.63838	0.32567	C	-1.91464	0.60090	-0.23856	C	2.02946	-0.28131	-0.02789
C	-0.95467	0.50677	0.10460	C	-0.67283	-0.08748	0.18140	C	0.92129	0.65524	-0.29288
C	0.34631	0.23652	-0.64924	C	0.33274	0.67504	1.05080	C	-0.42293	0.08206	-0.72394
C	1.61914	0.70889	0.07166	C	1.59951	1.16732	0.30703	C	-1.45199	-0.05241	0.40727
C	2.86321	0.21977	-0.62318	C	2.36670	0.07302	-0.39909	C	-2.70896	-0.73408	-0.06475
C	3.80350	-0.62138	-0.16098	C	3.53512	-0.48591	-0.04358	C	-3.95317	-0.23337	-0.14555
C	4.99648	-0.98331	-1.01246	C	4.17070	-1.55633	-0.89813	C	-5.09373	-1.08736	-0.64395
C	-1.45697	1.93541	0.09981	C	-0.58927	-1.59677	0.25265	C	0.88057	2.04166	0.31516
C	3.78376	-1.28565	1.19356	C	4.31836	-0.13499	1.19741	C	-4.34788	1.17636	0.21932
O	1.60797	2.15024	0.07303	O	1.25385	2.20839	-0.61686	O	-0.82329	-0.83421	1.44028
O	-1.01482	-0.16921	1.39025	O	-0.82103	0.45108	-1.17725	O	1.96484	0.44224	-1.28108
C	-5.66923	-0.75227	-0.54474	C	-5.55907	-0.69676	0.22665	C	5.07577	-0.45864	-0.82263
H	-6.20730	-0.89015	-1.48886	H	-6.23495	-0.70637	1.08847	H	5.97729	-1.04955	-1.01991
H	-6.00910	0.19212	-0.10369	H	-5.45418	-1.72980	-0.12498	H	5.36117	0.59986	-0.84218
H	-5.97182	-1.56038	0.13158	H	-6.04560	-0.12376	-0.57132	H	4.37828	-0.62423	-1.64942
H	-3.64579	-1.34078	1.25669	H	-3.67535	0.49886	-1.42208	H	3.44623	1.04082	0.90753
H	-3.63033	0.39647	1.00564	H	-3.05030	-1.07779	-0.96379	H	2.88430	-0.29622	1.91011
H	-1.54357	-1.59475	-0.09474	H	-2.02567	1.62834	0.11769	H	1.78768	-1.34217	-0.13643
H	0.30469	0.70503	-1.64063	H	0.63418	0.03226	1.88538	H	-0.25816	-0.90113	-1.17858
H	0.44498	-0.84261	-0.80517	H	-0.14357	1.56245	1.48217	H	-0.85204	0.72926	-1.49937
H	1.57377	0.33825	1.10328	H	2.23838	1.64672	1.05516	H	-1.68175	0.94815	0.79653
H	2.99057	0.63259	-1.62532	H	1.89819	-0.27162	-1.32155	H	-2.55071	-1.77033	-0.36761
H	5.04054	-2.06674	-1.18716	H	4.31253	-2.48528	-0.32930	H	-5.55814	-0.64165	-1.53380
H	5.93445	-0.71432	-0.50831	H	5.16906	-1.24655	-1.23563	H	-5.88665	-1.16314	0.11221
H	4.97515	-0.48265	-1.98463	H	3.56927	-1.78706	-1.78216	H	-4.76815	-2.09931	-0.90096
H	-1.65764	2.26287	-0.92693	H	-0.88719	-1.94377	1.24875	H	0.41527	2.01854	1.30637
H	-2.37329	2.04491	0.68296	H	-1.23039	-2.07556	-0.48963	H	1.88229	2.46245	0.41666
H	-0.69362	2.59793	0.51378	H	0.43940	-1.92773	0.08095	H	0.29436	2.71857	-0.31732
H	3.73427	-2.37709	1.08360	H	4.39270	-1.00416	1.86478	H	-4.72690	1.70769	-0.66371
H	4.71335	-1.07367	1.73793	H	5.34830	0.14148	0.93643	H	-5.16831	1.16819	0.94869
H	2.94513	-0.97746	1.82045	H	3.88371	0.68646	1.77051	H	-3.52975	1.76712	0.63563
H	2.40294	2.43675	0.54619	H	0.52941	1.83776	-1.15207	H	-1.49015	-0.98266	2.12666

Table S3. Cartesian coordinates, energies, and population at 298 K of significantly populated conformers of model compound **2r**. Conformers were optimized at the B3LYP/TZVP/SMD($CHCl_3$) level.

Conformer 1			
Relative energy (kcal/mol)			0.00
Population (%)			27.4
C	-4.57249	-0.29321	0.27959
C	-3.11552	-0.38381	0.61375
C	-2.12487	0.44798	0.26684
C	-0.70639	0.15818	0.70513
C	0.16303	-0.43553	-0.41922
C	1.54507	-0.75380	0.08634
C	2.68342	0.17869	0.11734
C	3.75887	-0.03039	1.15631
C	2.59710	1.58505	-0.42367
C	-2.30002	1.69203	-0.56119
O	-0.38412	-1.66846	-0.89678
O	2.60755	-0.87011	-0.87706
H	-5.17101	-0.20593	1.19255
H	-4.81864	0.55152	-0.36217
H	-4.91048	-1.20576	-0.22202
H	-2.84843	-1.23480	1.23666
H	-0.70991	-0.54641	1.54046
H	-0.22964	1.08060	1.05181
H	0.23220	0.27263	-1.25003
H	1.54472	-1.55495	0.82467
H	3.57653	0.59929	2.03091
H	3.79446	-1.07087	1.48024
H	4.73705	0.23856	0.74884
H	3.56453	1.87790	-0.84032
H	2.35552	2.28987	0.37572
H	1.84986	1.67938	-1.20904
H	-1.62870	1.68931	-1.42511
H	-3.31526	1.82118	-0.93126
H	-2.04179	2.57854	0.02783
H	-1.33759	-1.54262	-1.00638

Conformer 2			
Relative energy (kcal/mol)			0.02
Population (%)			26.5
C	-4.68918	-0.15300	0.37589
C	-3.23665	-0.33913	0.68966
C	-2.18839	0.35290	0.23248
C	-0.79150	0.00315	0.69680
C	0.18160	-0.40142	-0.41248
C	1.54862	-0.70013	0.15284
C	2.70287	0.21343	0.16907
C	3.76985	0.00440	1.21639
C	2.64459	1.61050	-0.39775
C	-2.28354	1.51106	-0.72365
O	-0.33387	-1.57901	-1.04612
O	2.61695	-0.85696	-0.80525
H	-5.26556	0.01400	1.29202
H	-4.88483	0.67933	-0.29905
H	-5.10072	-1.05718	-0.08531
H	-3.02653	-1.15619	1.37640
H	-0.83879	-0.80847	1.42710
H	-0.35357	0.87105	1.20467
H	0.26028	0.39864	-1.15254
H	1.53332	-1.48652	0.90716
H	3.59711	0.65752	2.07547
H	3.78209	-1.02896	1.56386
H	4.75479	0.24371	0.80692
H	3.62439	1.88721	-0.79576
H	2.39094	2.32894	0.38571
H	1.91639	1.70201	-1.20103
H	-1.86715	1.25144	-1.70207
H	-3.30733	1.84408	-0.88327
H	-1.70752	2.36556	-0.35394
H	0.27842	-1.82903	-1.75076

Conformer 3			
Relative energy (kcal/mol)			0.42
Population (%)			13.6
C	-3.27043	-1.58910	-0.52555
C	-2.31019	-0.47681	-0.81488
C	-1.85480	0.47103	0.01176
C	-0.89627	1.52618	-0.49595
C	0.54000	1.41207	0.02552
C	1.27523	0.23239	-0.56532
C	1.68505	-0.99501	0.13794
C	1.91035	-2.24502	-0.67761
C	1.39001	-1.24210	1.59629
C	-2.26350	0.61444	1.45334
O	1.21041	2.61823	-0.37231
O	2.64064	0.06315	-0.12539
H	-2.81365	-2.56018	-0.74380
H	-3.61454	-1.60786	0.50760
H	-4.15259	-1.51409	-1.17020
H	-1.95311	-0.44803	-1.84213
H	-1.25556	2.51696	-0.19896
H	-0.86382	1.51195	-1.58807
H	0.54124	1.34929	1.11626
H	1.16847	0.16350	-1.64753
H	1.01407	-2.87018	-0.67229
H	2.15640	-2.00134	-1.71149
H	2.73065	-2.83118	-0.25516
H	2.19088	-1.84389	2.03402
H	0.45711	-1.79923	1.70850
H	1.31587	-0.31763	2.16537
H	-2.77947	1.56854	1.60630
H	-2.92586	-0.18043	1.79094
H	-1.39392	0.62611	2.11623
H	2.13675	2.53817	-0.10726

Conformer 4			
Relative energy (kcal/mol)			0.52
Population (%)			11.4
C	4.09133	-1.56679	-0.31752
C	2.72587	-1.16685	0.15080
C	2.24553	0.06131	0.37723
C	0.81207	0.22580	0.84218
C	-0.18908	0.65221	-0.23633
C	-1.60277	0.58908	0.29181
C	-2.59273	-0.46253	0.00587
C	-3.71075	-0.68453	0.99566
C	-2.29281	-1.64695	-0.87914
C	3.04072	1.32913	0.22342
O	0.09607	2.00143	-0.63112
O	-2.65127	0.81866	-0.67237
H	4.02955	-2.11669	-1.26281
H	4.76140	-0.72185	-0.47009
H	4.56413	-2.24451	0.40120
H	2.04913	-2.00185	0.32266
H	0.76382	0.96100	1.65307
H	0.45823	-0.72494	1.24837
H	-0.08394	-0.00510	-1.10350
H	-1.73894	1.15598	1.21261
H	-3.46199	-1.50210	1.67698
H	-3.89840	0.21290	1.58573
H	-4.63202	-0.95399	0.47261
H	-3.20832	-1.97288	-1.37962
H	-1.92487	-2.48571	-0.28298
H	-1.55503	-1.41514	-1.64454
H	3.03586	1.89464	1.16118
H	4.07804	1.14793	-0.05261
H	2.59071	1.98004	-0.52918
H	-0.54998	2.25223	-1.30510

Conformer 5			
Relative energy (kcal/mol)			0.98
Population (%)			5.2
C	-4.63708	-0.16041	0.50956
C	-3.16829	-0.28863	0.77217
C	-2.15706	0.37301	0.19958
C	-0.73393	0.09267	0.62601
C	0.19202	-0.41521	-0.49097
C	1.56092	-0.73407	0.06397
C	2.68797	0.20604	0.18800
C	3.72657	-0.04490	1.25462
C	2.60370	1.63672	-0.28468
C	-2.31814	1.42497	-0.86339
O	-0.34251	-1.56317	-1.15748
O	2.66059	-0.79333	-0.85953
H	-5.17537	0.08740	1.43044
H	-4.87911	0.59761	-0.23406
H	-5.04990	-1.11230	0.15872
H	-2.91032	-1.02389	1.53181
H	-0.73428	-0.63962	1.43983
H	-0.28607	1.00992	1.02668
H	0.28589	0.33516	-1.27463
H	1.54673	-1.56872	0.76692
H	3.50237	0.53409	2.15416
H	3.76565	-1.10097	1.52327
H	4.71547	0.25806	0.90024
H	3.58003	1.95902	-0.65637
H	2.33131	2.29755	0.54195
H	1.87953	1.76602	-1.08672
H	-1.91428	1.08123	-1.82056
H	-3.35681	1.70516	-1.02811
H	-1.76445	2.33080	-0.59568
H	-0.62110	-2.20568	-0.49096

Conformer 6			
Relative energy (kcal/mol)			1.08
Population (%)			4.4
C	-4.10809	0.60539	1.05646
C	-2.71653	0.46219	0.51840
C	-2.21027	-0.53334	-0.21411
C	-0.77102	-0.56559	-0.68921
C	0.15389	0.57741	-0.29470
C	1.58743	0.24156	-0.63151
C	2.61321	-0.23564	0.31036
C	3.77470	-1.03078	-0.23512
C	2.33527	-0.45655	1.77672
C	-2.98758	-1.74399	-0.65836
O	-0.23118	1.75002	-1.02752
O	2.58974	1.14780	-0.12602
H	-4.58486	1.50592	0.65449
H	-4.75427	-0.24154	0.83000
H	-4.08929	0.72897	2.14448
H	-2.06467	1.29675	0.75458
H	-0.76357	-0.64497	-1.78338
H	-0.31986	-1.49869	-0.32958
H	0.06828	0.77736	0.77647
H	1.73029	-0.00912	-1.68242
H	3.58611	-2.10259	-0.13434
H	3.94464	-0.80644	-1.28846
H	4.68734	-0.79843	0.31997
H	3.23935	-0.25417	2.35695
H	2.05139	-1.49620	1.95799
H	1.54287	0.18826	2.15081
H	-2.52063	-2.66245	-0.28653
H	-4.02300	-1.73724	-0.32461
H	-2.98918	-1.81846	-1.75104
H	0.36450	2.46658	-0.77063

Conformer 7			
Relative energy (kcal/mol)			1.46
Population (%)			2.3
C	-4.67178	-0.46251	-0.52231
C	-3.26568	-0.74638	-0.09189
C	-2.37088	0.09088	0.44264
C	-0.99489	-0.40838	0.81452
C	0.16854	0.26280	0.05953
C	1.49915	-0.29949	0.55820
C	2.83254	0.01615	0.02599
C	4.02941	-0.18610	0.92300
C	3.04777	0.99413	-1.10117
C	-2.63117	1.54547	0.72919
O	0.03117	0.10477	-1.34711
O	2.15764	-1.23089	-0.32203
H	-5.37421	-1.13744	-0.02199
H	-4.98916	0.55995	-0.32110
H	-4.78802	-0.63966	-1.59705
H	-2.94613	-1.77424	-0.24956
H	-0.93373	-1.48804	0.64983
H	-0.82584	-0.23455	1.88499
H	0.15203	1.33878	0.25721
H	1.46208	-0.63551	1.59257
H	4.30635	0.75376	1.40705
H	3.82228	-0.92628	1.69647
H	4.88847	-0.52693	0.33923
H	3.88199	0.66450	-1.72619
H	3.30994	1.97347	-0.69251
H	2.16411	1.10566	-1.72454
H	-2.04889	2.19037	0.06338
H	-3.67821	1.81905	0.61291
H	-2.33029	1.79736	1.75141
H	0.55872	-0.67064	-1.59303

Conformer 8			
Relative energy (kcal/mol)			1.48
Population (%)			2.3
C	3.16526	2.07535	-0.20659
C	2.15885	1.19517	0.46891
C	1.96829	-0.12167	0.32441
C	0.88617	-0.81291	1.13047
C	-0.35556	-1.31608	0.37938
C	-1.00927	-0.25897	-0.47544
C	-2.13914	0.59753	-0.07909
C	-2.31741	1.92023	-0.78451
C	-2.78445	0.52064	1.28272
C	2.79600	-1.00484	-0.56974
O	0.01578	-2.43146	-0.44480
O	-2.34827	-0.55684	-0.93103
H	3.80487	2.57057	0.53181
H	3.81041	1.54118	-0.90266
H	2.66537	2.87466	-0.76416
H	1.50541	1.71639	1.16578
H	0.52951	-0.13205	1.90687
H	1.31192	-1.68324	1.64148
H	-1.07043	-1.66287	1.13080
H	-0.36584	0.11849	-1.26709
H	-1.86112	2.72760	-0.20643
H	-1.85978	1.90189	-1.77388
H	-3.38031	2.14918	-0.89839
H	-3.84993	0.75023	1.19795
H	-2.34029	1.25726	1.95673
H	-2.68678	-0.46481	1.73350
H	2.18265	-1.45533	-1.35316
H	3.62129	-0.47408	-1.04122
H	3.21463	-1.83784	0.00423
H	-0.78410	-2.75760	-0.87858

Conformer 9			
Relative energy (kcal/mol)			1.52
Population (%)			2.1
C	3.64447	-1.05422	-0.77263
C	2.69460	0.08016	-0.54136
C	1.81978	0.24932	0.45573
C	0.94724	1.49001	0.48599
C	-0.39041	1.37426	-0.25447
C	-1.36899	0.41363	0.37869
C	-1.79031	-0.89589	-0.15245
C	-2.35039	-1.91463	0.81108
C	-1.24068	-1.47939	-1.43020
C	1.64554	-0.70200	1.60874
O	-0.97027	2.68851	-0.23218
O	-2.62763	0.27498	-0.31704
H	4.67792	-0.69271	-0.79859
H	3.58386	-1.83198	-0.01255
H	3.45910	-1.52142	-1.74567
H	2.74115	0.86487	-1.29436
H	1.47713	2.32425	0.02238
H	0.73996	1.78060	1.52070
H	-0.19939	1.08546	-1.29118
H	-1.49474	0.57329	1.44920
H	-1.57886	-2.62822	1.10979
H	-2.74596	-1.43490	1.70671
H	-3.15787	-2.47755	0.33559
H	-2.01460	-2.07526	-1.92136
H	-0.39797	-2.14054	-1.21583
H	-0.90951	-0.71362	-2.12832
H	0.59878	-0.99168	1.73157
H	2.23305	-1.61269	1.50615
H	1.94121	-0.21685	2.54534
H	-1.83521	2.63355	-0.66056

Conformer 10			
Relative energy (kcal/mol)			1.55
Population (%)			2.0
C	-3.84290	-1.57464	-0.17897
C	-2.76739	-0.63121	-0.62205
C	-2.19173	0.36267	0.06446
C	-1.12343	1.21578	-0.58220
C	0.28638	1.10807	0.03721
C	0.90644	-0.26243	-0.22102
C	2.26306	-0.68345	0.15600
C	2.53936	-2.15851	0.31482
C	3.21646	0.22043	0.89588
C	-2.55166	0.73841	1.47781
O	1.10942	2.15181	-0.47659
O	1.95006	-0.30034	-1.21774
H	-3.49529	-2.61142	-0.24095
H	-4.18027	-1.39951	0.84169
H	-4.71454	-1.50413	-0.83817
H	-2.42419	-0.78867	-1.64243
H	-1.40653	2.27043	-0.50339
H	-1.04750	0.98081	-1.64729
H	0.21717	1.26499	1.11753
H	0.17029	-1.05387	-0.33561
H	2.45276	-2.45148	1.36411
H	1.83941	-2.75570	-0.27020
H	3.55479	-2.39326	-0.01485
H	4.24097	0.03518	0.56239
H	3.17437	0.00246	1.96612
H	2.98562	1.27290	0.74950
H	-2.76966	1.80908	1.54364
H	-3.41823	0.19714	1.85294
H	-1.72249	0.54874	2.16674
H	1.66008	1.75386	-1.16881

Conformer 11			
Relative energy (kcal/mol)			1.67
Population (%)			1.6
C	-4.10436	0.61215	1.03471
C	-2.67706	0.40582	0.62553
C	-2.16736	-0.55590	-0.15207
C	-0.68527	-0.66075	-0.46872
C	0.16083	0.59872	-0.28158
C	1.60065	0.32693	-0.63263
C	2.62310	-0.19258	0.28984
C	3.78246	-0.97183	-0.28307
C	2.33876	-0.46921	1.74621
C	-2.97787	-1.65786	-0.77896
O	-0.26366	1.65099	-1.15903
O	2.60277	1.20424	-0.08922
H	-4.45014	1.60754	0.73719
H	-4.78958	-0.11842	0.60783
H	-4.20242	0.56694	2.12452
H	-1.99496	1.14266	1.04144
H	-0.57754	-0.98648	-1.50950
H	-0.25706	-1.46686	0.13941
H	0.10858	0.94605	0.75369
H	1.74114	0.11762	-1.69254
H	3.58676	-2.04601	-0.23308
H	3.96107	-0.70044	-1.32387
H	4.69312	-0.77106	0.28756
H	3.23306	-0.26196	2.34002
H	2.08186	-1.52108	1.89373
H	1.52753	0.14414	2.13351
H	-2.55055	-2.63438	-0.52810
H	-4.02025	-1.65986	-0.46752
H	-2.95080	-1.58027	-1.87092
H	-1.21598	1.76707	-1.04295

Conformer 12			
Relative energy (kcal/mol)			1.93
Population (%)			1.0
C	4.04201	-1.58760	-0.31198
C	2.67033	-1.17996	0.13025
C	2.20016	0.04908	0.37340
C	0.75723	0.22037	0.80256
C	-0.20492	0.73899	-0.28203
C	-1.63451	0.65341	0.20201
C	-2.55204	-0.48180	0.00454
C	-3.66743	-0.69329	0.99971
C	-2.15873	-1.71464	-0.77164
C	3.01774	1.30770	0.26773
O	0.08588	2.07955	-0.69547
O	-2.67922	0.72847	-0.78087
H	3.99876	-2.10130	-1.27843
H	4.73142	-0.75063	-0.41389
H	4.48046	-2.30007	0.39475
H	1.97788	-2.00911	0.26244
H	0.70197	0.90382	1.65959
H	0.37258	-0.74351	1.14481
H	-0.08504	0.14526	-1.18732
H	-1.82288	1.28044	1.07597
H	-3.37547	-1.42802	1.75438
H	-3.92563	0.23799	1.50480
H	-4.56013	-1.07076	0.49400
H	-3.03627	-2.12458	-1.27859
H	-1.77676	-2.48572	-0.09796
H	-1.40228	-1.50542	-1.52544
H	2.97249	1.87006	1.20656
H	4.06573	1.11442	0.04580
H	2.61931	1.96595	-0.50763
H	-0.00854	2.66348	0.06962

Table S4. Cartesian coordinates, energies, and population at 298 K of significantly populated conformers of model compound **2s**. Conformers were optimized at the B3LYP/TZVP/SMD($CHCl_3$) level.

**Conformer 1**				**Conformer 2**				**Conformer 3**			
Relative energy (kcal/mol)			0.00	Relative energy (kcal/mol)			0.02	Relative energy (kcal/mol)			0.42
Population (%)			61.1	Population (%)			26.5	Population (%)			13.6
C	4.58415	-0.61269	0.31820	C	4.73132	-0.38544	0.22510	C	4.74615	-0.36001	0.24394
C	3.19410	-0.67119	-0.23658	C	3.31487	-0.69021	-0.15434	C	3.33343	-0.68723	-0.13081
C	2.29517	0.31613	-0.34466	C	2.30174	0.16143	-0.34635	C	2.31171	0.14867	-0.34176
C	0.92902	0.03228	-0.92754	C	0.93745	-0.35960	-0.73611	C	0.95472	-0.39628	-0.72827
C	-0.16607	-0.09841	0.14508	C	-0.18676	-0.04894	0.26133	C	-0.17987	-0.07639	0.24726
C	-1.50541	-0.38732	-0.48760	C	-1.50957	-0.58580	-0.24214	C	-1.50405	-0.57354	-0.29046
C	-2.81045	0.02770	0.04943	C	-2.82294	0.04216	-0.03308	C	-2.81648	0.04747	-0.03581
C	-4.02956	-0.78023	-0.32227	C	-4.05080	-0.83531	-0.03865	C	-4.05005	-0.81939	-0.10619
C	-2.94781	0.83259	1.31649	C	-2.98817	1.35385	0.69155	C	-2.97578	1.30781	0.77787
C	2.52738	1.73809	0.08826	C	2.41003	1.65651	-0.21656	C	2.40585	1.64782	-0.24533
O	0.09943	-1.18907	1.03013	O	0.07405	-0.58574	1.56196	O	0.10498	-0.73629	1.48650
O	-2.20155	0.72530	-1.07074	O	-2.14838	0.12159	-1.31848	O	-2.14152	0.21532	-1.30877
H	4.71406	-1.35446	1.11284	H	4.98723	-0.86620	1.17539	H	5.00112	-0.80923	1.20990
H	4.84726	0.36251	0.72552	H	4.93096	0.68008	0.33115	H	4.93879	0.70968	0.31717
H	5.31785	-0.86110	-0.45605	H	5.42743	-0.78613	-0.51930	H	5.44831	-0.77921	-0.48444
H	2.90191	-1.64719	-0.61860	H	3.10295	-1.74936	-0.28688	H	3.12972	-1.75102	-0.23189
H	0.63611	0.83853	-1.60757	H	0.63962	0.07509	-1.69745	H	0.66162	0.01990	-1.69919
H	0.95131	-0.89657	-1.50335	H	0.98254	-1.44454	-0.87946	H	1.00926	-1.48191	-0.84376
H	-0.22446	0.83536	0.71199	H	-0.26118	1.02831	0.40234	H	-0.23293	1.00433	0.39947
H	-1.49595	-1.27977	-1.11327	H	-1.50471	-1.66913	-0.37603	H	-1.50279	-1.64363	-0.49880
H	-4.25738	-1.50909	0.45948	H	-4.33810	-1.09653	0.98295	H	-4.34161	-1.15479	0.89231
H	-3.87505	-1.31563	-1.25957	H	-3.87318	-1.75679	-0.59417	H	-3.87697	-1.69755	-0.72891
H	-4.89918	-0.12747	-0.43596	H	-4.89211	-0.31077	-0.49927	H	-4.88709	-0.25631	-0.52745
H	-3.81321	1.49644	1.24222	H	-3.84360	1.89763	0.28224	H	-3.79460	1.90757	0.37198
H	-3.10850	0.16875	2.16933	H	-3.18083	1.17873	1.75279	H	-3.22823	1.06471	1.81324
H	-2.06971	1.44343	1.51643	H	-2.11025	1.99064	0.60146	H	-2.07655	1.92136	0.77374
H	2.49191	2.40644	-0.77858	H	1.97086	2.15046	-1.08906	H	1.96176	2.11948	-1.12769
H	3.48473	1.88451	0.58469	H	3.43867	1.99880	-0.12053	H	3.43143	2.00168	-0.15759
H	1.74178	2.07683	0.77008	H	1.86026	2.01784	0.65815	H	1.85427	2.02494	0.62181
H	1.05042	-1.19302	1.21250	H	0.43505	-1.47686	1.46332	H	-0.55695	-0.46574	2.13564

**Conformer 4**				**Conformer 5**				**Conformer 6**			
Relative energy (kcal/mol)			0.52	Relative energy (kcal/mol)			0.98	Relative energy (kcal/mol)			1.08
Population (%)			11.4	Population (%)			5.2	Population (%)			4.4
C	-4.28041	-1.35749	0.37651	C	4.05186	1.43585	-0.27048	C	-4.38448	1.10744	0.03171
C	-2.93037	-1.02690	-0.18191	C	2.68508	0.82514	-0.19016	C	-2.92762	0.93657	-0.27032
C	-2.35137	0.17176	-0.31632	C	2.35681	-0.46985	-0.11831	C	-2.23018	-0.19641	-0.40110
C	-0.95464	0.26288	-0.89865	C	0.92009	-0.95653	-0.06265	C	-0.75320	-0.13833	-0.72369
C	0.17887	0.39041	0.12435	C	-0.16051	0.07027	0.26589	C	0.16664	-0.76728	0.32669
C	1.52574	0.26405	-0.55490	C	-1.52829	-0.56930	0.22471	C	1.60794	-0.81100	-0.16106
C	2.74409	-0.29222	0.06057	C	-2.77383	0.10792	-0.16683	C	2.60236	0.27420	-0.16928
C	4.08629	0.16296	-0.45874	C	-4.07955	-0.42020	0.37462	C	3.75192	0.17305	-1.14361
C	2.76541	-0.84972	1.46214	C	-2.80871	1.54798	-0.61063	C	2.31980	1.67487	0.31377
C	-2.99629	1.47836	0.05859	C	3.35270	-1.59814	-0.10821	C	-2.81413	-1.57701	-0.26865
O	0.09217	1.67601	0.75345	O	-0.02120	0.59190	1.59373	O	0.02346	-0.07111	1.55809
O	1.97106	-1.05849	-0.89807	O	-2.09734	-0.80634	-1.07179	O	2.62595	-0.76596	0.85468
H	-4.89298	-1.87449	-0.36993	H	4.16671	2.00039	-1.20195	H	-4.52018	1.67187	0.96041
H	-4.83460	-0.48327	0.71544	H	4.85899	0.70678	-0.22184	H	-4.91871	0.16417	0.13829
H	-4.18893	-2.04306	1.22578	H	4.20124	2.15338	0.54292	H	-4.87876	1.68707	-0.75521
H	-2.35948	-1.89128	-0.51608	H	1.88129	1.55609	-0.20977	H	-2.38060	1.86929	-0.38901
H	-0.74893	-0.63687	-1.48274	H	0.67155	-1.41840	-1.02605	H	-0.57115	-0.66892	-1.66659
H	-0.88628	1.11625	-1.58243	H	0.85600	-1.76349	0.67705	H	-0.44830	0.89950	-0.87235
H	0.06418	-0.40173	0.86918	H	-0.12491	0.88553	-0.46213	H	-0.12811	-1.81487	0.47782
H	1.67098	0.99737	-1.34838	H	-1.62404	-1.41153	0.90985	H	1.76111	-1.60141	-0.89541
H	4.48675	0.96729	0.16341	H	-4.38354	0.14691	1.25795	H	3.53387	0.74009	-2.05203
H	4.00767	0.52573	-1.48401	H	-3.99354	-1.47135	0.65170	H	3.94494	-0.86372	-1.42068
H	4.80175	-0.66342	-0.43837	H	-4.86991	-0.32327	-0.37446	H	4.66145	0.58776	-0.70114
H	3.45492	-1.69660	1.51114	H	-3.59722	1.68754	-1.35482	H	3.21331	2.08505	0.79276
H	3.12184	-0.09496	2.16793	H	-3.03146	2.19953	0.23776	H	2.07994	2.31814	-0.53654
H	1.78599	-1.19582	1.78740	H	-1.86693	1.86681	-1.05288	H	1.49304	1.71199	1.01708
H	-2.40806	1.99617	0.81950	H	3.29645	-2.15243	0.83448	H	-2.52604	-2.20153	-1.12057
H	-4.01304	1.36044	0.42958	H	4.38096	-1.27014	-0.24460	H	-3.90102	-1.57200	-0.21174
H	-3.03137	2.14481	-0.80981	H	3.12117	-2.31551	-0.90238	H	-2.44134	-2.08038	0.62881
H	0.73970	1.70881	1.46957	H	0.91158	0.79983	1.73594	H	0.79699	-0.29219	2.09707

Conformer 7			
Relative energy (kcal/mol)			1.46
Population (%)			2.3
C	-3.74516	-1.52753	-0.26136
C	-2.74777	-0.49189	-0.67951
C	-2.06391	0.36434	0.08694
C	-1.10607	1.34649	-0.54944
C	0.36001	1.22199	-0.12048
C	0.98128	-0.09911	-0.52023
C	2.19785	-0.67185	0.08555
C	3.04900	-1.60113	-0.74562
C	2.92232	-0.01380	1.23497
C	-2.19128	0.45504	1.58420
O	1.05123	2.28942	-0.78811
O	0.88695	-1.19827	0.40183
H	-4.70066	-1.37572	-0.77446
H	-3.93744	-1.53638	0.81068
H	-3.39995	-2.52802	-0.54342
H	-2.56442	-0.45094	-1.75105
H	-1.14668	1.25528	-1.63764
H	-1.41467	2.36806	-0.30005
H	0.43664	1.34812	0.96242
H	0.77715	-0.37600	-1.55431
H	3.87330	-1.05524	-1.21142
H	2.45997	-2.07389	-1.53201
H	3.47984	-2.38573	-0.11783
H	3.32781	-0.77997	1.90109
H	3.76441	0.57878	0.86802
H	2.27229	0.63072	1.82434
H	-1.29080	0.08005	2.07961
H	-3.03704	-0.11047	1.97128
H	-2.31327	1.49701	1.89675
H	1.97052	2.28992	-0.49201

Conformer 8			
Relative energy (kcal/mol)			1.48
Population (%)			2.3
C	-4.27383	-1.38354	0.33939
C	-2.92374	-1.04105	-0.21086
C	-2.33758	0.15836	-0.30170
C	-0.94181	0.26072	-0.88098
C	0.19202	0.44801	0.14052
C	1.54099	0.29407	-0.53075
C	2.73533	-0.31613	0.07291
C	4.09438	0.11809	-0.41905
C	2.72532	-0.91889	1.45462
C	-2.97631	1.45168	0.12783
O	0.14791	1.71290	0.81136
O	1.94751	-1.02735	-0.92079
H	-4.88569	-1.88455	-0.41831
H	-4.82798	-0.51733	0.69830
H	-4.18189	-2.08735	1.17356
H	-2.35805	-1.89591	-0.57621
H	-0.71781	-0.65078	-1.44003
H	-0.89095	1.09211	-1.59635
H	0.08136	-0.29569	0.92867
H	1.71657	1.04720	-1.30200
H	4.51137	0.88544	0.23804
H	4.03590	0.52242	-1.43022
H	4.78565	-0.72878	-0.42503
H	3.43636	-1.74784	1.50178
H	3.02991	-0.17543	2.19523
H	1.74465	-1.30061	1.73181
H	-2.42535	1.90305	0.95668
H	-4.01365	1.33100	0.43535
H	-2.95407	2.17677	-0.69271
H	0.14168	2.41403	0.14525

Conformer 9			
Relative energy (kcal/mol)			1.52
Population (%)			2.1
C	-4.15743	0.17895	0.95267
C	-3.02404	-0.41856	0.17617
C	-2.03834	0.20702	-0.48240
C	-0.98151	-0.60539	-1.20438
C	0.24165	-1.00960	-0.35242
C	1.05119	0.16283	0.15919
C	2.52049	0.23993	0.18836
C	3.17391	1.12475	1.22178
C	3.40339	-0.85520	-0.35299
C	-1.90925	1.70224	-0.59942
O	-0.13349	-1.82516	0.75941
O	1.74900	0.96652	-0.80712
H	-5.11697	-0.07228	0.48789
H	-4.10297	1.26340	1.03617
H	-4.19102	-0.23379	1.96603
H	-3.03817	-1.50566	0.12839
H	-1.41919	-1.53561	-1.57333
H	-0.61314	-0.05462	-2.07401
H	0.86989	-1.64193	-0.97906
H	0.52770	0.74585	0.91692
H	3.47791	0.53655	2.09128
H	2.49266	1.90808	1.55574
H	4.06853	1.59767	0.80807
H	4.32817	-0.42489	-0.74609
H	3.67252	-1.55347	0.44334
H	2.92557	-1.41446	-1.15505
H	-0.90356	2.04163	-0.34132
H	-2.61858	2.24518	0.02263
H	-2.07464	2.00750	-1.63848
H	-0.88910	-1.40187	1.19318

Conformer 10			
Relative energy (kcal/mol)			1.55
Population (%)			2.0
C	-4.21784	1.39650	-0.12896
C	-2.85919	0.95555	0.32126
C	-2.33869	-0.27655	0.32440
C	-0.91890	-0.49885	0.81674
C	0.16623	-0.19892	-0.23135
C	1.53868	-0.50872	0.31594
C	2.76830	0.24163	0.01923
C	4.09218	-0.47348	0.13353
C	2.78113	1.45776	-0.87125
C	-3.06872	-1.51587	-0.12004
O	0.03688	-1.00109	-1.41156
O	2.07416	0.40410	1.28610
H	-4.77119	1.84984	0.70047
H	-4.82488	0.58530	-0.52867
H	-4.13829	2.16830	-0.90178
H	-2.22993	1.75816	0.70227
H	-0.71569	0.14385	1.67701
H	-0.79987	-1.53409	1.15013
H	0.10287	0.85971	-0.49697
H	1.65920	-1.55822	0.58497
H	4.41794	-0.83255	-0.84591
H	4.02093	-1.32713	0.80829
H	4.86008	0.20528	0.51410
H	3.55409	2.15445	-0.53635
H	3.01355	1.17107	-1.89974
H	1.82826	1.98341	-0.86614
H	-2.47176	-2.09058	-0.83393
H	-4.03567	-1.30743	-0.57508
H	-3.23792	-2.17984	0.73496
H	-0.77914	-0.74782	-1.86096

Conformer 11			
Relative energy (kcal/mol)			1.67
Population (%)			1.6
C	-3.72731	-1.53287	-0.28979
C	-2.73972	-0.47935	-0.68583
C	-2.05565	0.35919	0.09971
C	-1.10316	1.36267	-0.51160
C	0.37179	1.23363	-0.09768
C	0.99080	-0.08081	-0.52377
C	2.19284	-0.67537	0.08084
C	3.05405	-1.58128	-0.76545
C	2.90138	-0.04866	1.25549
C	-2.17076	0.40831	1.59964
O	1.13289	2.31786	-0.64883
O	0.87289	-1.20927	0.36016
H	-4.68668	-1.37476	-0.79361
H	-3.91349	-1.57111	0.78267
H	-3.37626	-2.52306	-0.59964
H	-2.56433	-0.40784	-1.75724
H	-1.15557	1.29849	-1.60360
H	-1.41408	2.37563	-0.23164
H	0.46130	1.34474	0.98164
H	0.79966	-0.32893	-1.56922
H	3.88886	-1.02295	-1.19658
H	2.47782	-2.02510	-1.57804
H	3.47047	-2.38853	-0.15701
H	3.34409	-0.82925	1.87996
H	3.70951	0.59949	0.90843
H	2.23168	0.54160	1.87783
H	-1.26406	0.02389	2.07600
H	-3.01045	-0.17176	1.97800
H	-2.29448	1.44062	1.94199
H	0.96412	2.35743	-1.59999

Conformer 12			
Relative energy (kcal/mol)			1.93
Population (%)			1.0
C	-3.74001	-1.54142	-0.25564
C	-2.74336	-0.50758	-0.67937
C	-2.06845	0.36045	0.08184
C	-1.10160	1.33084	-0.55953
C	0.36750	1.20883	-0.12124
C	0.98624	-0.11310	-0.51104
C	2.20839	-0.66828	0.09101
C	3.05356	-1.61036	-0.73103
C	2.94163	0.02254	1.21320
C	-2.20724	0.47089	1.57660
O	1.16041	2.20902	-0.77749
O	0.89964	-1.19388	0.43111
H	-4.69003	-1.40341	-0.78253
H	-3.94470	-1.53343	0.81407
H	-3.38599	-2.54447	-0.51687
H	-2.55059	-0.48055	-1.74968
H	-1.13713	1.22988	-1.64707
H	-1.41701	2.35530	-0.31897
H	0.43821	1.33259	0.96256
H	0.76921	-0.40772	-1.53737
H	3.86971	-1.06810	-1.21497
H	2.45878	-2.10042	-1.50248
H	3.49489	-2.38106	-0.09334
H	3.39263	-0.72202	1.87476
H	3.74574	0.64404	0.81246
H	2.28571	0.65258	1.81079
H	-1.30888	0.10659	2.08349
H	-3.05353	-0.09295	1.96483
H	-2.33635	1.51638	1.87472
H	0.70899	3.05880	-0.69208

Conformer 13			
Relative energy (kcal/mol)			1.55
Population (%)			2.0
C	3.46729	1.75439	-0.17457
C	2.33973	0.87160	-0.61276
C	2.01338	-0.34791	-0.17036
C	0.83374	-1.08027	-0.77290

Conformer 14			
Relative energy (kcal/mol)			1.67
Population (%)			1.6
C	4.07007	1.39387	-0.37245
C	2.71397	0.80936	-0.11517
C	2.36482	-0.47833	-0.04913
C	0.94334	-0.94131	0.20103

Conformer 15			
Relative energy (kcal/mol)			1.93
Population (%)			1.0
C	-4.59973	-0.89736	0.08586
C	-3.11808	-0.97144	-0.12092
C	-2.25331	0.02657	-0.33692
C	-0.78051	-0.28678	-0.50599

C	-0.32417	-1.37000	0.19025	C	-0.15539	0.10430	0.33093	C	0.10810	0.04413	0.70015
C	-0.97506	-0.10938	0.74129	C	-1.51925	-0.55206	0.29258	C	1.46380	-0.64069	0.58447
C	-1.94898	0.77600	0.08266	C	-2.75110	0.07508	-0.21838	C	2.63431	-0.22081	-0.20329
C	-2.06245	2.19675	0.58215	C	-4.07876	-0.39971	0.32025	C	3.64579	-1.27592	-0.58382
C	-2.45438	0.57095	-1.32376	C	-2.77086	1.46016	-0.81560	C	2.66131	1.00846	-1.07698
C	2.77174	-1.09771	0.89284	C	3.32119	-1.62930	-0.21648	C	-2.63624	1.47755	-0.44441
O	-1.25234	-2.20873	-0.49157	O	0.00112	0.77431	1.58967	O	0.22741	1.45368	0.85794
O	-2.37492	-0.21340	1.06779	O	-2.03929	-0.92236	-0.99485	O	2.56607	0.01603	1.23562
H	4.11655	2.00145	-1.02114	H	4.04741	2.04711	-1.25137	H	-5.12639	-1.49688	-0.66426
H	4.08681	1.31196	0.60430	H	4.84498	0.64637	-0.53604	H	-4.99282	0.11723	0.03868
H	3.08479	2.70729	0.20653	H	4.38457	2.02214	0.46790	H	-4.87433	-1.31488	1.06052
H	1.71610	1.29538	-1.39706	H	1.93859	1.55075	0.04788	H	-2.71061	-1.98007	-0.08411
H	0.44115	-0.52103	-1.62413	H	0.65978	-1.61291	-0.61813	H	-0.66780	-1.35561	-0.70523
H	1.16876	-2.05263	-1.15107	H	0.92737	-1.56342	1.10498	H	-0.37905	0.24789	-1.37305
H	0.06276	-1.91715	1.06006	H	-0.07457	0.81856	-0.49280	H	-0.36656	-0.37295	1.59977
H	-0.38142	0.34731	1.53080	H	-1.64389	-1.32106	1.05512	H	1.39786	-1.70491	0.80800
H	-1.45759	2.86713	-0.03335	H	-4.42241	0.25381	1.12604	H	3.45225	-1.64350	-1.59463
H	-1.72648	2.27948	1.61608	H	-4.00360	-1.41604	0.70797	H	3.61319	-2.12122	0.10422
H	-3.10003	2.53650	0.52679	H	-4.83613	-0.38542	-0.46800	H	4.65511	-0.85634	-0.56862
H	-3.50464	0.87016	-1.38313	H	-3.49779	1.49943	-1.63106	H	3.65938	1.45473	-1.05128
H	-1.89390	1.20543	-2.01474	H	-3.07816	2.19439	-0.06639	H	2.45324	0.73091	-2.11331
H	-2.36204	-0.46044	-1.65148	H	-1.80255	1.75512	-1.21574	H	1.93368	1.75290	-0.76729
H	2.17273	-1.22519	1.79988	H	3.31091	-2.26807	0.67313	H	-2.15713	2.06614	0.34120
H	3.69787	-0.60326	1.18000	H	4.34872	-1.31541	-0.38797	H	-3.71146	1.63728	-0.38576
H	3.02233	-2.10442	0.54364	H	3.01897	-2.26413	-1.05627	H	-2.28545	1.89239	-1.39515
H	-2.09075	-2.14745	-0.01072	H	-0.64506	1.49078	1.63676	H	1.00636	1.60684	1.41302

Table S5. Cartesian coordinates, energies, and population at 298 K of significantly populated conformers of model compound **3r**. Conformers were optimized at the B3LYP/6-31G(d,p)/SMD($CHCl_3$) level.

Conformer 1			
Relative energy (kcal/mol)			0.00
Population (%)			40.2
C	4.77530	1.67530	0.01984
C	3.67947	0.69086	-0.26809
C	2.61716	0.47984	0.51174
C	1.50378	-0.51043	0.23446
C	0.20432	0.29544	-0.05771
C	-1.07944	-0.52770	-0.23854
C	-2.25045	0.33693	-0.61798
C	-3.38606	0.55104	0.06776
C	-4.45453	1.46271	-0.48490
C	1.34323	-1.44393	1.45220
C	-3.71793	-0.04853	1.41176
O	-0.81495	-1.47786	-1.30207
O	1.86964	-1.28749	-0.90863
H	5.75004	1.17461	0.09223
H	4.86801	2.41339	-0.78806
H	4.60310	2.21732	0.95560
H	3.76936	0.10809	-1.18278
H	2.50256	1.05023	1.43475
H	0.02727	1.00581	0.75755
H	0.38021	0.88448	-0.96572
H	-1.28758	-1.07827	0.68556
H	-2.12748	0.83657	-1.57973
H	-5.40560	0.92604	-0.60137
H	-4.17604	1.88105	-1.45614
H	-4.65295	2.29605	0.20227
H	0.58774	-2.21209	1.25879
H	1.05224	-0.89348	2.35420
H	2.29266	-1.95042	1.65006
H	-4.68169	-0.57235	1.36916
H	-3.83096	0.74211	2.16523
H	-2.96683	-0.75083	1.77818
H	-1.58817	-2.05417	-1.38896
H	1.03751	-1.67061	-1.23848

Conformer 2			
Relative energy (kcal/mol)			0.53
Population (%)			16.4
C	-4.80311	-1.61109	-0.26738
C	-3.73769	-0.55443	-0.27508
C	-2.53833	-0.67153	0.30029
C	-1.45015	0.38645	0.30411
C	-0.11691	-0.26094	-0.15888
C	1.11970	0.66379	-0.18003
C	2.32513	-0.07606	-0.69798
C	3.39021	-0.52553	-0.01604
C	4.49715	-1.26955	-0.72365
C	-1.34481	1.01871	1.69867
C	3.60282	-0.36062	1.46866
O	0.91655	1.78738	-1.04686
O	-1.79347	1.48288	-0.57810
H	-5.72237	-1.23959	0.20387
H	-5.07673	-1.90517	-1.28917
H	-4.48361	-2.50900	0.27077
H	-3.98654	0.37533	-0.78623
H	-2.28383	-1.59036	0.82964
H	0.10072	-1.11418	0.49394
H	-0.26847	-0.66876	-1.16757
H	1.30736	1.01619	0.84391
H	2.28592	-0.25880	-1.77257
H	5.45907	-0.75491	-0.59646
H	4.30383	-1.37072	-1.79545
H	4.62821	-2.27617	-0.30386
H	-0.59895	1.81871	1.71698
H	-1.06533	0.26731	2.44316
H	-2.31093	1.44438	1.98328
H	4.57596	0.10743	1.66665
H	3.62557	-1.33975	1.96584
H	2.83312	0.24154	1.95543
H	-0.01107	2.05673	-0.92285
H	-1.96072	1.10251	-1.45583

Conformer 3			
Relative energy (kcal/mol)			0.53
Population (%)			16.4
C	4.69103	1.74347	-0.07021
C	3.60077	0.73894	-0.30165
C	2.57234	0.51960	0.52091
C	1.46539	-0.49414	0.29460
C	0.16362	0.25150	-0.09327
C	-1.09332	-0.62289	-0.29566
C	-2.27149	0.23388	-0.67993
C	-3.35578	0.54486	0.04780
C	-4.42925	1.44326	-0.51827
C	1.29720	-1.36287	1.55214
C	-3.62460	0.06787	1.45398
O	-0.90395	-1.57721	-1.34797
O	1.77327	-1.34380	-0.83510
H	5.67608	1.25952	-0.03805
H	4.73295	2.47418	-0.88852
H	4.55046	2.29054	0.86739
H	3.66418	0.15486	-1.21904
H	2.48194	1.10008	1.43995
H	-0.05010	0.99233	0.68483
H	0.36315	0.80500	-1.01866
H	-1.30558	-1.14951	0.64551
H	-2.19339	0.64163	-1.68854
H	-5.39967	0.92919	-0.53992
H	-4.19518	1.77248	-1.53470
H	-4.56730	2.33630	0.10641
H	0.55674	-2.15122	1.39157
H	0.98210	-0.75961	2.40967
H	2.24999	-1.83746	1.81345
H	-4.61395	-0.40401	1.51570
H	-3.64414	0.91570	2.15208
H	-2.88616	-0.64781	1.82059
H	0.04167	-1.80957	-1.33674
H	2.50628	-1.92545	-0.58105

Conformer 4			
Relative energy (kcal/mol)			1.33
Population (%)			4.3
C	-4.60610	-1.84899	-0.40204
C	-3.50065	-1.04187	0.21531
C	-2.68643	-0.22665	-0.45936
C	-1.53707	0.59785	0.08825
C	-0.21234	-0.02749	-0.44154
C	1.09013	0.65005	0.01171
C	2.29934	0.02402	-0.62827
C	3.30188	-0.64867	-0.03854
C	4.43818	-1.20676	-0.86048
C	-1.54781	0.70620	1.62007
C	3.40986	-0.93095	1.43954
O	0.99921	2.04115	-0.38728
O	-1.71759	1.90855	-0.47875
H	-4.45756	-2.92327	-0.22950
H	-5.57726	-1.59561	0.04303
H	-4.67401	-1.68473	-1.48208
H	-3.38095	-1.15223	1.29278
H	-2.81626	-0.11855	-1.53727
H	-0.16357	-1.07630	-0.13007
H	-0.24993	-0.01777	-1.53785
H	1.15963	0.59831	1.10380
H	2.33232	0.15419	-1.71073
H	5.40126	-0.79952	-0.52482
H	4.32415	-0.98337	-1.92491
H	4.50728	-2.29666	-0.74523
H	-0.78023	1.41062	1.95365
H	-1.36027	-0.25614	2.10834
H	-2.51532	1.08638	1.95969
H	4.37007	-0.56872	1.82916
H	3.39249	-2.01328	1.62322
H	2.61191	-0.48167	2.03371

Conformer 5			
Relative energy (kcal/mol)			1.37
Population (%)			4.0
C	4.20739	2.20834	-0.16431
C	2.91423	1.44815	-0.12059
C	2.80992	0.14541	0.15813
C	1.55280	-0.70046	0.21723
C	0.28023	0.06609	-0.20731
C	-1.03213	-0.74913	-0.20594
C	-2.18274	0.10562	-0.66969
C	-3.19503	0.61291	0.05101
C	-4.25050	1.47074	-0.60461
C	1.42365	-1.32888	1.61012
C	-3.39368	0.40876	1.53281
O	-0.95510	-1.86609	-1.10030
O	1.76174	-1.84106	-0.67572
H	4.36036	2.66884	-1.14905
H	4.20588	3.02975	0.56406
H	5.06617	1.56460	0.04958
H	2.01851	2.03092	-0.33037
H	3.71726	-0.42437	0.36716
H	0.14745	0.92408	0.46067
H	0.43423	0.46836	-1.21704
H	-1.21899	-1.10571	0.81668
H	-2.15017	0.32468	-1.73782
H	-5.24850	1.02999	-0.47778
H	-4.06828	1.59919	-1.67544
H	-4.29274	2.46695	-0.14357
H	0.63323	-2.08486	1.62931
H	1.19529	-0.56363	2.35760
H	2.36130	-1.81780	1.89061
H	-4.40053	0.02016	1.73398
H	-3.32068	1.36559	2.06723
H	-2.67156	-0.27759	1.97929

Conformer 6			
Relative energy (kcal/mol)			1.46
Population (%)			3.4
C	-4.63108	-1.78958	-0.49413
C	-3.52348	-1.02716	0.17244
C	-2.63364	-0.26332	-0.46789
C	-1.49099	0.52972	0.13566
C	-0.15429	-0.00683	-0.43427
C	1.12912	0.73819	-0.00274
C	2.34123	0.09874	-0.62855
C	3.30211	-0.63252	-0.04228
C	4.43765	-1.20396	-0.85710
C	-1.50572	0.56214	1.66664
C	3.36513	-0.96622	1.42800
O	1.11107	2.10627	-0.42786
O	-1.59803	1.89355	-0.37019
H	-4.54079	-2.86639	-0.29996
H	-5.61071	-1.48588	-0.10249
H	-4.63438	-1.63797	-1.57794
H	-3.46618	-1.12934	1.25556
H	-2.69767	-0.17016	-1.55309
H	-0.06341	-1.05803	-0.14102
H	-0.21744	0.01834	-1.52927
H	1.20582	0.69198	1.09282
H	2.40941	0.27258	-1.70323
H	5.40650	-0.83992	-0.48933
H	4.35520	-0.94263	-1.91598
H	4.46936	-2.29914	-0.77730
H	-0.73775	1.24351	2.04081
H	-1.32271	-0.42879	2.09293
H	-2.47494	0.91531	2.03521
H	4.33637	-0.67101	1.84624
H	3.28081	-2.05054	1.58159
H	2.58319	-0.48495	2.01858

H	1.77270	2.50513	-0.03500
H	-0.84342	2.33514	-0.42947

H	-0.04548	-2.20722	-1.01883
H	2.01927	-1.48001	-1.53920

H	0.18113	2.39165	-0.37546
H	-2.38238	2.29754	0.03104

**Conformer 7**			
Relative energy (kcal/mol)			1.62
Population (%)			2.6
C	-1.85388	3.18558	0.17250
C	-1.98574	1.69901	0.33383
C	-1.49639	0.78641	-0.50940
C	-1.68349	-0.71779	-0.37001
C	-0.36206	-1.50751	-0.57934
C	0.80205	-1.28279	0.40880
C	1.47854	0.06075	0.34404
C	2.70175	0.34080	-0.13883
C	3.23194	1.75370	-0.10285
C	-2.69761	-1.17716	-1.43333
C	3.64520	-0.65805	-0.76167
O	0.27160	-1.50413	1.74047
O	-2.26729	-1.06035	0.89036
H	-2.83939	3.66726	0.11913
H	-1.33828	3.63623	1.03124
H	-1.29879	3.45119	-0.73317
H	-2.52563	1.34436	1.21080
H	-0.95948	1.11065	-1.40230
H	-0.62494	-2.57139	-0.53363
H	0.02029	-1.31606	-1.58837
H	1.53588	-2.07182	0.20016
H	0.88933	0.87942	0.75105
H	4.16950	1.80537	0.46689
H	2.51847	2.44938	0.34761
H	3.46631	2.11149	-1.11437
H	-2.82739	-2.26387	-1.39310
H	-2.37373	-0.90089	-2.44193
H	-3.66603	-0.70511	-1.24227
H	4.62658	-0.61506	-0.27183
H	3.81783	-0.41196	-1.81783
H	3.28811	-1.68869	-0.71764
H	0.93216	-1.19456	2.37731
H	-1.52706	-1.09783	1.52319

**Conformer 8**			
Relative energy (kcal/mol)			1.75
Population (%)			2.1
C	-4.45956	1.95093	-0.15174
C	-3.38106	1.00184	0.28278
C	-2.59438	0.30854	-0.54282
C	-1.50479	-0.65525	-0.11766
C	-0.13354	-0.04623	-0.53187
C	1.10104	-0.93459	-0.30320
C	2.38008	-0.27524	-0.77834
C	3.19373	0.58262	-0.13860
C	4.42514	1.11868	-0.82932
C	-1.75557	-2.02452	-0.78225
C	2.99575	1.09901	1.26589
O	1.10829	-1.29391	1.09408
O	-1.56945	-0.80281	1.30358
H	-5.44043	1.63955	0.23166
H	-4.28191	2.96062	0.24195
H	-4.52908	2.02009	-1.24234
H	-3.24057	0.87409	1.35436
H	-2.71425	0.41653	-1.62185
H	-0.16031	0.21287	-1.59641
H	-0.00304	0.89037	0.02173
H	0.97953	-1.85392	-0.89387
H	2.63951	-0.53596	-1.80469
H	4.39904	2.21554	-0.87694
H	4.52929	0.73509	-1.84830
H	5.33402	0.85686	-0.27087
H	-1.02313	-2.76461	-0.44455
H	-1.70528	-1.96391	-1.87540
H	-2.74972	-2.38666	-0.50372
H	2.91241	2.19394	1.25354
H	3.87211	0.86802	1.88688
H	2.11281	0.68452	1.74944
H	1.86311	-1.87950	1.25278
H	-0.70584	-1.16433	1.56809

**Conformer 9**			
Relative energy (kcal/mol)			1.79
Population (%)			2.0
C	4.29068	2.16181	-0.15316
C	2.98062	1.43405	-0.05258
C	2.85468	0.10630	0.00239
C	1.58881	-0.72139	0.13269
C	0.32472	0.06649	-0.28833
C	-1.01341	-0.66685	-0.11527
C	-2.16634	0.14194	-0.64500
C	-3.21490	0.64060	0.03127
C	-4.28191	1.43241	-0.68488
C	1.48881	-1.25109	1.57879
C	-3.44373	0.49359	1.51527
O	-0.91512	-1.90889	-0.85792
O	1.79672	-1.83442	-0.75680
H	4.32855	2.78642	-1.05536
H	4.43525	2.83952	0.69876
H	5.13790	1.46943	-0.18360
H	2.09389	2.06653	-0.02452
H	3.75387	-0.51148	-0.02908
H	0.25524	0.99036	0.29484
H	0.44024	0.35357	-1.33994
H	-1.16049	-0.89929	0.94520
H	-2.11251	0.32354	-1.71917
H	-5.26799	0.96632	-0.55653
H	-4.08042	1.51832	-1.75631
H	-4.36364	2.44585	-0.26980
H	0.68632	-1.99087	1.66965
H	1.30206	-0.44234	2.29397
H	2.42525	-1.74449	1.85713
H	-4.43310	0.05891	1.70820
H	-3.44059	1.47805	2.00143
H	-2.69799	-0.12609	2.01690
H	-1.72329	-2.41741	-0.69702
H	0.92039	-2.24550	-0.86411

**Conformer 10**			
Relative energy (kcal/mol)			1.87
Population (%)			1.7
C	-2.44329	2.77129	-0.82489
C	-1.94957	1.62151	0.00419
C	-1.92005	0.34586	-0.38972
C	-1.45394	-0.82809	0.45819
C	-0.48346	-1.73377	-0.34010
C	0.77328	-1.06038	-0.91037
C	1.74535	-0.59709	0.14564
C	2.32347	0.61286	0.27774
C	3.32967	0.86536	1.37448
C	-2.67414	-1.65656	0.88983
C	2.05631	1.80598	-0.60614
O	1.39914	-2.07507	-1.72298
O	-0.85197	-0.39133	1.68356
H	-3.28096	3.28235	-0.33211
H	-1.65969	3.52792	-0.96363
H	-2.77995	2.44621	-1.81460
H	-1.59945	1.85818	1.00838
H	-2.28091	0.08265	-1.38562
H	-0.17550	-2.56310	0.30786
H	-1.02475	-2.17164	-1.18705
H	0.46594	-0.22051	-1.54616
H	2.04146	-1.38217	0.84307
H	4.29748	1.16614	0.95179
H	3.48828	-0.01809	1.99904
H	3.00628	1.69078	2.02215
H	-2.35544	-2.53319	1.46358
H	-3.24691	-1.99781	0.02226
H	-3.32975	-1.04755	1.51921
H	2.99601	2.17146	-1.04018
H	1.64674	2.63603	-0.01623
H	1.36207	1.60061	-1.42219
H	2.23379	-1.70156	-2.04247

**Conformer 11**			
Relative energy (kcal/mol)			1.90
Population (%)			1.6
C	-4.40846	2.01322	-0.11984
C	-3.33332	1.04656	0.28143
C	-2.60302	0.31677	-0.56499
C	-1.51424	-0.66352	-0.16800
C	-0.13218	-0.05799	-0.51931
C	1.10105	-0.94151	-0.21829
C	2.36031	-0.28426	-0.74382
C	3.22487	0.54891	-0.14131
C	4.41539	1.08383	-0.90323
C	-1.76315	-2.01557	-0.85645
C	3.13075	1.03870	1.28296
O	1.20343	-1.25357	1.17064
O	-1.49518	-0.85421	1.26637
H	-5.37427	1.73925	0.32481
H	-4.18054	3.02597	0.23752
H	-4.53469	2.05643	-1.20627
H	-3.14195	0.94771	1.34932
H	-2.76964	0.40659	-1.63918
H	-0.13185	0.19228	-1.58598
H	-0.02890	0.88477	0.02994
H	0.98772	-1.88028	-0.78699
H	2.55159	-0.52079	-1.79128
H	4.40560	2.18211	-0.92703
H	4.44554	0.72118	-1.93498
H	5.35598	0.79590	-0.41402
H	-1.03380	-2.76448	-0.53596
H	-1.70703	-1.92033	-1.94557
H	-2.76426	-2.38576	-0.60710
H	3.06706	2.13550	1.30025
H	4.04146	0.77751	1.83962
H	2.27453	0.61978	1.80754
H	0.28823	-1.30198	1.50168

**Conformer 12**			
Relative energy (kcal/mol)			1.96
Population (%)			1.5
C	4.19786	-1.89219	-0.15634
C	3.25951	-0.79170	0.24528
C	2.40765	-0.16943	-0.57391
C	1.47914	0.97650	-0.18780
C	0.02499	0.68054	-0.68033
C	-0.87034	-0.03790	0.35051
C	-2.08867	-0.63856	-0.29700
C	-3.37761	-0.30600	-0.12037
C	-4.46805	-1.02759	-0.87464
C	1.99246	2.26992	-0.83603
C	-3.87342	0.78165	0.79948
O	-0.13765	-1.04548	1.09215
O	1.49616	1.21303	1.22214
H	5.24120	-1.61026	0.03741
H	4.01319	-2.80539	0.42475
H	4.10621	-2.14073	-1.21863
H	3.29374	-0.48068	1.28884
H	2.37297	-0.45472	-1.62695
H	-0.47744	1.61992	-0.93915
H	0.07261	0.09177	-1.60535
H	-1.15500	0.68379	1.11837
H	-1.86187	-1.44077	-1.00267
H	-5.17962	-1.49521	-0.18116
H	-4.06874	-1.80625	-1.53083
H	-5.05051	-0.32830	-1.48940
H	1.35335	3.11185	-0.54984
H	1.99105	2.18973	-1.92780
H	3.01453	2.47788	-0.50555
H	-4.61024	0.37716	1.50550
H	-4.39140	1.56241	0.22690
H	-3.08167	1.26150	1.37773
H	0.30913	-1.62219	0.45300

H	-0.00377	0.02273	1.45506	H	-2.28488	-1.35991	1.51276	H	1.13929	0.39663	1.61728

**Conformer 13**				**Conformer 14**				**Conformer 15**			
Relative energy (kcal/mol)			1.97	Relative energy (kcal/mol)			2.06	Relative energy (kcal/mol)			2.09
Population (%)			1.4	Population (%)			1.2	Population (%)			1.2
C	-4.60073	1.83552	-0.09250	C	4.05795	-1.82250	0.27801	C	4.45542	-1.66497	-0.19145
C	-3.54707	0.83274	0.27596	C	3.02709	-0.76255	0.53694	C	3.41562	-0.66706	0.22802
C	-2.55753	0.43135	-0.52598	C	2.40043	-0.05021	-0.40701	C	2.50054	-0.12652	-0.57894
C	-1.48551	-0.58238	-0.17041	C	1.39717	1.06607	-0.15622	C	1.46118	0.90840	-0.17114
C	-0.09536	0.00421	-0.53551	C	-0.00382	0.74310	-0.72906	C	0.04625	0.51339	-0.72555
C	1.12213	-0.90849	-0.26083	C	-0.72444	-0.47273	-0.10894	C	-0.89466	-0.11579	0.31020
C	2.39526	-0.25832	-0.75931	C	-2.15265	-0.54627	-0.58026	C	-2.14452	-0.66470	-0.31919
C	3.27672	0.53250	-0.12503	C	-3.26909	-0.22226	0.08995	C	-3.41515	-0.27143	-0.12698
C	4.47912	1.07074	-0.86560	C	-4.62394	-0.34621	-0.56461	C	-4.54774	-0.95445	-0.85418
C	-1.77157	-1.91222	-0.88120	C	1.92503	2.35894	-0.80711	C	1.87173	2.27650	-0.73557
C	3.19271	0.96858	1.31723	C	-3.30640	0.29183	1.50798	C	-3.84882	0.85179	0.78190
O	1.21391	-1.26655	1.11839	O	-0.11288	-1.70960	-0.51025	O	-0.15875	-1.18419	0.95814
O	-1.52272	-0.89734	1.24398	O	1.19442	1.28700	1.24612	O	1.42474	1.07415	1.25019
H	-5.60441	1.39719	-0.01769	H	5.01364	-1.56427	0.75275	H	5.46833	-1.29133	0.00960
H	-4.58682	2.69397	0.59183	H	3.75141	-2.78591	0.70510	H	4.35569	-2.60333	0.37034
H	-4.46811	2.21119	-1.11189	H	4.23767	-1.96517	-0.79209	H	4.38738	-1.90126	-1.25835
H	-3.61991	0.40519	1.27578	H	2.77917	-0.57434	1.58126	H	3.42001	-0.37105	1.27647
H	-2.48528	0.83797	-1.53517	H	2.63369	-0.23221	-1.45695	H	2.48732	-0.40228	-1.63479
H	-0.09877	0.26986	-1.59925	H	-0.62597	1.63154	-0.57133	H	-0.46281	1.40484	-1.10876
H	0.04075	0.94161	0.01975	H	0.07702	0.58831	-1.81150	H	0.16196	-0.17238	-1.57261
H	0.99288	-1.82577	-0.85972	H	-0.68060	-0.37365	0.98249	H	-1.13416	0.64733	1.05892
H	2.58288	-0.45893	-1.81472	H	-2.25055	-0.90224	-1.60676	H	-1.95964	-1.48826	-1.01034
H	4.49353	2.16904	-0.84618	H	-5.27284	-1.02691	0.00288	H	-5.27041	-1.37774	-0.14382
H	4.50173	0.74823	-1.91074	H	-4.55222	-0.72004	-1.59007	H	-4.19341	-1.76005	-1.50344
H	5.41274	0.74299	-0.38840	H	-5.14105	0.62265	-0.58998	H	-5.10601	-0.23811	-1.47164
H	-1.04117	-2.67786	-0.60412	H	1.22700	3.18254	-0.62694	H	1.14576	3.04015	-0.43609
H	-1.74270	-1.78314	-1.96724	H	2.05150	2.24060	-1.88820	H	1.91531	2.25134	-1.82923
H	-2.76611	-2.27090	-0.60235	H	2.89982	2.62992	-0.38483	H	2.85773	2.56154	-0.35665
H	3.15933	2.06522	1.37775	H	-3.96836	-0.32995	2.12509	H	-4.60290	0.49614	1.49597
H	4.09455	0.66079	1.86424	H	-3.72228	1.30797	1.53792	H	-4.32831	1.65039	0.20063
H	2.32482	0.55243	1.82471	H	-2.32523	0.32136	1.98560	H	-3.02958	1.29890	1.34795
H	0.29578	-1.39730	1.41809	H	0.81153	-1.66854	-0.21979	H	-0.75734	-1.59078	1.60289
H	-1.46430	-0.05603	1.72551	H	2.04043	1.57587	1.62030	H	1.11797	0.21532	1.59360

Table S6. Cartesian coordinates, energies, and population at 298 K of significantly populated conformers of model compound **3s**. Conformers were optimized at the B3LYP/6-31G(d,p)/SMD($CHCl_3$) level.

Conformer 1				Conformer 2				Conformer 3			
Relative energy (kcal/mol)			0.00	Relative energy (kcal/mol)			0.53	Relative energy (kcal/mol)			0.53
Population (%)			40.2	Population (%)			16.4	Population (%)			16.4
C	3.09361	2.72379	0.07926	C	-2.91433	2.79761	-0.17226	C	-4.02941	-2.04285	-0.04873
C	2.71320	1.28786	0.29416	C	-2.52192	1.36236	-0.36816	C	-3.15429	-0.87117	0.28887
C	2.01847	0.53709	-0.56368	C	-2.02029	0.56849	0.58196	C	-2.35565	-0.23233	-0.56924
C	1.65700	-0.92687	-0.35483	C	-1.64068	-0.89181	0.42106	C	-1.48825	0.97176	-0.23135
C	0.12480	-1.13308	-0.51007	C	-0.10777	-1.09062	0.51868	C	-0.01201	0.70722	-0.63672
C	-0.74753	-0.31298	0.44966	C	0.73238	-0.37800	-0.56221	C	0.64085	-0.51848	0.02135
C	-2.21659	-0.54877	0.23079	C	2.20438	-0.55981	-0.29988	C	2.01799	-0.81171	-0.53743
C	-3.15056	0.33258	-0.16464	C	3.09267	0.34085	0.14880	C	3.22073	-0.33919	-0.16727
C	-4.58846	-0.09557	-0.33045	C	4.53627	-0.04455	0.36640	C	4.46652	-0.78167	-0.89791
C	2.39444	-1.78312	-1.39601	C	-2.34393	-1.72873	1.50290	C	-2.01058	2.20150	-0.99011
C	-2.89421	1.78367	-0.48833	C	2.77659	1.77794	0.48344	C	3.47455	0.64198	0.95143
O	-0.38729	-0.73389	1.79270	O	0.47449	-0.92367	-1.86259	O	0.61786	-0.29025	1.44697
O	2.09484	-1.40028	0.92128	O	-2.02314	-1.39843	-0.87588	O	-1.56974	1.30745	1.15653
H	2.69753	3.36470	0.87825	H	-2.35227	3.45529	-0.84778	H	-3.78007	-2.91663	0.56799
H	4.18424	2.85088	0.09613	H	-3.97653	2.95249	-0.40287	H	-5.08572	-1.81798	0.15008
H	2.72349	3.10684	-0.87739	H	-2.73736	3.13220	0.85472	H	-3.93759	-2.33316	-1.10045
H	3.04339	0.82549	1.22347	H	-2.65970	0.96132	-1.37220	H	-3.18674	-0.51397	1.31735
H	1.68357	0.96955	-1.50875	H	-1.86791	0.96815	1.58537	H	-2.31123	-0.56117	-1.60948
H	-0.17040	-0.87523	-1.53338	H	0.21379	-0.73816	1.50486	H	0.04133	0.57255	-1.72304
H	-0.09407	-2.19858	-0.36724	H	0.10278	-2.16649	0.47349	H	0.58063	1.59684	-0.39260
H	-0.49572	0.74709	0.33453	H	0.46988	0.68905	-0.55005	H	0.00901	-1.39244	-0.19388
H	-2.52801	-1.57496	0.43005	H	2.55587	-1.57123	-0.50780	H	1.99282	-1.49515	-1.38682
H	-5.25076	0.49852	0.31340	H	5.20456	0.57664	-0.24518	H	4.99606	0.08114	-1.32371
H	-4.73360	-1.15227	-0.08879	H	4.72349	-1.09313	0.11767	H	4.24545	-1.48071	-1.70957
H	-4.93077	0.06972	-1.36078	H	4.83561	0.11710	1.41097	H	5.17301	-1.26748	-0.21139
H	3.47435	-1.70904	-1.23503	H	-3.43214	-1.63059	1.41607	H	-3.01985	2.44606	-0.64502
H	2.17492	-1.45119	-2.41581	H	-2.05754	-1.40139	2.50763	H	-2.05038	2.01893	-2.06869
H	2.10175	-2.83406	-1.30005	H	-2.08049	-2.78515	1.39269	H	-1.36314	3.06530	-0.80571
H	-3.55244	2.43034	0.10636	H	3.44768	2.45497	-0.06128	H	3.95746	1.54497	0.55444
H	-3.13164	1.98815	-1.54068	H	2.94547	1.97142	1.55146	H	4.17736	0.21833	1.68191
H	-1.86327	2.09734	-0.31380	H	1.74842	2.06289	0.25223	H	2.56507	0.93055	1.47547
H	-0.82170	-0.13458	2.41727	H	-0.48580	-1.07621	-1.89759	H	0.94158	-1.09158	1.88428
H	1.40374	-1.13305	1.55528	H	-2.96884	-1.22467	-0.99896	H	-0.95279	0.70763	1.61345

Conformer 4				Conformer 5				Conformer 6			
Relative energy (kcal/mol)			1.33	Relative energy (kcal/mol)			1.37	Relative energy (kcal/mol)			1.46
Population (%)			4.3	Population (%)			4.0	Population (%)			3.4
C	-3.12440	2.70001	-0.17716	C	-2.30791	3.10908	-0.26012	C	-3.20972	2.70834	-0.30151
C	-2.73287	1.26047	-0.33914	C	-1.79516	1.77309	0.19711	C	-2.82645	1.39225	0.31214
C	-2.00198	0.56712	0.53687	C	-2.28094	0.59359	-0.20195	C	-2.09249	0.45409	-0.29297
C	-1.60633	-0.88828	0.41747	C	-1.86757	-0.80100	0.25516	C	-1.67564	-0.90387	0.25449
C	-0.06811	-1.05680	0.53330	C	-0.37820	-0.91621	0.66631	C	-0.16991	-0.91396	0.64424
C	0.76119	-0.32469	-0.54305	C	0.65260	-0.57413	-0.41974	C	0.80548	-0.39279	-0.41963
C	2.23590	-0.52090	-0.31218	C	2.06447	-0.72205	0.07733	C	2.23996	-0.54723	0.00452
C	3.13857	0.36540	0.13640	C	3.01812	0.22024	0.16801	C	3.16204	0.41341	0.18600
C	4.58300	-0.03382	0.32080	C	4.38994	-0.12560	0.69422	C	4.56483	0.05826	0.61537
C	-2.30874	-1.70671	1.51317	C	-2.75992	-1.22355	1.43351	C	-2.50931	-1.34871	1.45767
C	2.83901	1.79833	0.50197	C	2.84862	1.66969	-0.21482	C	2.92480	1.89255	0.00683
O	0.47378	-0.83885	-1.85049	O	0.43022	-1.50532	-1.51316	O	0.56761	-1.17412	-1.61881
O	-2.05841	-1.35751	-0.87632	O	-2.16497	-1.73482	-0.79596	O	-1.91992	-1.89257	-0.76412
H	-2.75078	3.30749	-1.01180	H	-2.67294	3.70480	0.58708	H	-4.30099	2.82006	-0.34925
H	-4.21641	2.81438	-0.17692	H	-1.51408	3.70111	-0.73457	H	-2.83910	3.55002	0.29857
H	-2.73796	3.12699	0.75389	H	-3.12772	3.00309	-0.97767	H	-2.81335	2.81431	-1.31646
H	-3.07953	0.76020	-1.24130	H	-0.97251	1.80345	0.91251	H	-3.19319	1.22025	1.32370
H	-1.64869	1.05196	1.44752	H	-3.11296	0.57524	-0.90753	H	-1.74184	0.64359	-1.30887
H	0.24489	-0.70141	1.52207	H	-0.18465	-0.26867	1.52794	H	-0.02865	-0.30603	1.54478
H	0.16878	-2.12883	0.49137	H	-0.20406	-1.94697	0.99815	H	0.09983	-1.94693	0.89841
H	0.50460	0.74335	-0.50234	H	0.46562	0.44159	-0.78130	H	0.57227	0.65738	-0.62775
H	2.57683	-1.53072	-0.54460	H	2.31335	-1.73719	0.38931	H	2.53691	-1.58372	0.16993
H	5.24428	0.59353	-0.29200	H	5.16414	0.10166	-0.05082	H	5.30086	0.41477	-0.11754
H	4.75823	-1.07911	0.05038	H	4.47307	-1.18252	0.96256	H	4.69734	-1.02044	0.73724
H	4.90239	0.10748	1.36235	H	4.63200	0.47287	1.58265	H	4.81795	0.54358	1.56745
H	-3.39410	-1.63833	1.39379	H	-3.81360	-1.18619	1.13839	H	-3.56689	-1.40746	1.18410
H	-2.04689	-1.33978	2.51047	H	-2.62053	-0.56162	2.29342	H	-2.40440	-0.66326	2.30335
H	-2.01373	-2.76195	1.45704	H	-2.52679	-2.25152	1.73171	H	-2.18407	-2.34258	1.77957
H	3.50620	2.47979	-0.04188	H	3.61914	1.96612	-0.93836	H	3.66248	2.31614	-0.68697
H	3.02711	1.97136	1.57026	H	2.98663	2.31573	0.66224	H	3.06124	2.41983	0.96020
H	1.80952	2.09512	0.29266	H	1.87277	1.89918	-0.64682	H	1.92890	2.13639	-0.36778

H	-0.49468	-0.93781	-1.88116	H	0.99285	-1.22951	-2.25158	H	1.13010	-0.82051	-2.32314
H	-1.97459	-2.32351	-0.87601	H	-1.39592	-1.71842	-1.39442	H	-1.19840	-1.78593	-1.40901

Table S7. Isotropic shieldings calculated at the mPW1PW91/6-311+G(2d,p)/SMD(CHCl3) level of theory of significantly populated conformers of model compound **1r**, average isotropic shieldings over conformers, linearly scaled calculated chemical shifts, experimental chemical shifts of compound **1**, and errors. Isotropic shieldings of methyl protons are the arithmetic mean of the shieldings of the three protons of each methyl group.

	1	2	3	4	5	6	7	8	9	10	11	12	13	14	15	16	Averag	Calcd.	Exp.	Δ
%pop	29.7	20.9	9.8	6.8	6.2	3.9	3.9	3.4	3.0	2.5	2.3	2.2	1.9	1.2	1.2	1.1				
C-10	116.0	116.7	115.1	119.2	115.8	118.1	118.1	120.3	115.9	119.9	121.3	116.7	120.5	121.0	118.2	119.5	117.0	66.0	62.1	3.9
C-11	119.0	118.8	121.6	118.6	118.0	118.8	118.9	123.3	118.7	121.0	123.1	118.6	120.8	120.6	123.7	125.7	119.6	63.6	60.4	3.2
C-12	141.4	141.1	141.4	141.3	141.4	141.0	140.9	143.0	141.6	138.0	142.7	141.2	138.3	139.3	138.1	143.1	141.2	43.1	44.1	-1.0
C-13	116.6	116.5	116.6	116.4	116.4	116.6	116.3	120.2	112.6	115.0	120.1	112.3	114.9	114.9	117.5	120.2	116.5	66.5	65.5	1.0
C-14	51.4	51.5	51.4	51.2	51.2	51.4	51.1	51.5	54.4	51.3	51.8	54.3	51.1	51.6	51.5	51.7	51.6	128.2	127.4	0.8
C-15	43.9	43.8	44.0	44.1	44.0	43.8	44.0	43.2	39.8	42.7	42.9	40.7	42.8	42.0	40.1	43.0	43.5	135.8	134.7	1.1
C-16	160.0	160.0	160.0	160.0	160.0	160.0	160.0	159.8	158.8	159.9	159.8	158.7	159.9	159.9	159.7	159.9	159.9	25.3	25.7	-0.4
C-17	168.3	168.3	168.3	168.3	168.3	168.3	168.3	168.8	167.4	168.7	168.7	167.5	168.7	168.7	168.3	168.7	168.3	17.3	18.2	-0.9
C-18	169.4	170.4	169.4	169.6	169.5	170.4	170.4	167.2	169.9	167.5	168.4	170.9	168.6	167.1	168.9	167.3	169.5	16.2	18.3	-2.1
H-10	28.85	28.84	28.90	28.61	28.93	28.61	28.61	28.04	28.81	28.90	28.02	28.82	28.92	28.88	29.06	28.08	28.77	2.77	2.98	-0.21
H-12	30.12	30.10	30.12	30.12	30.08	30.09	30.08	30.12	29.90	30.80	30.10	29.88	30.83	30.68	30.60	30.12	30.14	1.52	1.74	-0.22
H-12	30.35	30.32	30.36	30.31	30.31	30.30	30.31	29.82	30.29	29.63	29.81	30.30	29.64	29.67	29.37	29.82	30.24	1.42	1.81	-0.39
H-13	26.53	26.53	26.53	26.53	26.52	26.53	26.52	27.13	26.74	26.97	27.10	26.73	27.00	26.93	27.00	27.12	26.61	4.74	4.44	0.30
H-14	26.23	26.24	26.22	26.22	26.21	26.23	26.22	26.15	26.19	26.15	26.14	26.22	26.14	26.17	26.25	26.15	26.22	5.11	5.15	-0.04
H3-16	29.92	29.91	29.93	29.93	29.93	29.91	29.93	29.93	29.95	29.93	29.92	29.94	29.93	29.92	29.87	29.92	29.92	1.72	1.70	0.02
H3-17	29.94	29.93	29.92	29.94	29.94	29.92	29.94	29.98	29.65	29.98	29.98	29.63	29.99	29.98	29.91	29.98	29.92	1.72	1.66	0.06
H3-18	30.38	30.39	30.40	30.38	30.39	30.39	30.39	30.52	30.43	30.31	30.55	30.45	30.34	30.37	30.53	30.54	30.40	1.28	1.30	-0.02

Table S8. Isotropic shieldings calculated at the mPW1PW91/6-311+G(2d,p)/SMD(CHCl3) level of significantly populated conformers of model compound **1s**, average isotropic shieldings over conformers, linearly scaled calculated chemical shifts, experimental chemical shifts of compound **1**, and errors. Isotropic shieldings of methyl protons are the arithmetic mean of the shieldings of the three protons of each methyl group.

	1	2	3	4	5	6	7	8	9	10	11	12	13	14	15	Averag	Calcd.	Exp.	Δ
%pop	28.1	20.5	9.6	6.1	6.0	4.6	3.9	3.8	3.4	3.0	2.7	2.6	2.1	2.1	1.5				
C-10	122.1	123.0	121.3	125.5	122.1	117.6	118.4	124.2	117.6	114.9	115.5	122.1	116.0	115.6	116.7	121.2	62.0	62.1	-0.1
C-11	118.4	118.4	121.0	117.9	117.5	123.3	123.0	118.5	122.0	118.3	121.4	118.2	121.3	118.1	125.7	119.3	63.8	60.4	3.4
C-12	144.2	143.8	144.1	144.0	144.2	138.1	138.4	143.7	138.0	143.7	140.8	143.9	140.6	143.5	138.1	143.0	41.3	44.1	-2.8
C-13	118.7	118.8	118.8	118.7	118.6	117.4	117.1	118.7	117.0	114.0	116.4	114.7	116.4	113.9	117.2	118.1	65.0	65.5	-0.5
C-14	51.3	51.5	51.9	51.5	51.3	51.3	51.4	51.5	51.5	50.7	51.0	55.1	51.1	50.6	51.3	51.5	128.2	127.4	0.8
C-15	44.2	44.1	43.6	43.9	44.2	41.9	41.9	44.0	42.1	45.6	43.2	39.8	43.2	45.7	41.9	43.7	135.6	134.7	0.9
C-16	160.1	160.1	160.0	160.1	160.1	159.9	159.8	160.1	159.8	160.0	159.9	158.7	160.0	160.1	159.9	160.0	25.2	25.7	-0.5
C-17	168.4	168.3	168.3	168.3	168.3	168.6	168.6	168.3	168.6	168.8	168.7	167.5	168.7	168.8	168.6	168.4	17.2	18.2	-1.0
C-18	166.9	168.1	167.0	166.9	167.0	170.0	171.1	168.1	170.3	166.3	168.9	166.9	169.8	167.3	170.2	167.8	17.8	18.3	-0.5
H-10	28.17	28.18	28.23	27.91	28.27	28.92	28.93	27.95	28.98	28.80	29.06	28.26	29.07	28.82	28.99	28.34	3.16	2.98	0.18
H-12	29.87	29.86	29.88	29.85	29.84	30.59	30.59	29.85	30.54	30.00	29.80	29.80	29.80	29.97	30.62	29.96	1.68	1.74	-0.06
H-12	30.04	30.04	30.04	30.02	30.01	29.92	29.92	30.02	29.90	29.67	30.78	29.77	30.75	29.66	29.92	30.03	1.62	1.81	-0.19
H-13	26.95	26.95	26.97	26.94	26.92	26.92	26.91	26.94	26.88	27.17	26.71	27.18	26.72	27.16	26.93	26.95	4.44	4.44	0.00
H-14	26.27	26.27	26.29	26.27	26.26	26.18	26.20	26.26	26.17	25.82	26.13	26.26	26.14	25.82	26.18	26.23	5.09	5.15	-0.06
H3-16	29.94	29.93	29.92	29.93	29.93	29.92	29.91	29.92	29.90	29.86	29.92	29.95	29.94	29.87	29.91	29.93	1.71	1.70	0.01
H3-17	30.01	30.01	30.02	30.01	30.01	29.94	29.94	30.00	29.94	30.00	29.98	29.62	29.98	30.01	29.94	29.99	1.66	1.66	0.00
H3-18	30.45	30.48	30.47	30.46	30.49	30.41	30.43	30.48	30.44	30.45	30.27	30.43	30.30	30.47	30.43	30.45	1.24	1.30	-0.06

Table S9. Isotropic shieldings calculated at the mPW1PW91/6-311+G(2d,p)/SMD(CHCl3) level of significantly populated conformers of model compound **2r**, average isotropic shieldings over conformers, linearly scaled calculated chemical shifts, experimental chemical shifts of compound **2**, and errors. Isotropic shieldings of methyl protons are the arithmetic mean of the shieldings of the three protons of each methyl group.

	1	2	3	4	5	6	7	8	9	10	11	12	Averag	Calcd.	Exp.	Δ
%pop	27.4	26.5	13.6	11.4	5.2	4.4	2.3	2.3	2.1	2.0	1.6	1.0				
C-11	47.9	47.3	47.2	41.8	46.2	47.8	46.4	44.5	47.0	46.6	46.7	42.5	46.6	132.8	131.1	1.7
C-12	140.9	141.1	140.5	141.9	139.3	145.5	134.0	141.6	142.7	135.0	144.4	138.8	140.9	43.3	44.8	-1.5
C-13	116.3	116.0	115.6	108.3	114.5	115.9	121.6	111.3	110.8	122.1	116.3	106.8	115.0	67.9	69.2	-1.3
C-14	116.5	115.2	114.9	115.6	115.0	115.4	117.7	117.3	115.1	121.4	116.7	115.2	115.8	67.1	67.5	-0.4
C-15	126.2	123.2	121.4	122.5	125.0	122.8	123.9	123.3	121.3	123.9	126.0	124.5	123.8	59.6	58.3	1.3
C-16	162.6	162.6	162.9	162.7	162.6	162.6	162.7	162.6	162.7	162.9	162.6	162.7	162.7	22.7	24.9	-2.2
C-17	168.4	168.6	167.9	168.3	168.7	168.5	170.0	168.7	168.1	169.9	168.5	168.6	168.5	17.2	19.4	-2.2
H-12	29.71	29.56	29.33	29.60	29.78	29.55	29.48	29.55	29.21	29.26	29.69	29.78	29.57	2.04	2.23	-0.19
H-12	29.62	29.62	29.47	29.68	29.48	29.75	29.58	29.17	29.74	29.51	29.57	29.58	29.59	2.02	2.10	-0.08
H-13	28.23	27.97	28.01	28.29	28.23	27.80	27.60	28.05	28.33	27.73	27.80	28.54	28.09	3.39	3.51	-0.12
H-14	29.17	29.14	29.18	29.13	29.34	29.04	29.08	29.10	29.02	28.88	29.07	29.32	29.15	2.43	2.66	-0.23
H3-16	30.59	30.58	30.63	30.57	30.60	30.57	30.56	30.60	30.55	30.60	30.58	30.59	30.59	1.11	1.09	0.02
H3-17	30.59	30.57	30.70	30.57	30.60	30.60	30.39	30.60	30.71	30.43	30.63	30.59	30.59	1.11	1.07	0.04
H3-18	30.09	30.08	30.10	29.87	30.08	30.09	30.03	29.87	30.09	30.05	30.05	29.87	30.05	1.60	1.52	0.08

Table S10. Isotropic shieldings calculated at the mPW1PW91/6-311+G(2d,p)/SMD(CHCl3) level of significantly populated conformers of model compound **2s**, average isotropic shieldings over conformers, linearly scaled calculated chemical shifts, experimental chemical shifts of compound **2**, and errors. Isotropic shieldings of methyl protons are the arithmetic mean of the shieldings of the three protons of each methyl group.

	1	2	3	4	5	6	7	8	9	10	11	12	13	14	15	Averag	Calcd.	Exp.	Δ
%pop	61.1	8.8	8.2	3.4	3.0	2.3	2.0	1.9	1.8	1.4	1.3	1.2	1.2	1.2	1.1				
C-11	46.8	46.0	46.7	42.1	46.2	46.7	46.9	43.0	47.5	45.2	46.9	47.3	46.8	46.6	41.4	46.4	133.1	131.1	2.0
C-12	139.2	137.4	139.6	141.6	142.7	141.3	137.2	138.0	143.9	142.4	134.0	133.6	139.7	144.5	142.2	139.3	44.8	44.8	0.0
C-13	117.7	116.5	116.6	107.6	117.8	118.9	114.5	108.2	116.8	108.3	114.4	114.1	118.0	116.1	110.9	116.6	66.4	69.2	-2.8
C-14	118.1	116.9	117.4	117.6	118.5	117.4	117.6	117.0	119.7	117.9	117.2	118.8	118.4	117.8	117.6	117.9	65.2	67.5	-2.3
C-15	123.9	123.7	124.2	124.0	123.7	123.1	126.9	123.2	122.8	123.9	126.2	126.3	123.0	123.9	123.0	123.9	59.4	58.3	1.1
C-16	162.8	162.8	162.8	162.8	162.8	161.9	162.8	162.8	162.7	162.9	162.8	162.8	162.0	162.7	162.0	162.8	22.6	24.9	-2.3
C-17	169.2	168.9	169.6	169.6	169.1	168.6	169.6	168.8	169.2	169.0	168.8	168.8	168.7	169.7	168.6	169.2	16.5	19.4	-2.9
H-12	29.26	29.14	29.36	29.41	29.26	29.45	29.51	29.23	29.39	29.34	29.67	29.37	29.52	29.51	29.52	29.29	2.29	2.23	0.06
H-12	29.73	29.81	29.58	29.68	29.67	29.64	29.14	29.89	29.35	29.82	29.04	29.32	29.12	29.62	29.70	29.68	1.94	2.10	-0.16
H-13	28.35	28.22	28.01	28.32	27.80	27.24	27.94	28.55	28.31	28.27	28.18	27.95	27.21	27.82	27.62	28.23	3.27	3.51	-0.24
H-14	29.27	29.38	29.23	29.23	29.20	29.04	29.25	29.38	29.36	29.20	29.39	29.28	29.11	29.16	29.02	29.26	2.32	2.66	-0.34
H3-16	30.55	30.55	30.55	30.57	30.54	30.58	30.60	30.56	30.56	30.56	30.60	30.60	30.61	30.54	30.58	30.56	1.14	1.09	0.05
H3-17	30.52	30.52	30.54	30.57	30.49	30.34	30.56	30.54	30.57	30.54	30.54	30.54	30.33	30.51	30.34	30.52	1.17	1.07	0.10
H3-18	30.08	30.06	30.07	29.87	30.03	30.03	30.11	29.88	29.97	29.96	30.10	30.10	30.00	30.07	29.89	30.06	1.60	1.52	0.08

Table S11. Isotropic shieldings calculated at the mPW1PW91/6-311+G(2d,p)/SMD(CHCl3) level of significantly populated conformers of model compound **3r**, average isotropic shieldings over conformers, linearly scaled calculated chemical shifts, experimental chemical shifts of compound **3**, and errors. Isotropic shieldings of methyl protons are the arithmetic mean of the shieldings of the three protons of each methyl group.

	1	2	3	4	5	6	7	8	9	10	11	12	13	14	15	Averag	Calcd.	Exp.	Δ
%pop	37.8	15.4	15.3	4.0	3.7	3.2	2.4	2.0	1.8	1.6	1.5	1.4	1.3	1.2	1.1				
C-10	40.9	39.2	40.0	38.7	40.5	40.3	37.7	41.1	38.7	40.2	40.3	36.3	39.2	38.7	35.8	40.0	139.1	139.0	0.1
C-11	110.1	106.3	106.4	110.4	107.7	107.2	108.5	110.0	110.4	110.4	106.2	110.5	106.1	109.8	111.2	108.5	74.1	73.0	1.1
C-12	139.2	137.1	139.2	139.0	141.5	139.4	139.1	139.0	143.4	136.7	139.1	138.3	137.1	135.3	139.0	138.9	45.2	47.6	-2.4
C-13	115.2	117.1	117.6	114.7	117.4	117.1	111.4	111.8	114.9	118.0	113.6	115.0	113.4	118.0	115.7	115.9	67.1	66.3	0.8
C-14	52.5	50.9	50.4	52.3	50.5	50.3	52.5	53.8	52.3	51.1	54.1	52.8	54.3	51.7	53.0	51.8	127.9	127.8	0.1
C-15	41.4	44.0	44.5	41.6	44.2	44.4	41.7	36.9	41.5	37.3	39.9	42.5	40.1	44.7	40.8	42.4	136.8	134.6	2.2
C-16	159.8	160.0	160.0	159.9	160.0	160.1	159.8	159.0	159.9	159.6	158.9	160.0	158.9	160.1	159.9	159.9	25.3	25.7	-0.4
C-17	168.7	168.4	168.5	168.7	168.4	168.5	168.9	167.2	168.6	168.0	167.5	168.7	167.5	168.2	168.6	168.5	17.1	18.2	-1.1
C-18	160.5	162.8	160.5	163.8	162.3	164.4	155.1	160.9	158.8	156.7	160.8	156.6	163.1	154.5	155.4	160.8	24.4	27.0	-2.6
H-12	30.34	30.31	30.44	30.57	30.14	30.61	29.40	30.14	30.01	30.00	30.22	30.00	30.07	30.14	30.20	30.31	1.37	1.52	-0.15
H-12	30.19	30.34	30.19	29.94	30.07	29.94	30.10	30.07	30.06	29.70	30.13	30.17	30.25	30.07	30.00	30.17	1.49	1.78	-0.29
H-13	26.59	26.62	26.72	26.57	26.58	26.65	26.62	26.89	26.53	27.13	26.92	26.93	26.85	26.65	26.63	26.65	4.71	4.74	-0.03
H-14	26.14	26.23	26.19	26.14	26.15	26.18	25.61	26.01	26.06	26.09	26.20	26.36	26.17	26.23	26.20	26.15	5.17	5.19	-0.02
H3-16	29.91	29.92	29.93	29.91	29.90	29.93	29.91	29.92	29.88	29.86	29.95	29.92	29.94	29.94	29.90	29.92	1.73	1.69	0.04
H3-17	29.95	29.97	29.97	29.98	29.96	29.98	30.01	29.64	29.94	30.00	29.60	29.98	29.62	29.99	29.95	29.94	1.70	1.67	0.03
H3-18	30.44	30.39	30.35	30.31	30.38	30.21	30.70	30.52	30.44	30.64	30.42	30.59	30.46	30.56	30.58	30.42	1.26	1.37	-0.11

Table S12. Isotropic shieldings calculated at the mPW1PW91/6-311+G(2d,p)/SMD(CHCl3) level of significantly populated conformers of model compound **3s**, average isotropic shieldings over conformers, linearly scaled calculated chemical shifts, experimental chemical shifts of compound **4**, and errors. Isotropic shieldings of methyl protons are the arithmetic mean of the shieldings of the three protons of each methyl group.

	1	2	3	4	5	6	Average	Calcd.	Exp.	Δ
%pop	79.0	7.3	4.2	4.1	1.1	1.0				
C-10	41.1	42.4	41.6	46.0	38.0	40.5	41.4	137.8	138.2	-0.4
C-11	109.0	105.7	108.9	106.1	109.5	110.5	108.6	74.0	73.6	0.4
C-12	139.4	139.1	139.1	136.3	142.5	136.5	139.2	44.9	47.8	-2.9
C-13	114.1	116.8	110.6	116.2	115.2	114.7	114.3	68.6	67.5	1.1
C-14	52.4	50.5	53.8	51.0	52.4	52.4	52.2	127.5	129.5	-2.0
C-15	41.9	44.7	37.7	44.3	41.8	41.1	42.1	137.2	132.7	4.5
C-16	160.0	160.1	158.9	160.1	159.9	159.9	160.0	25.2	25.6	-0.4
C-17	168.7	168.4	167.4	168.3	168.6	168.6	168.6	17.1	18.1	-1.0
C-18	156.8	154.9	157.0	154.9	154.7	161.0	156.6	28.4	31.0	-2.6
H-12	30.33	30.44	30.13	30.26	29.93	30.41	30.32	1.35	1.48	-0.13
H-12	29.98	29.93	29.89	30.18	29.96	29.92	29.98	1.67	1.79	-0.12
H-13	26.82	26.97	27.13	26.89	26.57	26.66	26.85	4.53	4.62	-0.09
H-14	26.13	26.17	26.11	26.22	26.09	26.14	26.14	5.18	5.18	0.00
H3-16	29.92	29.94	29.93	29.94	29.89	29.91	29.92	1.72	1.68	0.04
H3-17	30.00	30.04	29.63	30.02	29.96	29.99	29.99	1.66	1.61	0.05
H3-18	30.65	30.52	30.64	30.48	30.63	30.52	30.63	1.07	1.25	-0.18

Table S13. Calculated relative energies (ΔE), populations, and optical rotations of significantly populated conformers of model compounds **1s** and **2r** at the B3LYP/TZVP/SMD(CHCl3) level.

	model compound **1s**			model compound **2r**		
Conformer	ΔE (kcal/mol)	% population	$[\alpha]_D$	ΔE (kcal/mol)	% population	$[\alpha]_D$
1	0.00	28.1	+42.8	0.00	27.4	–9.3
2	0.19	20.5	+28.3	0.02	26.5	+0.2
3	0.64	9.6	+55.0	0.42	13.6	–39.8
4	0.91	6.1	+26.1	0.52	11.4	–3.7
5	0.91	6.0	+18.9	0.98	5.2	–4.3
6	1.07	4.6	–87.9	1.08	4.4	+21.9
7	1.17	3.9	–48.5	1.46	2.3	+55.0
8	1.19	3.8	+66.0	1.48	2.3	–12.4
9	1.25	3.4	–58.8	1.52	2.1	+64.9
10	1.32	3.0	–53.3	1.55	2.0	–4.4
11	1.39	2.7	–83.2	1.67	1.6	–8.0
12	1.42	2.6	–5.0	1.93	1.0	–4.7
13	1.52	2.1	–84.7			
14	1.54	2.1	-–0.1			
15	1.73	1.5	-80.9			
		**weighted mean**	**+13.3**		**weighted mean**	**–5.5**
		**experimental value**	**+17.0**		**experimental value**	**–19.5**
